# Mannich reaction with organozinc reagents in continuous flow: experimental and computational studies

**DOI:** 10.1039/d6ra01038e

**Published:** 2026-03-16

**Authors:** Lucas Fraile-González, Ángel Sánchez-González, Laura F. Peña, Enol López

**Affiliations:** a Department of Organic Chemistry, School of Engineering (EII) University of Valladolid (UVa). Dr Mergelina 47002 Valladolid Spain enol.lopez@uva.es; b BioISI─BioSystems and Integrative Sciences, Institute Faculdade deCiências da Universidade de Lisboa, Campo Grande Lisbon 1749-016 Portugal; c Faculty of Pharmacy, University of Castilla-La Mancha. Calle Almansa 14 - Edif. Bioincubadora 02008 Albacete Spain

## Abstract

Herein, we report a fully continuous flow version of a Mannich reaction using organozinc reagents. These organometallics were generated *in situ* in a packed-bed reactor and subsequently introduced in a 1 mL chip to react with an array of substituted aldehydes and primary amines. The highly substituted amine products were obtained in moderate to good yields within 5 minutes and under mild conditions, thereby reducing reaction times. DFT studies suggest the participation of organometallic dimers as nucleophiles, responsible for C–C bond formation through an S_N_1 reaction pathway.

## Abstract 2

Fully continuous flow version of a Mannich reaction using organozinc reagents.

## Graphical abstract

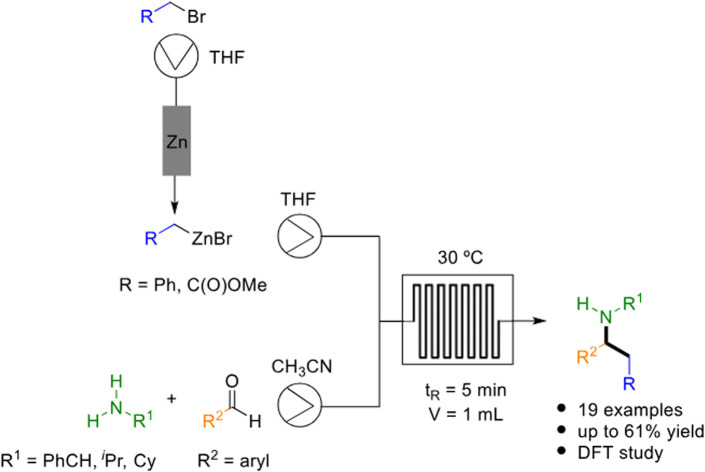

## Introduction

Over the last three decades, continuous flow has gained significant attention in organic synthesis in both academic and industrial environments due to the numerous benefits compared to batch methodologies.^1–3^ The improved heat and mass transfer processes in flow as well as the superior mixing efficiency (deriving from enhanced surface-to-volume ratios) enable safer handling of hazardous reagents by significantly reducing reaction times. In addition, critical reaction parameters (*e.g.* residence time, temperature, concentrations) are precisely controlled to facilitate reproducibility and process scalability.^4,5^ From a technological perspective, chip microreactors have been widely implemented for the preparation of organic molecules in flow, as rapid screenings and scope studies can be carried out with minimum amounts of materials by reducing chemical waste.^6,7^

Within this framework, organozinc agents are very attractive intermediates in organic synthesis,^8,9^ which present a broader functional-group compatibility and higher chemoselectivity than the corresponding Grignard counterparts.^10^ Nevertheless, their preparation remains challenging: (i) solutions tend to decompose readily and limit their commercial availability; (ii) residual zinc must be removed upon reaction completion and (iii) the exothermicity of these transformations hinders scale-up processes. In 2014, a continuous flow approach was reported that effectively overcame these limitations, providing enhanced safety, improved product quality and greater efficiency^11–14^ with the use of packed-bed reactors.^15^ This strategy of immobilizing zinc metal in a single column allowed a rapid and reproducible preparation of organozinc species, while avoiding direct handling and zinc waste.^16^ Although the initial setup of the packed-bed reactor requires immobilization of zinc metal, with minor additional operational steps (*e.g.* column preparation) and cartridge costs compared to direct use of zinc powder, this approach offers significant advantages in terms of safety (no direct handling of fine powders), waste minimization (no residual zinc dust), and seamless integration into fully continuous workflows, facilitating scale-up and reproducibility. Once prepared, the organometallic species can subsequently participate in a variety of synthetic reactions^17^ or even in combination with novel technologies such as photo- or electrochemistry.^18,19^ Thus, the development of synthetic reactions involving organozinc agents is highly desirable from a synthetic perspective, as C(sp^3^)–fractions can easily be installed into organic frameworks in a reproducible and sustainable manner by using the benefits of continuous flow.

**Fig. 1:**
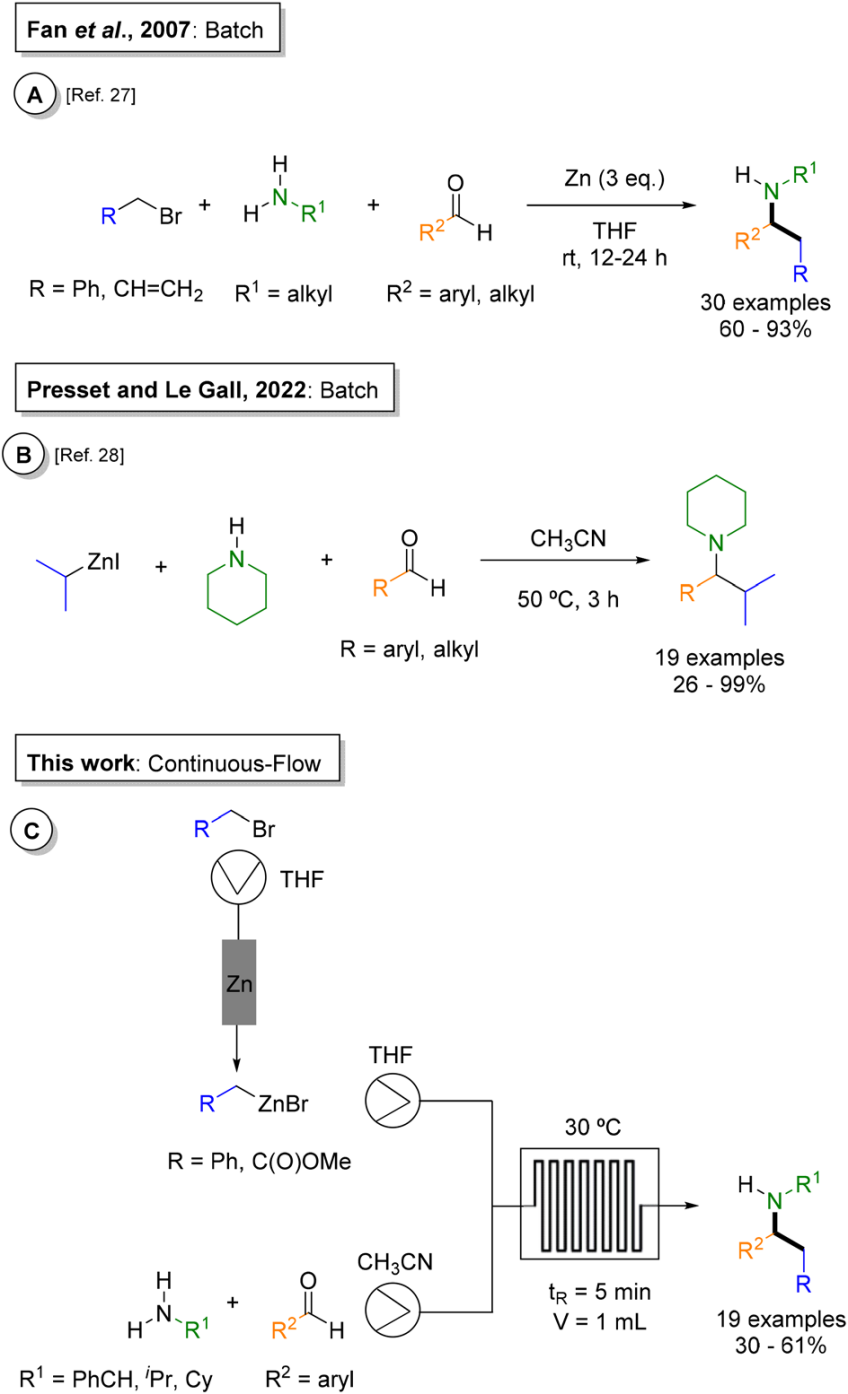
Representative examples of organozinc Mannich reactions. (A) and (B) Previously reported protocols under batch conditions; (C) continuous-flow version presented herein.

In organic synthesis, the Mannich reaction is a classical multicomponent transformation which constitutes a key strategy for the preparation of numerous pharmaceuticals and natural products.^20,21^ This transformation enables the simultaneous formation of two new C–C and C–N bonds in a single step,^22,23^ by combining a nucleophile, a carbonyl compound and an amine derivative in a one-pot process. To improve regio- and stereoselectivity, a variety of modifications have been developed, including the use of preformed iminium salts and imines as electrophiles, or electron-rich nucleophiles such as enolates and silyl enol ethers.^22^ Beyond these classical approaches, organometallics (organoboron, organosilicon, and organozinc derivatives) have been employed to further expand the synthetic versatility of the Mannich reaction.^24–26^ In 2017, Fan and coworkers developed a three-component batch process for the benzylation and allylation of aromatic and aliphatic aldehydes with amines, using commercially available zinc powder to generate the organozinc (Fig. 1A).^27^ More recently, Presset, Le Gall, and coworkers reported the preparation of mixed alkylzinc reagents in batch and applied them in a multicomponent version involving a range of amines and aldehydes in acetonitrile at 50 °C for 3 hours (Fig. 1B).^28^ The same group also described the generation of secondary benzylzinc species *via* direct metalation of (1-bromoethyl)benzenes with zinc dust in tetrahydrofuran, granting access to a diverse array of α,β-disubstituted β-arylethylamines through a Mannich reaction.^29^ In all these studies, the authors proposed a putative mechanism for the organometallic Mannich reaction in which the reaction pathway is governed by the nature of the amine component. When primary amines were used, hemiaminal^27^ or imine intermediates were postulated,^24^ whereas reactions with secondary amines were proposed to proceed through the formation of alkoxide intermediates, accompanied by a phenyl transfer *via* a six-membered transition state.^24,28^ Despite these efforts, previous organozinc Mannich mechanisms^30–32^ were proposed solely from experimental observation without computational validation. In addition, their implementation under continuous-flow conditions remained underexplored.

In this work, we developed a fully continuous-flow protocol integrating both the generation of organozinc agents^15^ and subsequent Mannich reaction with an array of amine and aldehyde partners (Fig. 1C). While batch organozinc Mannich reactions are viable (Fig. 1A and B), they suffer from exothermic zinc activation, powder handling/waste, and long residence times that limit throughput. Our integrated flow platform eliminates these *via* safe packed-bed organozinc generation with 5 min residence time in a microchip reactor. Additionally, it enables future automated synthesis capabilities. Finally, computational studies were performed to gain deeper insight into the reaction mechanism, revealing a different pathway involving dimeric nucleophiles across amine derivatives.

## Results and discussion

**Scheme 1:**
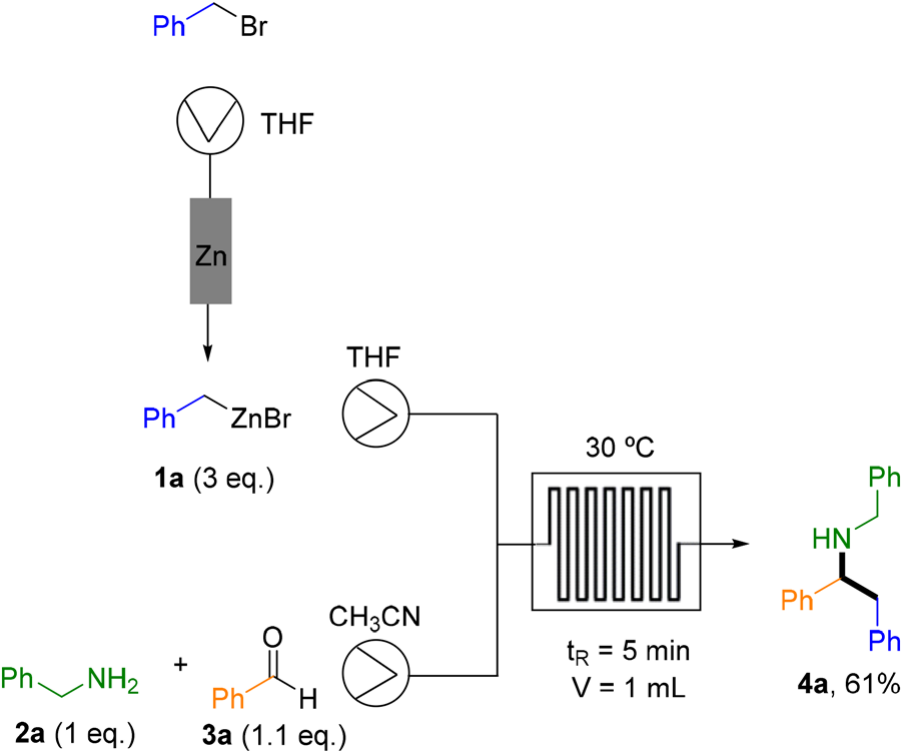
Continuous-flow setup under optimized reaction conditions.

The three-component Mannich reaction under flow conditions was studied through the system depicted in Scheme 1. Benzylzinc bromide (1a) was prepared in a packed-bed reactor containing Zn and pumping the corresponding halide in THF.^15^ Subsequently, a systematic optimization was conducted to determine the ideal stoichiometry, temperature (T), and residence time (*t*_R_) of the Mannich reaction within the flow reactor (Table 1). Based on previous investigations from Presset *et al.*,^28^ CH_3_CN was selected as solvent for the Mannich step, while THF was employed for the generation of organozinc species. After evaluating the influence of residence time and temperature on the reaction outcome, the optimal conditions were identified as those described in entry 2 using 3 eq. of 1a (30 °C, 5 min residence time). Although an identical yield was obtained at 40 °C with a 10 min residence time, the former conditions were selected for the scope studies due to the shorter residence time.

**Table 1:** Screening of the reaction parameters

Entry	*T* (°C)	*t*_R_ (min)	Flow (mL min^−1^)	NMR yield of 4a^a^^,^^b^
1^c^	30	5	0.2	42%
2^d^	30	5	0.2	76%
3	30	10	0.1	52%
4	40	5	0.2	48%
5	40	10	0.1	76%
6	50	10	0.1	39%

a The reaction was analysed by ^1^H-NMR using 1 eq. of DCE as internal standard and 0.5 mL of CDCl_3_.

b Literature batch protocol (50 °C, 3 h)^28^ gave 54% yield.

c 2 eq. of the amine were used instead of 1 eq.

d 3 eq. of organozinc optimal, 2 eq. reduced the yield to 60%.

Under these conditions, three equivalents of preformed benzyl zinc bromide were introduced into a 1 mL microchip flow reactor and combined with a solution of benzylamine (2a) and benzaldehyde (3a). The two streams were allowed to react for 5 min at 30 °C, affording the corresponding secondary amine 4a in a satisfactory isolated yield of 61%, with a productivity of 105 mg h^−1^, demonstrating the enhanced throughput of the continuous flow protocol *versus* traditional batch methods (Scheme 1).

The generality of the transformation was then evaluated by examining a series of aromatic aldehydes bearing substituents with varying electronic properties and substitution patterns (Table 2). The reaction efficiency was significantly influenced by the electronic nature of the substituents and ^1^H-NMR analysis of crude reaction mixture confirmed Wurtz-type coupling, and hydrolysis byproducts arising from the excess of organozinc reagent. *Para*-substituted aldehydes containing strong electron-withdrawing groups (EWGs) provided the highest yields within the series. In particular, the nitro derivative (4b, *p*-NO_2_) exhibited the best performance, indicating that electron deficiency on the aromatic ring may enhance the reaction efficiency. Halogen-substituted aldehydes afforded intermediate yields, with *p*-Cl (4c) and *p*-Br (4d) derivatives giving 41% and 43%, respectively. These results suggest that the combined electronic effects of halogens provide a moderate influence on the reaction efficiency, without significantly enhancing or diminishing the yield. The *para*-methyl-substituted derivative (4e, *p*-Me) afforded a moderate yield of 46%, comparable to those of the halogenated counterparts, indicating that a mild electron-donating effect does not significantly affect the reaction outcome. In contrast, the strongly electron-donating methoxy group (4f, *p*-OMe) resulted in the lowest yield of the series (30%), likely due to excessive electron density on the intermediate, which disfavours the reaction outcome. *Meta*-substituted aldehydes, both the cyano (4g, *m*-CN) and methyl (4i, *m*-Me) derivatives, were well-tolerated and afforded the corresponding products in comparable yields. In contrast, the *meta*-chloro derivative (4h, *m*-Cl) gave a slightly lower yield, consistent with the different electronic effects of chlorine at the *meta* position *versus para* position. A direct comparison for the three methyl isomers (4e, 4i and 4j) revealed a modest decrease in yield for the *ortho* derivative (4j, *o*-Me), which can be attributed to increased steric congestion near the reaction site. This steric effect became even more pronounced in the *ortho*-bromo compound (4k, *o*-Br), where the bulky bromine atom blocks access to the reactive centre and further diminishes the reaction efficiency. Such behaviour is characteristic of the well-known steric “*ortho* effect” commonly observed in substituted aromatic systems.^30^ Other aromatic systems, such as the naphthyl group (4l) and pyridine (4m), also delivered the desired products in comparable yields, indicating that extended polycyclic and electron-withdrawing heteroaromatic frameworks are compatible with the transformation. Vinylic substituents (4n) were also tolerated, providing the corresponding Mannich adduct, albeit in slightly reduced yield. In contrast, aliphatic aldehydes failed to furnish the corresponding products, which suggest the high sensitivity of the reaction to the electronic and structural nature of the carbonyl component. To further expand the applicability of the methodology, we next examined the scope with respect to both the amine and the organozinc partners. Under similar reaction conditions, benzylzinc bromide 1a reacted efficiently with two different primary amines (isopropyl and cyclohexyl amines) in presence of benzaldehyde, affording compounds 4o and 4p (40% and 44%, respectively). Unfortunately, secondary amines failed to provide the expected Mannich adducts in flow and batch conditions were needed (see Table 3). To increase the scope in the organometallic, the organozinc agent 1b resulting from the Reformatsky reagent (R = CH_2_CO_2_Me) was prepared and the Mannich reaction was carried out with three different aromatic aldehydes and benzylamine, giving rise to the corresponding ester derivatives in comparable yields (4q–s).

**Table 2:** Reaction scope under optimized flow conditions^a^

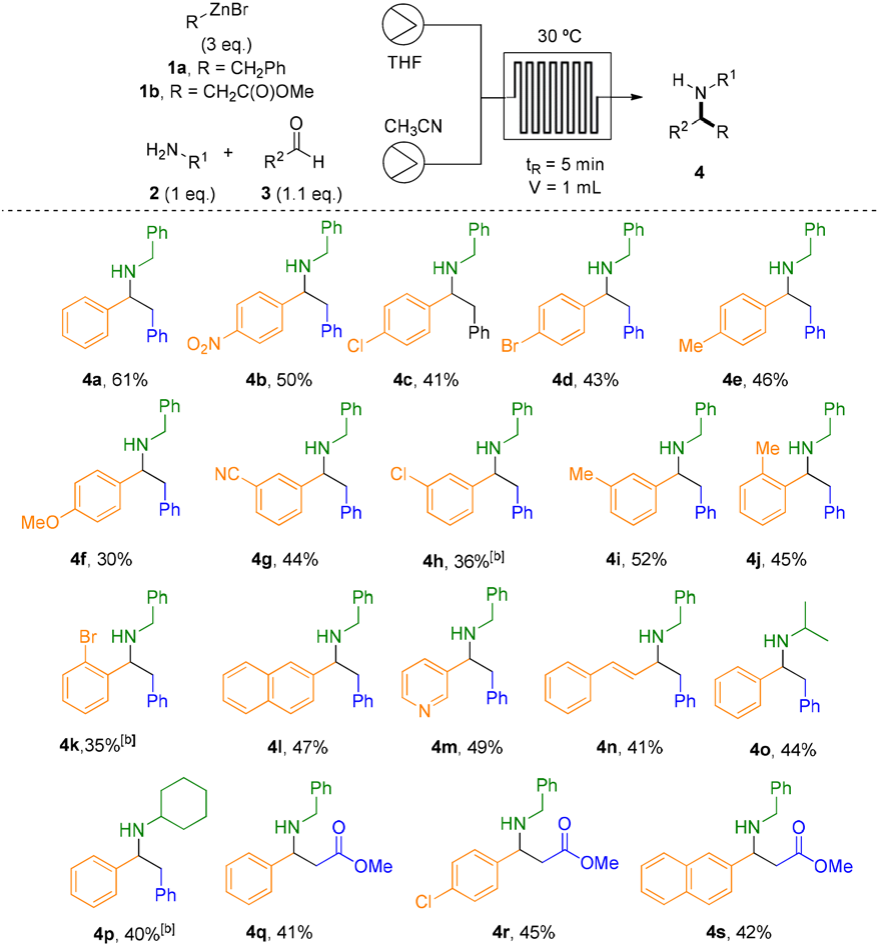

a Reaction conditions: 1a (3 eq.); amine 2 (1 eq.); aldehyde 3 (1.1 eq.), THF : CH_3_CN 1 : 1; 30 °C, 0.2 mL min^−1^, 5 min.

b Batch conditions were used because of solubility issues: 1a (3 eq.); amine 2 (1 eq.); aldehyde 3 (1.1 eq.), THF : CH_3_CN 1 : 1; rt, 24 h.

**Table 3:** Organometallic Mannich reaction in batch to introduce secondary amines

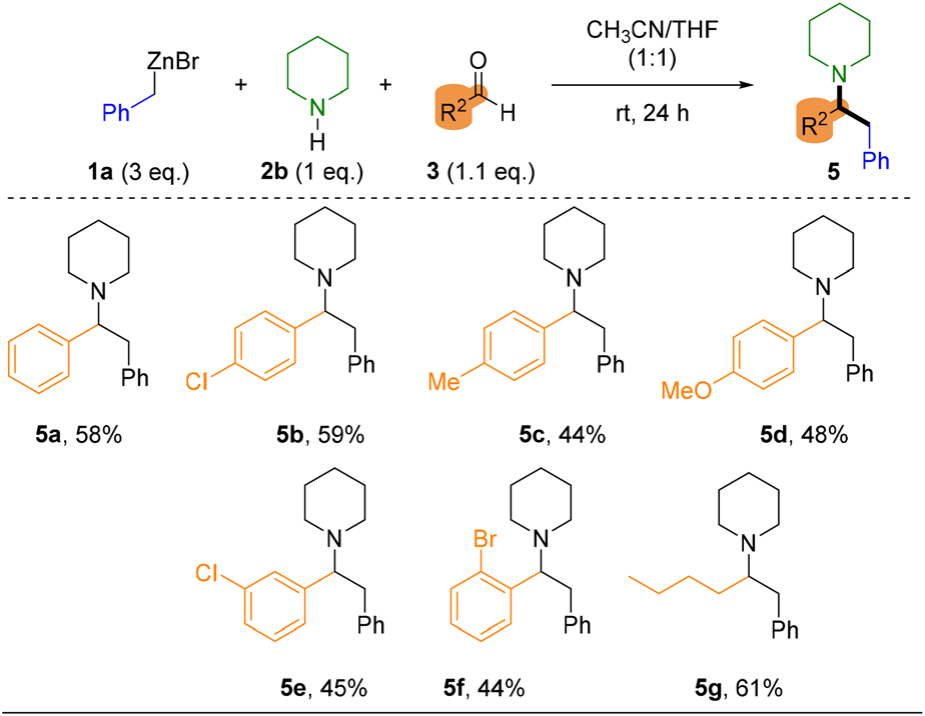

Finally, due to the inability to introduce secondary amines under the established flow conditions, the methodology was applied in batch mode. In this approach, the organozinc reagent 1a was first generated in the continuous-flow system and subsequently used in a conventional Mannich reaction at room temperature in a mixture of THF/CH_3_CN. The reactivity pattern remained consistent with our previous observations, aromatic aldehydes were well tolerated, and only minor variations in yield were observed, which can be attributed to electronic and steric effects. Notably, strong electron-donating substituents (5d, *p*-OMe) did not hamper the reaction outcome and the reaction with aliphatic aldehydes furnished the desired adduct (5g), in agreement with analogous reported reactions of piperidine with alkylic aldehydes.

In order to gain deeper insight into the reaction mechanism, a DFT computational study of the three-component Mannich reaction was performed at a fundamental level.

## Computational studies

### Computational details

Density functional theory (DFT) calculations were performed with Gaussian 09 package.^33^ To reproduce accurately the reaction profiles, HSEH1PBE^34^ hybrid functional has been employed, with 6-311++G(d,p) basis set to compute geometries and energies. This functional has described accurately energies and geometries (in comparison with CCSD methods) in a benchmark study, considering also the noncovalent interactions.^35,36^ To properly describe the reaction environment for the studied reactions, solvent effects have been considered, performing Self-Consistent-Reaction field (SCRF) within the Polarization Continuum Model (PCM).^37^

Stationary points and transition states (TSs) were characterized by calculating the eigenvalues of the second derivative matrix (Hessian). None of the optimized minima showed any imaginary frequencies, indicating a real minimum of potential energy surface (PES), while the geometries corresponding to TSs presented a single-point imaginary frequency. TSs optimized geometries were found from a relaxed scan along the reaction coordinates between the involved carbon atoms. The reaction profiles were determined by following a fractional displacement of the imaginary vibrational mode to both, reactants and products. Molecular renderings were made with Chemcraft.^38^ To gain insight into the proposed mechanism and describe the reaction profile at fundamental level, the wave functions were obtained to analyse the topology of electron density with quantum theory of atoms in molecules (QTAIM)^39^ using AIMALL software package.^40^ This topological tool provides an accurate description of the bonding scheme in the optimized structures, intermediates and transition states.

The structure of benzylzinc bromide 1a was optimized, and its wave function analysed with the QTAIM methodology. The QTAIM analysis has been proven useful in the description of similar organometallic compounds to describe future reactivity and applications.^41^ This methodology allows to understand, at fundamental level, the nature of the chemical bond and, consequently, the structure and chemical reactivity.

**Fig. 2:**
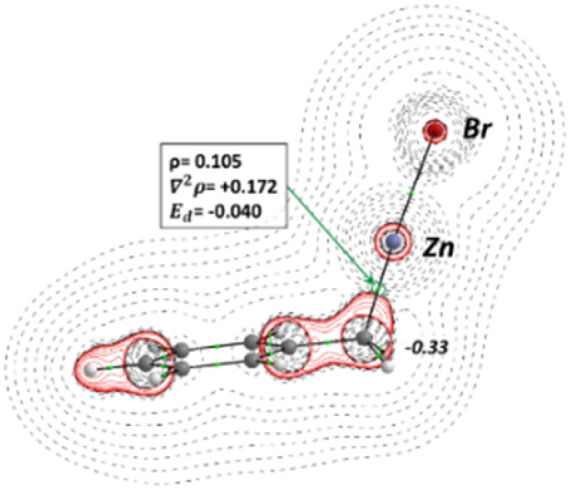
Optimized geometry and bonding scheme and *∇*^2^*ρ*_(*r*)_ contour map for benzylzinc bromide, with the main descriptors of C–Zn BCP, electron density (*ρ*), Laplacian (*∇*^2^*ρ*), and electron energy density (*E*_d_). The integrated QTAIM charge for the corresponding C atom. Black dotted lines represent charge depletion zones (*∇*^2^*ρ*_(*r*)_ > 0) and solid red lines concentration zones (*∇*^2^*ρ*_(*r*)_ < 0). All values are computed at HSEH1PBE/6-311++G(d,p) level.

Fig. 2 depicts the topology of electron density of 1a, and the values for the main descriptors characterizing the Bond Critical Point (BCP) corresponding to the C–Zn bond. The electron density at the bond critical point *ρ*, describes the strength of the chemical bond, and measures the electron population in the area between the connected atoms towards the BCP, quantifying the degree of bond multiplicity. Furthermore, the electron-energy density (*E*_d_), characterizes the stability of the chemical bond, high negative values represent more stabilized interactions, while values close to zero are characteristic to weak atomic interactions. Additionally, the value of Laplacian of electron density (*∇*^2^*ρ*_(*r*)_) for the BCP of C–Zn bond is provided, positive values correspond to charge depletion zones while negative values with charge concentration zones. The contour map of *∇*^2^*ρ*_(*r*)_ indicates a charge depletion zone for the C–Zn BCP (+0.172 e^−^ a_0_^−5^), also the value of *ρ* and *E*_d_, indicates a weak interaction, 0.105 (e^−^a_0_^−3^) and −0.040 (Hartree a_0_^−3^) respectively. Furthermore, from the contour map *∇*^2^*ρ*_(*r*)_ represented in the plane of symmetry, it can be seen that a lobe shape zone corresponding with a negative value of *∇*^2^*ρ*_(*r*)_ (solid red lines) arises from the C atom and is pointed in the direction of Zn, but the area between the C and Zn atoms the charge depletion zone (positive values, black dotted lines) for *∇*^2^*ρ*_(*r*)_ is dominant. Finally, the integrated QTAIM charge for the C atom corresponding to the C–Zn bond is provided, showing a value of −0.33 e^−^, indicating that such carbon atom is characterized by a negative partial charge, this agrees with the lobe shaped area of negative *∇*^2^*ρ*_(*r*)_ that surrounds the C atom. The main conclusion that arises from the QTAIM analysis of 1a is the located negative charge in the C atom and the weakness of the C–Zn bond, which confers a nucleophilic character to the C atom for a further nucleophilic attack.

**Fig. 3:**
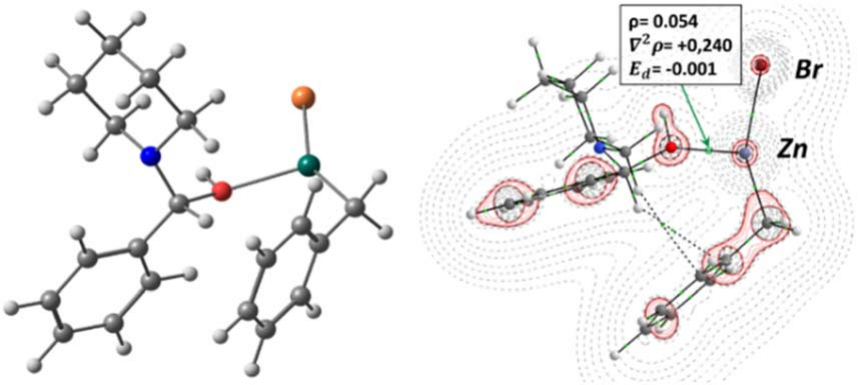
Optimized structure and bonding scheme (right) with *∇*^2^*ρ*_(*r*)_ contour map in the O–Zn–Br plane. Characteristic values for OH–Zn BCP, electron density (*ρ*), Laplacian (*∇*^2^*ρ*), and electron energy density (*E*_d_). Thin dotted lines represent charge depletion zones (*∇*^2^*ρ*_(*r*)_ > 0), while solid lines (red) represent charge concentration zones (*∇*^2^*ρ*_(*r*)_ < 0).

Fig. 3 shows the optimized structure for the intermediate, such structure represents a real minimum of the PES, showing only positive vibrational modes. Furthermore, the bonding scheme and *∇*^2^*ρ*_(*r*)_ plot is represented with the main descriptors for the BCP corresponding to the O–Zn interaction. It can be seen a structure where the OH group is coordinated to the Zn atom with an electrostatic interaction (BCP corresponding to the Zn–O bond is located in a region of charge depletion zone, *∇*^2^*ρ*_(*r*)_ > 0). Furthermore, it shows an arrangement where the aromatic moiety of the benzylzinc structure presents BCPs with the aliphatic group belonging to the amine, this indicates that the dispersion forces of the non-polar groups contribute to the structure stabilization. Thus, 1a can form a stable structure with the OH group of the hemiaminal, facilitating the formation of a more favourable leaving group in subsequent steps.

Previous experimental efforts proposed that 1a deprotonates the hydroxy group to generate toluene and the corresponding metal alkoxide with the subsequent formation of cyclic structures.^24^ In our computational studies, we did several computational attempts to find this proposal, but such deprotonation pathway was not found. Instead, a structure where the OH of the hemiaminal, towards the lone pairs is attached to the Zn atom for both primary and secondary amines was found. The following lines detail the computational study for the Mannich reaction with secondary amines and similar results for our primary amine can be found in the SI.

**Fig. 4:**
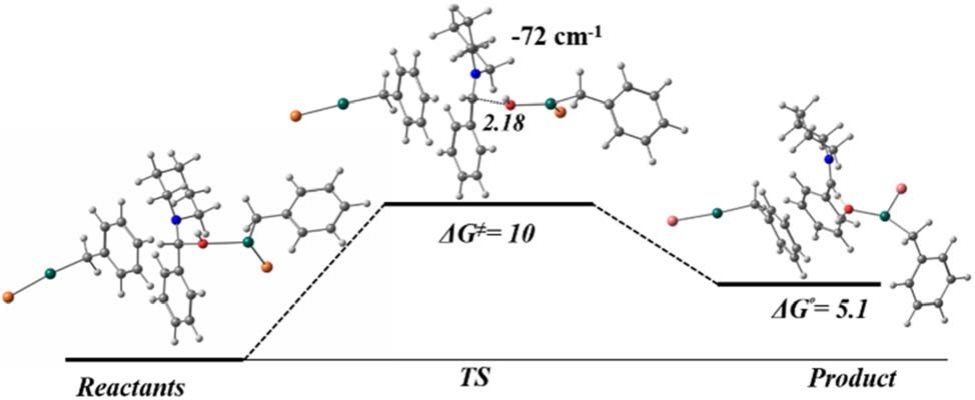
Reaction energy profile for the OH dissociation in Mannich reaction, computed at HSEH1PBE/6-311++G(d,p). Gibbs free energy values in kcal mol^−1^, and distances in Å.

Considering the experimental conditions for the Mannich reaction (various equivalents of 1a were required) the PES has been explored to perform the reaction profile to find a transition state (TS) corresponding to a formation of the C–C bond. Fig. 4 shows the reaction profile where the TS presents an imaginary vibrational mode (−72 cm^−1^) corresponding to the dissociation of the C–O bond. Following a fractional displacement of the imaginary frequency, reactants and products were optimized. In the transition state, the distance between the hydroxy group and the carbon atom of the remaining structure of the amine presents a value of 2.18 Å, while in the hydroxylated scaffolds (belonging to the benzylzinc structure as leaving group) are located in an arrangement that forms a H-bond with the previous structure. Furthermore, the additional equivalent of benzylzinc bromide does not take part in the reaction process, for reactants, TS and products, remains in the surrounding areas due to the interaction between the organic rings with dispersion forces. In consequence a S_N_2 reaction mechanism can be discarded. Concerning Gibbs free energies, the activation energy (Δ*G*^≠^) and the reaction energy (Δ*G*) present 10 kcal mol^−1^ and 5.1 kcal mol^−1^ values, respectively.

**Fig. 5:**
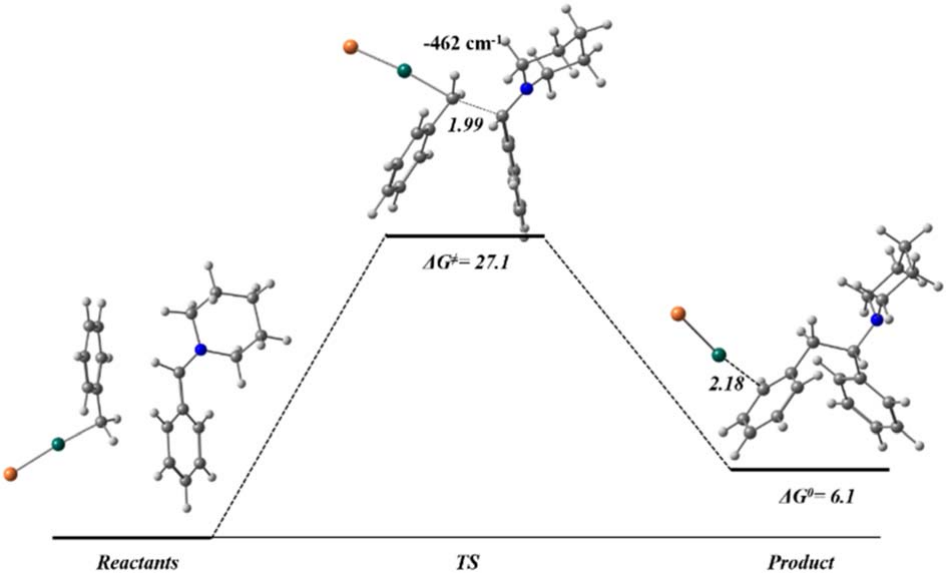
Reaction energy profile for the bond formation in Mannich reaction, with the optimized geometries for reactants, TS and products, computed at HSEH1PBE/6-311++G(d,p). Gibbs free energy values in kcal mol^−1^ and distances in Å.

The following step of the mechanism would be the interaction between the nucleophilic carbon atom of the benzylzinc bromide (QTAIM charge −0.33) and the electrophilic carbon atom of the amine (QTAIM charge of +0.64) (Fig. 5). The transition state presents an imaginary vibrational mode (−462 cm^−1^) corresponding to a bond formation between both carbon atoms with a distance of 1.99 Å. Considering the products, it must be highlighted the arrangement of the Zn atom that is coordinated to the aromatic moiety of the structure. With this data, the reaction shows an exergonic profile with a Δ*G*^≠^ of 27.1 kcal mol^−1^ and a Δ*G* of 6.1 kcal mol^−1^.

**Fig. 6:**
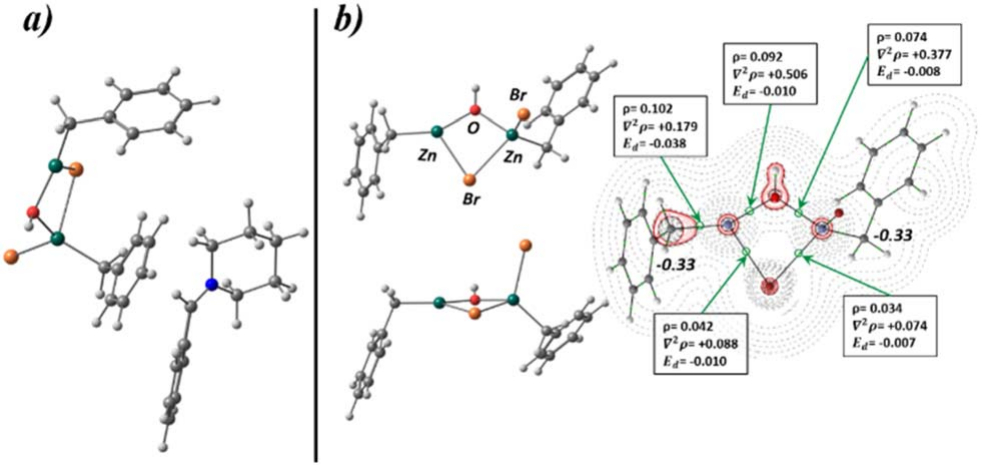
(a) Optimized structures for reactants, two benzylzinc bromide structures coordinated to the OH group and the substituted amine; (b) different orientations for the optimized geometry of (Ph–CH_2_–ZnBr)_2_OH, its bonding scheme and *∇*^2^*ρ*_(*r*)_ contour map in the Br–Zn–OH–Zn ring plane. Relevant BCPs are highlighted with the characteristic values, electron density (*ρ*), Laplacian (*∇*^2^*ρ*), and electron energy density (*E*_d_). Thin dotted lines (black) represent positive values for *∇*^2^*ρ*_(*r*)_, indicating charge depletion zones. Thin solid lines (red) represent negative values for *∇*^2^*ρ*_(*r*)_, charge concentration zones. All values and geometry are computed at HSEH1PBE/6-311++G(d,p) level.

These calculations suggest the reaction proceeds through a S_N_1 reaction mechanism (Fig. 4), as several computational attempts failed to obtain a transition state to support a S_N_2 reaction mechanism. During the optimization process of the calculations, an unexpected result showed that the Br atom interact with the Zn atom of an additional benzylzinc structure, leading the geometry to a local minimum (showing only positive vibrational modes), where the two benzylzinc structures form a cyclic arrangement with the OH and forming the structure (Ph–CH_2_–ZnBr)_2_OH (Fig. 6a). To analyse this new structure and describe its reactivity, this dimeric compound was fully optimized separately (showing no imaginary vibrational frequencies), and the wave function has been analysed within the QTAIM theory (Fig. 6b). Both carbon atoms are linked to Zn atoms, remain with its nucleophilic nature with an integrated QTAIM charge of −0.33 e^−^. The *∇*^2^*ρ*_(*r*)_ reveals that the bonds that conform the cyclic structure present an electrostatic character, where all BCPs are placed in charge depletion zones, *∇*^2^*ρ*_(*r*)_ > 0.

**Fig. 7:**
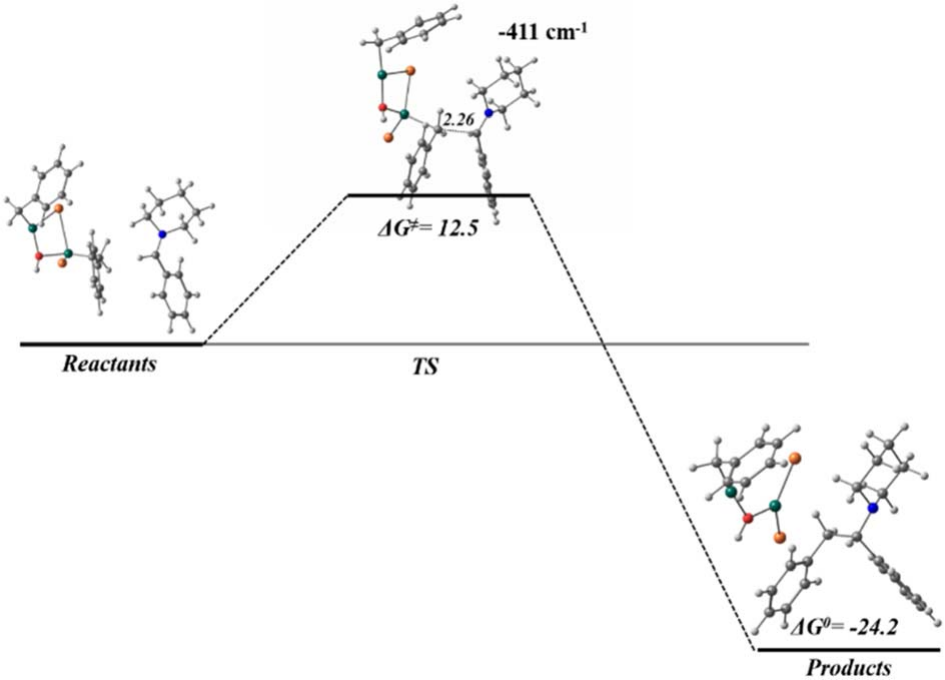
Reaction energy profile for the bond formation in Mannich reaction, with the optimized geometries for reactants, TS and products, computed at HSEH1PBE/6-311++G(d,p). Gibbs free energies in kcal mol^−1^, and distances in Å.

These results suggest that dimeric organozinc species, which are known to form under similar conditions for related systems, constitute a plausible reactive nucleophile in solution and may be responsible for the observed C–C bond formation. The energy profile of the reaction shows the distance between the two carbon atoms involved in the transition state (2.26 Å), with an energy barrier of 12.5 kcal mol^−1^, while products show a stabilization of −24 kcal mol^−1^ (Fig. 7). This energy profile is more favourable than the previous process involving monomeric organometallic species (Fig. 5), with lower activation energy and an exergonic value for products.

**Scheme 2:**
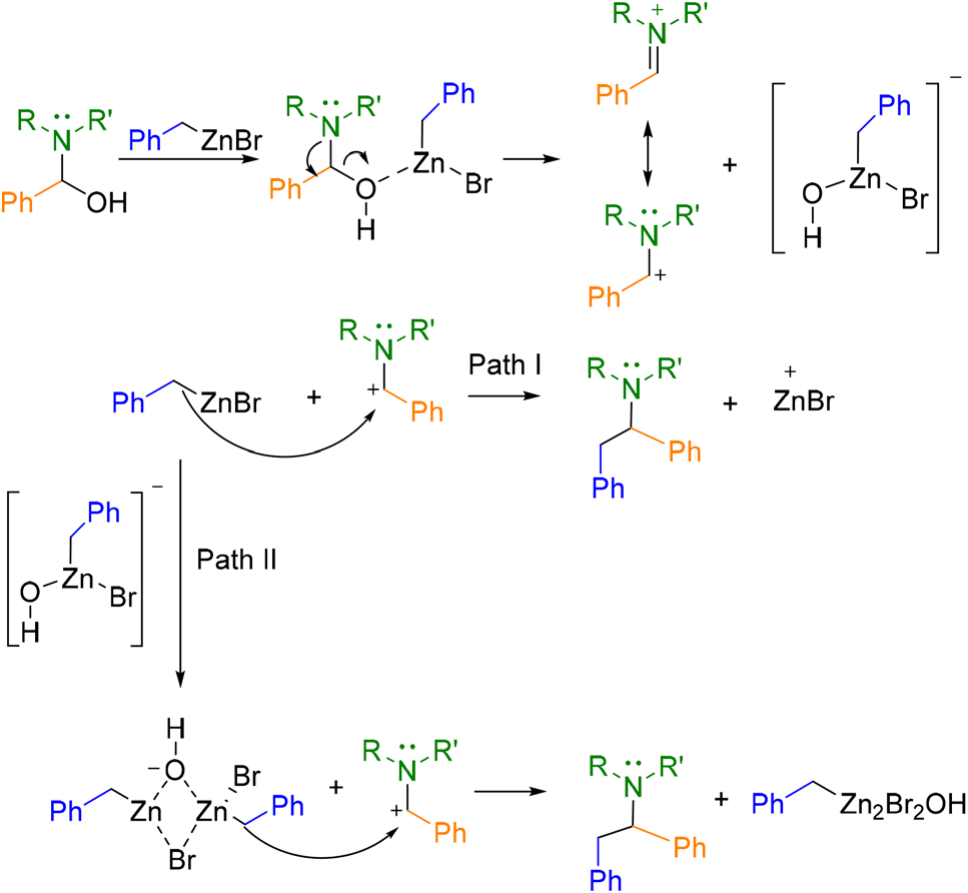
Proposed reaction pathways for Mannich reaction, based on DFT computational studies.

From these calculations, the following two-step mechanism is proposed for this organometallic Mannich reaction (Scheme 2). The first step would involve the interaction of the hydroxy group with one equivalent of the organometallic agent which would promote the formation of an iminium intermediate. Then, two pathways for the nucleophilic attack to the iminium species were proposed. The first one (Path I) would involve the participation of single molecules of benzylzinc bromide as nucleophiles to afford the products. However, a more thermodynamically and kinetically favourable process seems to be the nucleophilic attack of organometallic dimers formed *in situ* in the reaction media (Path II). These DFT findings rationalize the experimentally three-equivalent stoichiometry of the organozinc reagent. Path II requires two equivalents minimum to form reactive organometallic dimers as nucleophiles, while the third equivalent compensates competitive monomer-consuming side-reactions.

## Conclusions

In conclusion, we have developed a fully continuous flow version of a three-component organometallic Mannich reaction, integrating both the *in situ* generation of organozinc agents and the subsequent multicomponent coupling. The transformation proceeds with a residence time of only 5 minutes, delivering densely substituted α-secondary amines in moderate to good yields, minimizing chemical waste, and eliminating the use of zinc powder through the implementation of a microchip reactor. While primary amines successfully participate in the transformation, secondary amines do not afford the desired products under optimized flow conditions and batch versions are still required. Furthermore, DFT studies reveal that organozinc dimers are more likely to react as nucleophiles with the corresponding iminium salts through a S_N_1 reaction pathway.

## Author contributions

L. F. G. for investigation and methodology development, A. S. G. for computational studies, L. F. P. for writing and editing, E. L. for conceptualization, project administration, supervision, writing and editing.

## Conflicts of interest

There are no conflicts to declare.

## Supplementary Material

RA-016-D6RA01038E-s001

## Data Availability

The data that support the findings of this study are available from the corresponding author upon reasonable request. Supplementary information (SI): experimental procedures, flow setup details, optimization tables, characterization data, and computational details. See DOI: https://doi.org/10.1039/d6ra01038e.
