# Supplementary material for: Mannich reaction with organozinc reagents in continuous flow: experimental and computational studies
Source: RSC Adv. 2026 Mar 16;16(16):14251–8. doi: 10.1039/d6ra01038e (PMC12990320; doi:10.1039/d6ra01038e)
Supplement: RA-016-D6RA01038E-s001 [file RA-016-D6RA01038E-s001.pdf]

Supplementary Information (SI) for RSC Advances.
This journal is © The Royal Society of Chemistry 2026

Supporting information

Mannich Reaction with Organozinc Reagents in Continuous Flow: Experimental and Computational Studies

Lucas Fraile-González,[a] Ángel Sánchez-González,[a,b] Laura F. Peña,[c] and Enol López*[a]

Table of Contents

General information 3

General procedure A for the synthesis of organozinc derivatives 1 3

Characterization of organozinc derivatives 3

General procedure B for the synthesis of products 4 in flow 4

General procedure C for the synthesis of products 4h, 4k, 4p and 5 in batch 5

Characterization of compounds 4 and 5 5

Computational studies 45

General information

Unless otherwise noted, experiments were carried out with dry solvents under nitrogen atmosphere. Tetrahydrofuran (THF) and acetonitrile (CH_3CN) were dried with preactivated molecular sieves. NMR spectra were recorded at nuclear magnetic resonance service of the Laboratory of Instrumental Techniques (L.T.I., www.laboratoriotecnicasinstrumentales.es) University of Valladolid at Varian 400 MHz (^{1}H, 399.85 MHz; ^{13}C, 100.61 MHz) spectrometer at room temperature (25 °C). Chemical shifts (δ) were reported in parts per million (ppm) relative to the residual solvent peaks recorded, rounded to the nearest 0.01 for ^{1}H-NMR and 0.1 for ^{13}C-NMR (reference: $CDCl_3$ [^{1}H: 7.26, ^{13}C: 77.0]). Spin-spin coupling constants (*J*) in ^{1}H-NMR were given in Hz to the nearest 0.1 Hz, and peak multiplicity was indicated as follows s (singlet), d (doublet), t (triplet), q (quartet), m (multiplet) and bs (broad signal). ^{13}C NMR were recorded with complete proton decoupling. Carbon types, structure assignments and attribution of peaks were determined from two-dimensional correlation experiments (HSQC, COSY and HMBC). High resolution mass spectra (HRMS) were measured at mass spectrometry service of the Laboratory of Instrumental Techniques, University of Valladolid, on a UPLC-MS system (UPLC: Waters ACQUITY H-class UPLC; MS: Bruker Maxis Impact) by electrospray ionization (ESI positive).

General procedure A for the synthesis of organozinc derivatives **1**

A solution of 1.0 M trimethylchlorosilane (TMSCl) and 0.24 M 1-bromo-2-chloroethane was prepared under nitrogen (N_2) atmosphere in a dried flask by dissolving 1.25 mL of TMSCl and 0.2 mL of 1-bromo-2-chloroethane in 10 mL of dried tetrahydrofuran (THF). 5 mL of this solution were passed through the 10 mm internal diameter Omni-fit column containing Zn (12 g) at rt and 0.5 mL/min flow rate.

Then, a solution of the corresponding bromo derivative 0.5 M in THF was passed through the column containing activated Zn at rt and flow rate of 0.5 mL/min. The outcoming solution of the organozinc compound was collected in a closed flask under nitrogen (N_2) atmosphere.

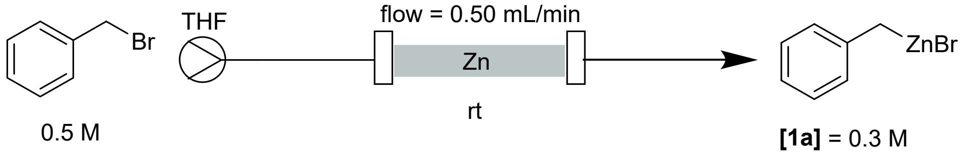

Scheme S1. Synthesis of organozinc derivatives in continuous flow.

Titration with I_2 was used to determine approximatively the concentration of the organozinc solution obtained. A known quantity (25-30 mg approx.) of I_2 was weighted and dissolved in 0.5 mL of dry THF in a closed vial under N_2 atmosphere. The solution of the organozinc compound was added dropwise until the I_2 solution became transparent. The volume of organozinc used to titrate the I_2 was used to calculate the molarity (Equation S1).

$$[RZnBr](M) = \frac{W\ iodine\ (mg)}{MW\ iodine\ \left(\frac{mg}{mol}\right) * V\ RZnBr\ (mL)}$$

Equation S1. Calculation of the molarity of the organozinc compound (W = weight; MW = molecular weight; V = volume).

Characterization of organozinc derivatives

Benzyl zinc (II) bromide (**1a**)

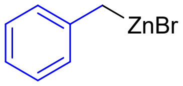

Prepared following General procedure A; **Titration**: 0.30 M.

(2-methoxy-2-oxoethyl)zinc(II) bromide (**1b**)

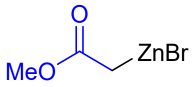

Prepared following General procedure A; **Titration**: 0.40 M

General procedure B for the synthesis of products **4** in flow

A solution of the aldehyde **3** (0.22 mmol, 1.1 eq.), and the amine **2** (0.20 mmol, 1.0 eq.), was prepared in CH_3CN (2 mL) and stirred for 20 minutes. Then, the solution of **2** + **3** in CH_3CN and a solution of **1** (3 eq., prepared according to General Procedure A) were pumped separately into two lines through a 1-mL chip microreactor chip (channel size 1mm, dimensions 115x60x6mm) at flow rate= 0.20 mL/min and at 30 ºC (Scheme S2).

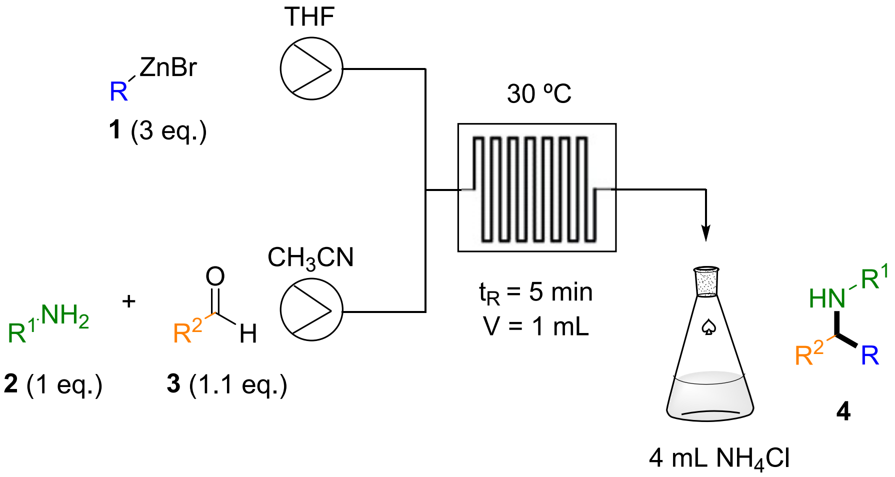

Scheme S2. Mannich reaction in flow.

The reaction crude was quenched with 4 mL of saturated NH_4Cl solution. The resulting mixture was extracted with ethyl acetate (10 mL) and washed with water (2x10 mL). The organic layer was dried over anhydrous Na_2SO_4, and the drying agent was filtered. The solvent was concentrated under reduced pressure, and the crude was purified using solid-supported cation exchange cartridges (SCX) (see Figure S1). The SCX matrix was activated with 2 mL of CH_3OH and 2 mL of CH_2Cl_2. The crude was diluted in the minimum volume of CH_2Cl_2 and inserted into the cartridge. Then, the cartridge was washed with 2 mL of CH_2Cl_2, and the purified product was eluted using 2 mL of a 7 N NH_3 in CH_3OH. Solvents were removed under reduced pressure, yielding the corresponding isolated products.

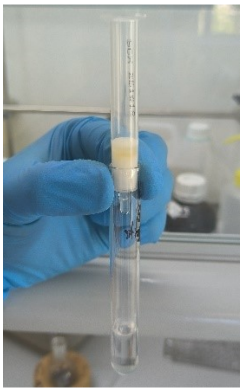

Figure S1. SCX cartridge used in work-up.

General procedure C for the synthesis of products **4h**, **4k**, **4p** and **5** in batch

For the synthesis of these products, the solution of the corresponding aldehyde and the amine in CH_3CN exhibited insufficient solubility to carry out the reaction in continuous flow. The following batch methodology was employed to circumvent this issue.

A solution of the organozinc reagent **1a** (3 eq., 2 mL prepared following General procedure A) was added to a 25-mL flask containing aldehyde **3** (0.22 mmol, 1.1 eq.), and amine **2** (0.20 mmol, 1.0 eq.), in 2 mL of CH_3CN and the reaction was stirred for 24 hours. The reaction was quenched with 4 mL of saturated NH_4Cl solution. The resulting mixture was extracted with ethyl acetate (10 mL) and washed with water (2x10 mL). The organic layer was dried over anhydrous Na_2SO_4, and the drying agent was filtered. The solvent was concentrated under reduced pressure, and the crude was purified using solid-supported cation exchange cartridges (SCX). First, the SCX matrix was activated with 2 mL of CH_3OH and 2 mL of CH_2Cl_2. The crude was diluted in the minimum volume of CH_2Cl_2 and applied directly to the activated matrix. Then, the cartridge was washed with 2 mL of CH_2Cl_2, and the purified product was eluted using 2 mL of a 7 N NH_3 in CH_3OH. Solvents were removed under reduced pressure, yielding the isolated product.

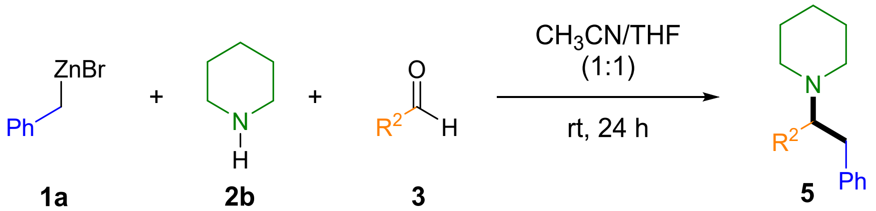

Scheme S3. Mannich reaction in batch.

Characterization of compounds **4** and **5**

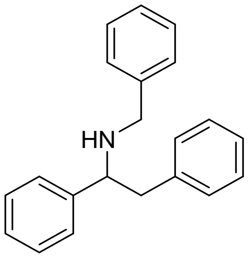

N-benzyl-1,2-diphenylethanamine (**4a**) was obtained following General procedure B.

^{1}H-NMR (400 MHz, $CDCl_3$): δ (ppm) 7.39 – 7.34 (m, 4H), 7.31 – 7.20 (m, 7H), 7.15 – 7.10 (m, 4H), 3.92 (dd, *J* = 8.1, 6.1 Hz, 1H), 3.70 (d, *J* = 13.5 Hz, 1H), 3.50 (d, *J* = 13.5 Hz, 1H), 3.04 – 2.91 (m, 2H), 2.60 (bs, 1H).

^{13}C-NMR (101 MHz, $CDCl_3$): δ (ppm) 143.3, 140.0, 138.7, 129.3, 2x 128.4, 128.3, 128.0, 127.5, 127.2, 126.8, 126.4, 63.6, 51.2, 45.1.

HRMS (ESI-TOF): mass calculated for $C_{21}H_{21}N$, 287.1674; m/z found, 288.1744 $[M+H]^+$.

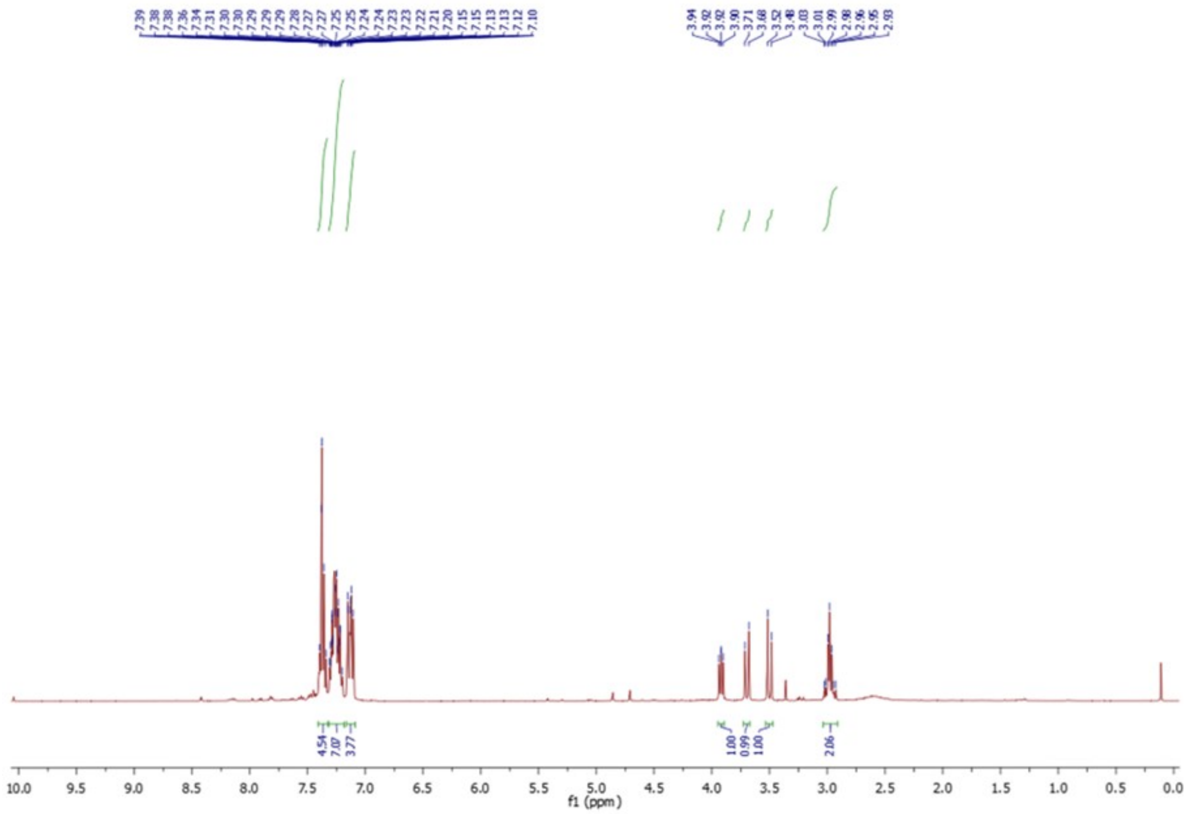

7.39 7.38 7.38 7.36 7.34 7.31 7.30 7.30 7.29 7.29 7.29 7.28 7.27 7.27 7.25 7.25 7.24 7.24 7.23 7.23 7.23 7.22 7.21 7.20 7.15 7.15 7.13 7.13 7.12 7.10
3.94 3.92 3.92 3.90 3.71 3.68 3.52 3.48 3.03 3.01 2.99 2.98 2.96 2.95 2.93
4.54 7.07 3.77
1.00 0.99 1.00
2.06
f1 (ppm)

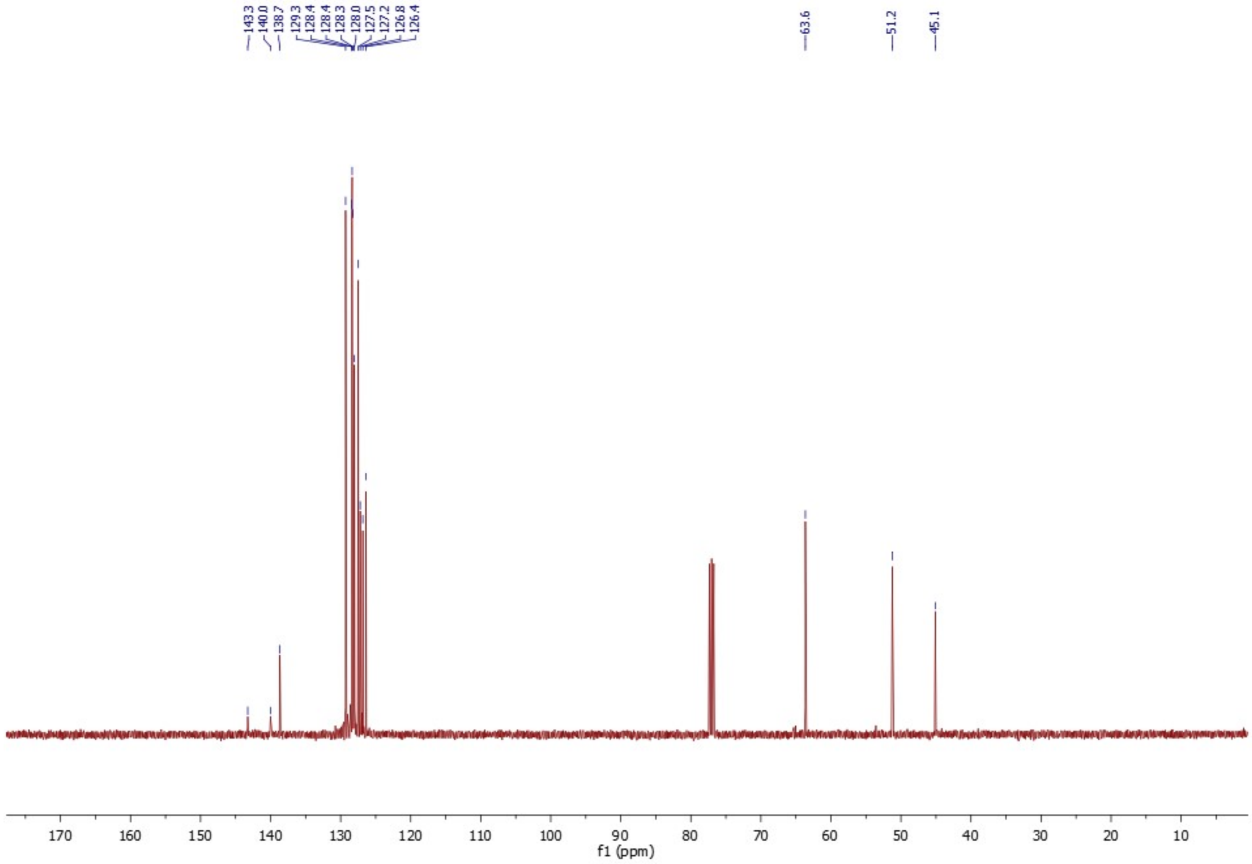

143.3 140.0 138.7 129.3 128.4 128.4 128.3 128.0 127.5 127.2 126.8 126.4
63.6
51.2
45.1
f1 (ppm)

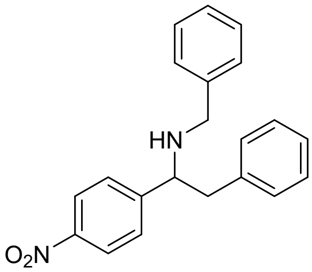

N-benzyl-1-(4-nitrophenyl)-2-phenylethanamide (**4b**) was obtained following General procedure B.

^{1}H-NMR (400 MHz, CDCl$_3$): δ (ppm) 8.19 (d, *J* = 8.8 Hz, 2H), 7.52 (d, *J* = 8.8 Hz, 2H), 7.30 – 7.21 (m, 6H), 7.11 – 7.05 (m, 4H), 4.02 (t, *J* = 7.1 Hz, 1H), 3.66 (d, *J* = 13.6 Hz, 1H), 3.48 (d, *J* = 13.6 Hz, 1H), 3.20 (bs, 1H), 2.93 (d, *J* = 7.1 Hz, 2H).

^{13}C-NMR (101 MHz, CDCl$_3$): δ (ppm) 151.3, 147.2, 139.5, 137.5, 129.9, 129.2, 128.6, 128.4, 128.3, 127.9, 127.1, 126.8, 123.7, 63.2, 51.4, 44.9.

HRMS (ESI-TOF): mass calculated for $C_{21}H_{20}N_2O_2$, 332.1525; m/z found, 333.1601 $[M+H]^+$.

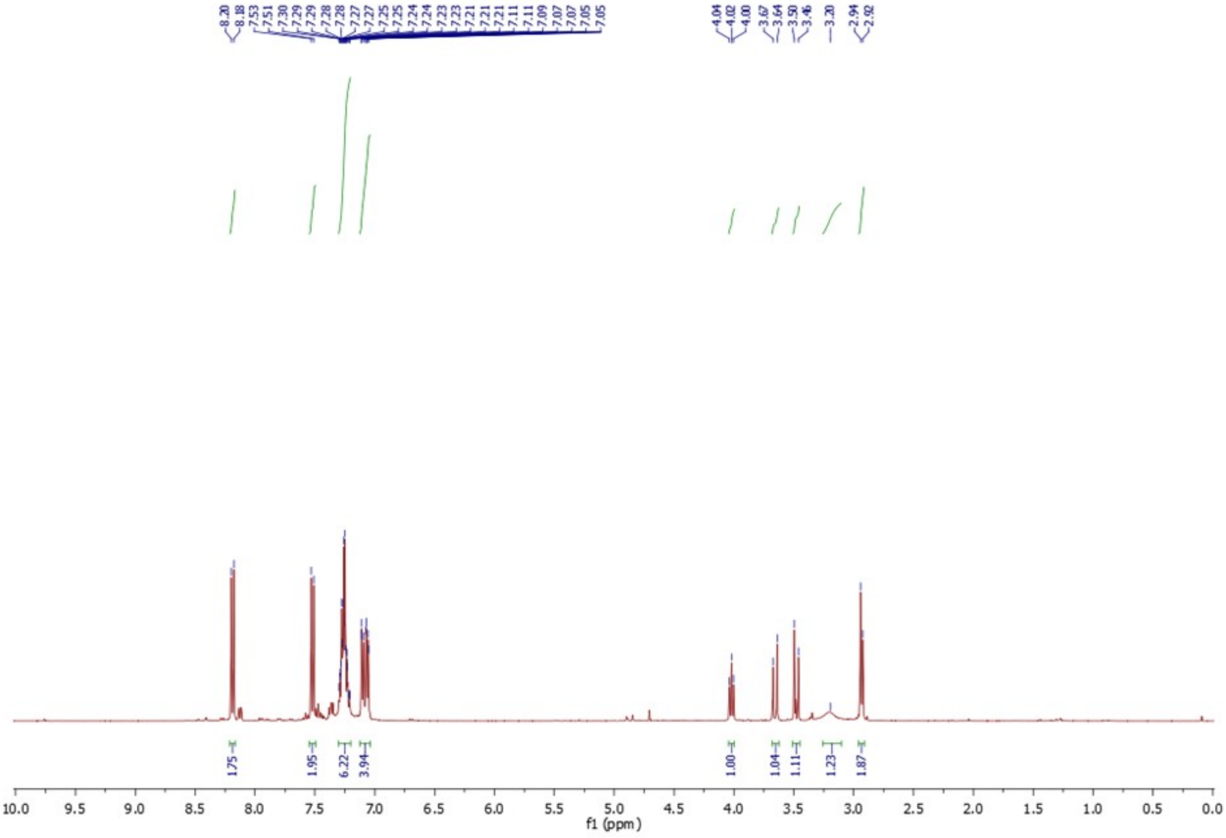

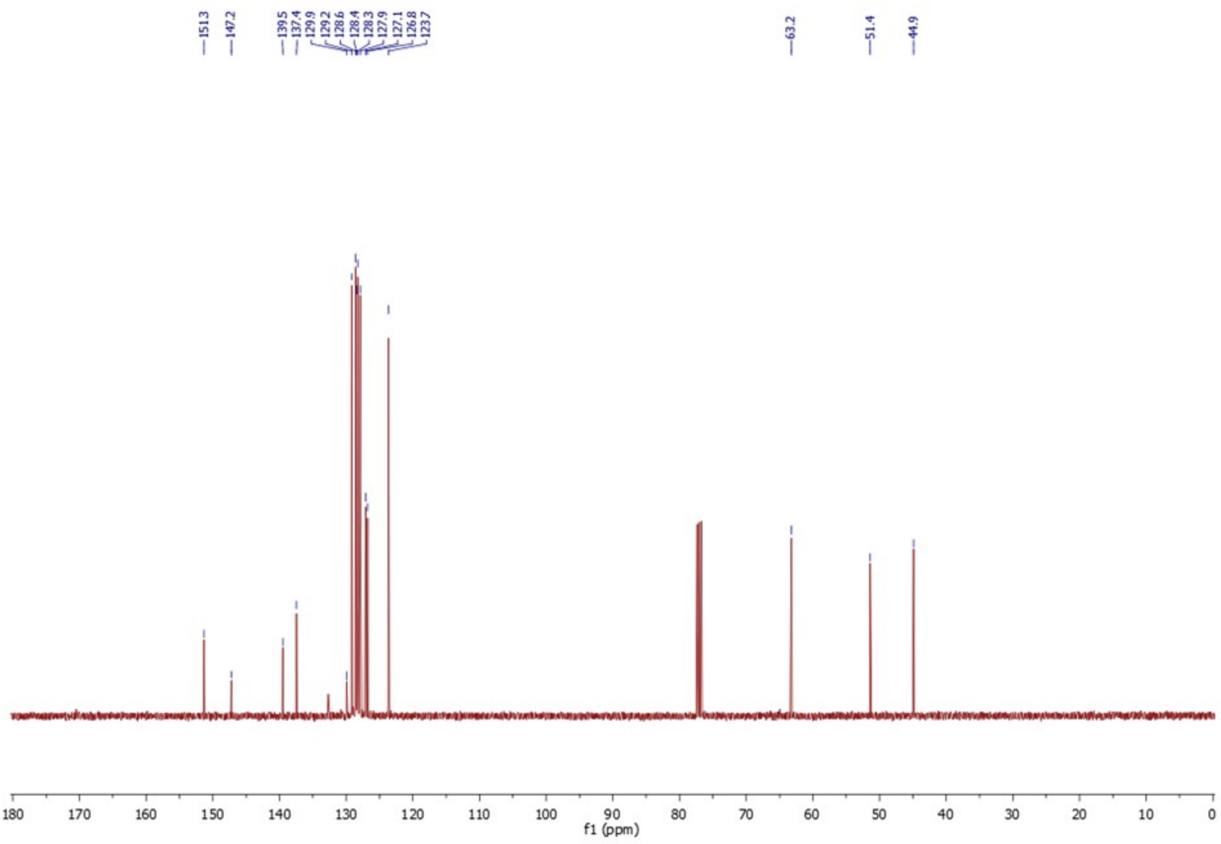

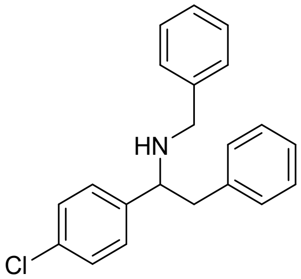

N-benzyl-1-(4-chlorophenyl)-2-phenylethanamide (**4c**) was obtained following General procedure B.

^{1}H-NMR (400 MHz, CDCl$_3$): δ (ppm) 7.32 – 7.20 (m, 10H), 7.12 – 7.06 (m, 4H), 3.87 (t, *J* = 7.0 Hz, 1H), 3.66 (d, *J* = 13.5 Hz, 1H), 3.46 (d, *J* = 13.5 Hz, 1H), 2.92 (d, *J* = 7.0 Hz, 2H).

^{13}C-NMR (101 MHz, CDCl$_3$): δ (ppm) 138.2, 129.2, 128.8, 128.5, 128.4, 128.3, 128.0, 126.9, 126.5, 63.0, 51.2, 45.0.

HRMS (ESI-TOF): mass calculated for $C_{21}H_{20}NCl$, 321.1284; m/z found, 322.1365 $[M+H]^+$.

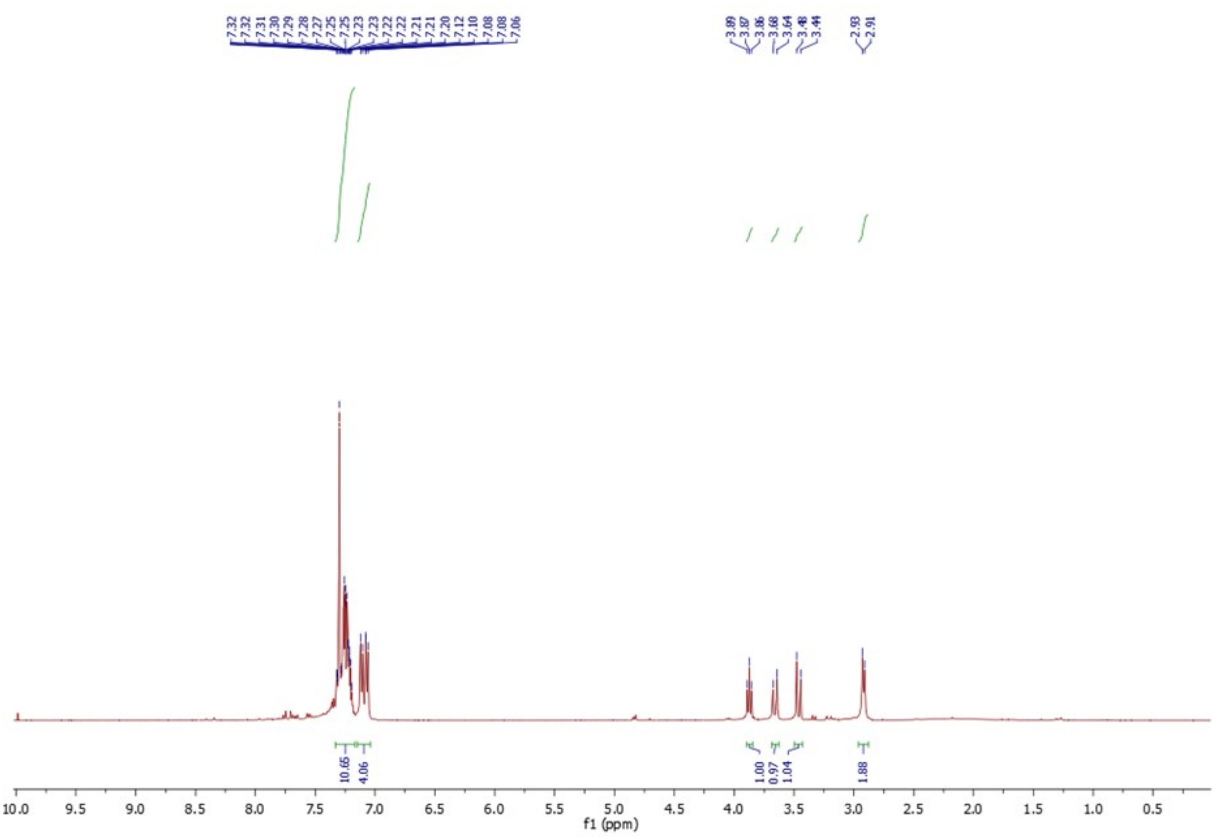
7.32 7.32 7.31 7.30 7.29 7.28 7.27 7.25 7.25 7.23 7.23 7.22 7.22 7.21 7.21 7.20 7.12 7.10 7.08 7.06
3.89 3.87 3.86 3.68 3.64 3.48 3.44
2.93 2.91
10.65 4.06 1.00 0.97 1.04 1.88
f1 (ppm)

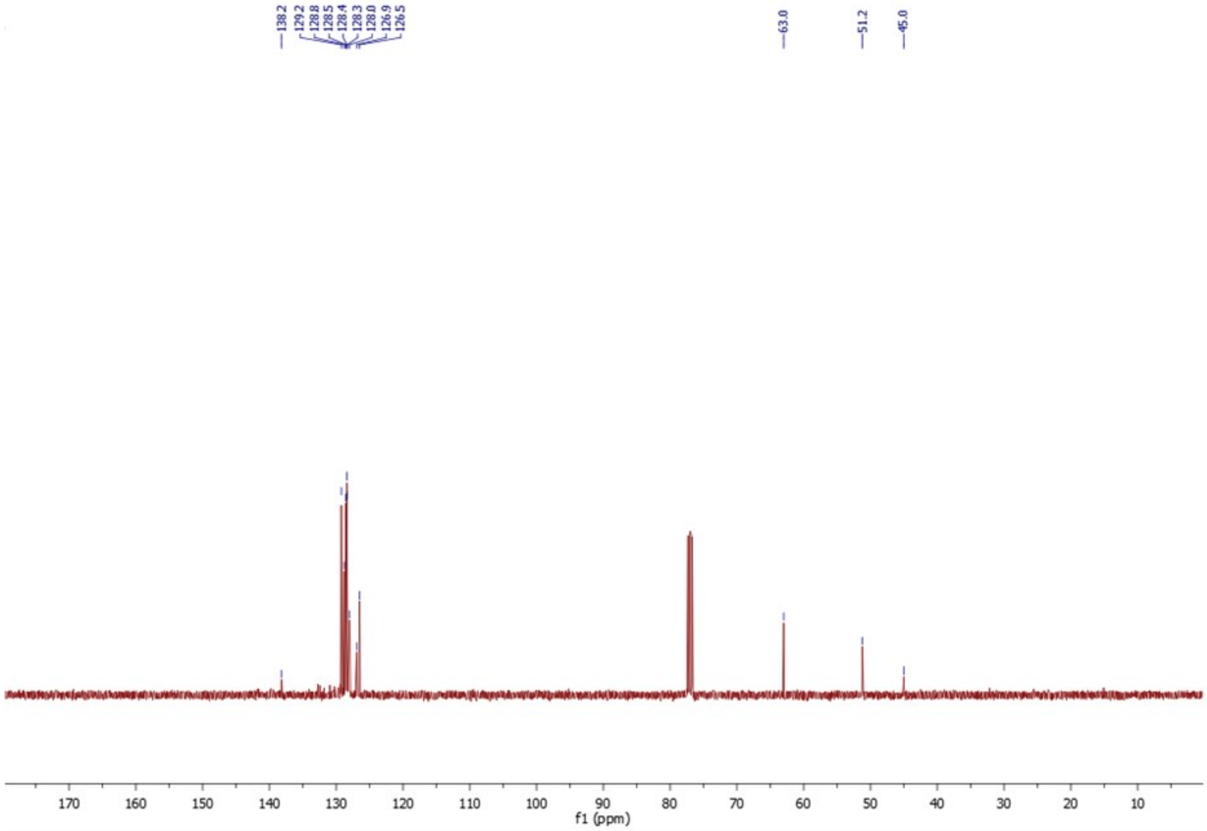
138.2 129.2 128.8 128.5 128.4 128.3 128.0 126.9 126.5
63.0 51.2 45.0
f1 (ppm)

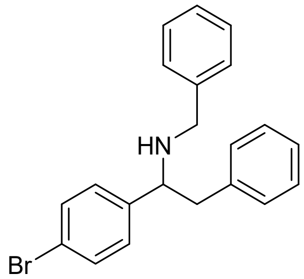

N-benzyl-1-(4-bromophenyl)-2-phenylethanamide (**4d**) was obtained following General procedure B.

^{1}H-NMR (400 MHz, CDCl$_3$): δ (ppm) 7.46 (d, *J* = 8.4 Hz, 2H), 7.28 – 7.18 (m, 8H), 7.12 – 7.05 (m, 4H), 3.87 (t, *J* = 7.1 Hz, 1H), 3.67 (d, *J* = 13.5 Hz, 1H), 3.47 (d, *J* = 13.5 Hz, 1H), 3.22 (bs, 1H), 2.93 (d, *J* = 7.1 Hz, 2H).

^{13}C-NMR (101 MHz, CDCl$_3$): δ (ppm) 141.9, 139.3, 138.0, 131.5, 129.2, 2x 128.4, 128.1, 121.0, 63.0, 51.1, 44.8.

HRMS (ESI-TOF): mass calculated for $C_{21}H_{20}NBr$, 365.0779; m/z found, 366.0858 $[M+H]^+$.

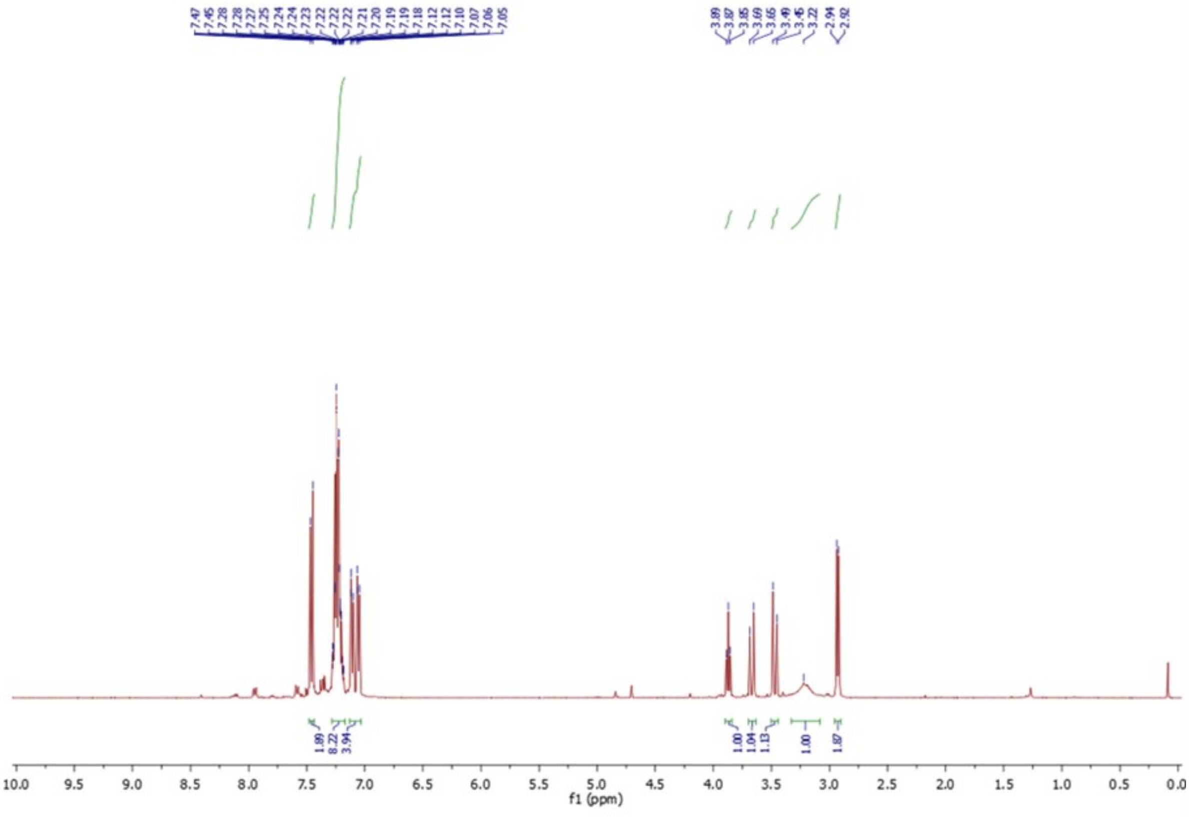

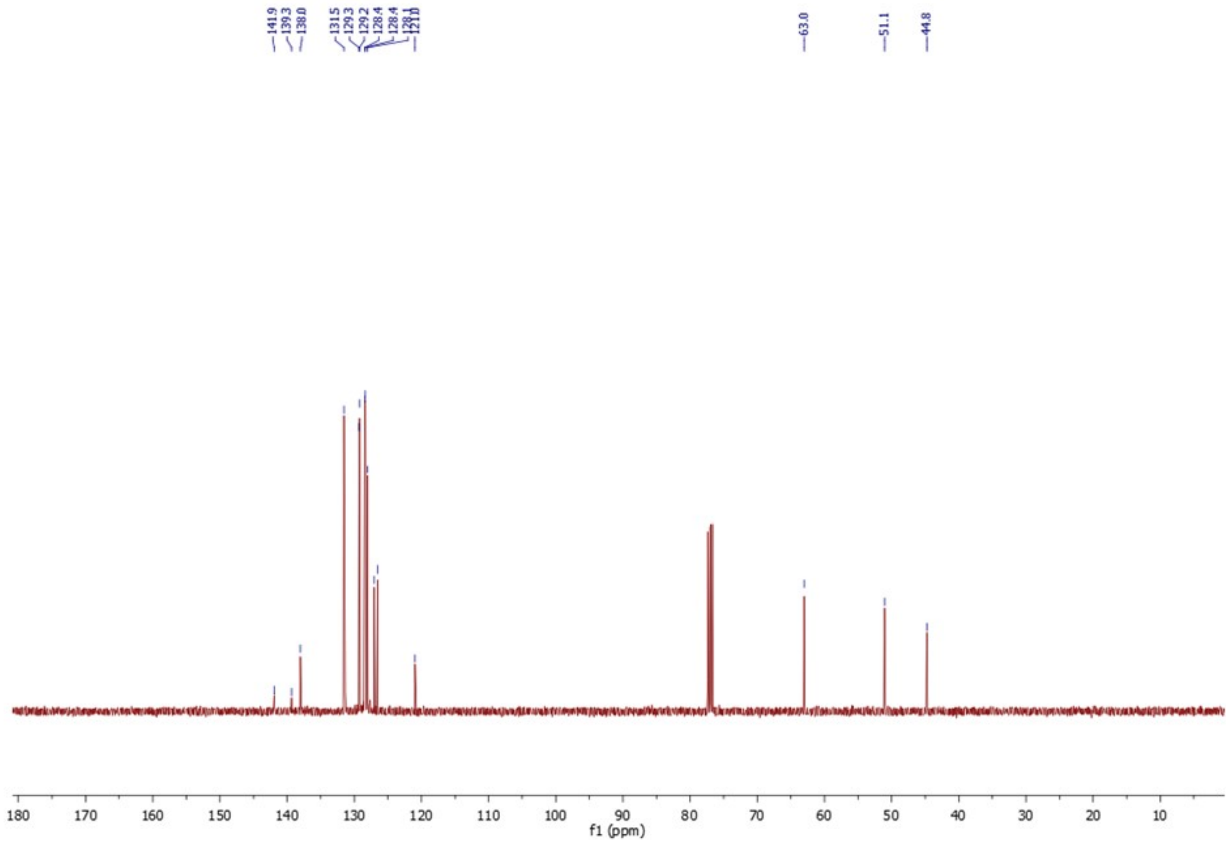

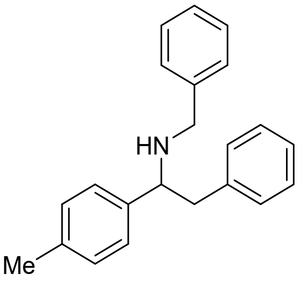

N-benzyl-2-phenyl-1-(*p*-tolyl)ethanamine (**4e**) was obtained following General procedure B.

^{1}H-NMR (400 MHz, $CDCl_3$): δ (ppm) 7.39 – 7.13 (m, 14H), 3.90 (dd, *J* = 8.5, 5.5 Hz, 1H), 3.70 (d, *J* = 13.5 Hz, 1H), 3.50 (d, *J* = 13.5 Hz, 1H), 3.00 (dd, *J* = 13.6, 5.5 Hz, 2H), 2.93 (dd, *J* = 13.6, 8.5 Hz, 2H), 2.40 (s, 3H), 1.89 (s, 1H).

^{13}C-NMR (101 MHz, $CDCl_3$): δ (ppm) 140.6, 140.5, 138.9, 136.7, 129.3, 129.1, 128.4, 128.3, 128.0, 127.3, 126.7, 126.3, 63.3, 51.3, 45.3, 21.2.

HRMS (ESI-TOF): mass calculated for $C_{22}H_{23}N$, 301.1830; m/z found, 302.1912 $[M+H]^+$.

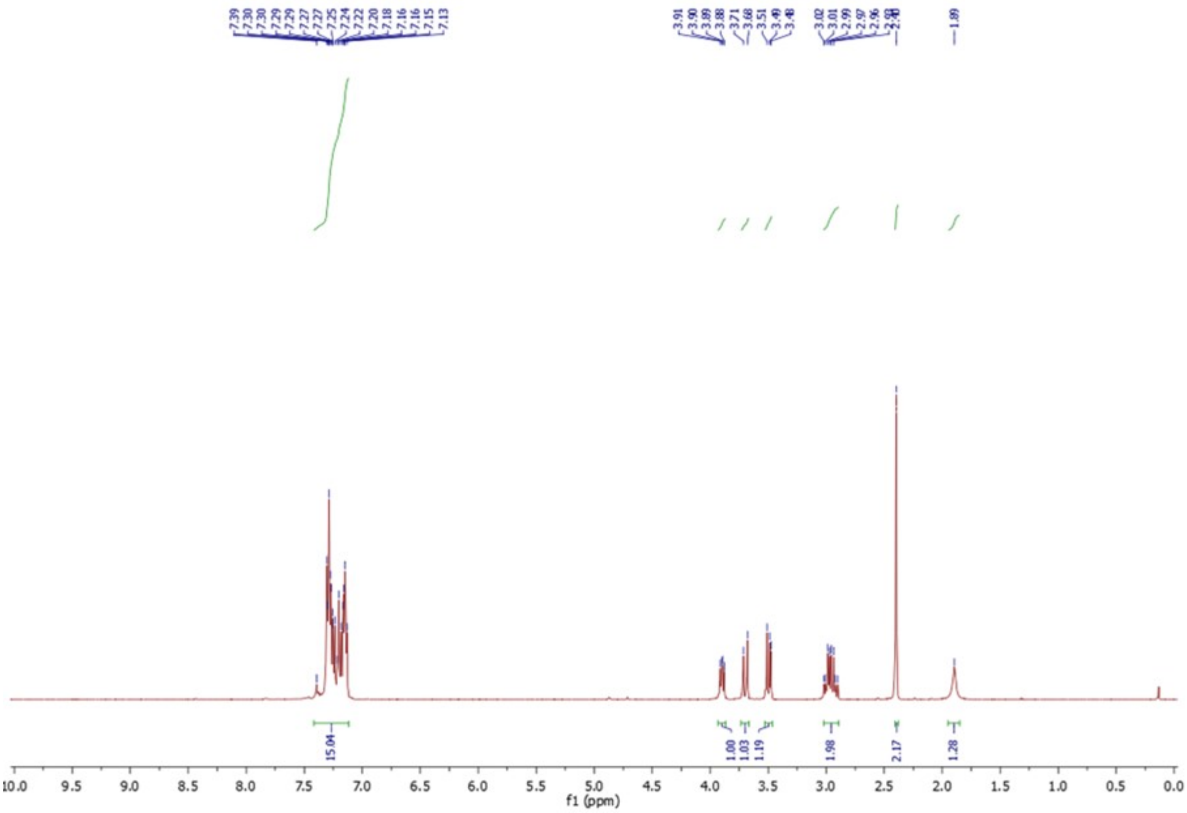
7.39
7.30
7.30
7.29
7.29
7.27
7.25
7.24
7.22
7.20
7.18
7.16
7.15
7.13
3.91
3.90
3.89
3.88
3.71
3.68
3.51
3.49
3.48
3.02
3.01
2.99
2.97
2.96
1.89
15.04
1.00
1.03
1.19
1.98
2.17
1.28
10.0 9.5 9.0 8.5 8.0 7.5 7.0 6.5 6.0 5.5 5.0 4.5 4.0 3.5 3.0 2.5 2.0 1.5 1.0 0.5 0.0
f1 (ppm)

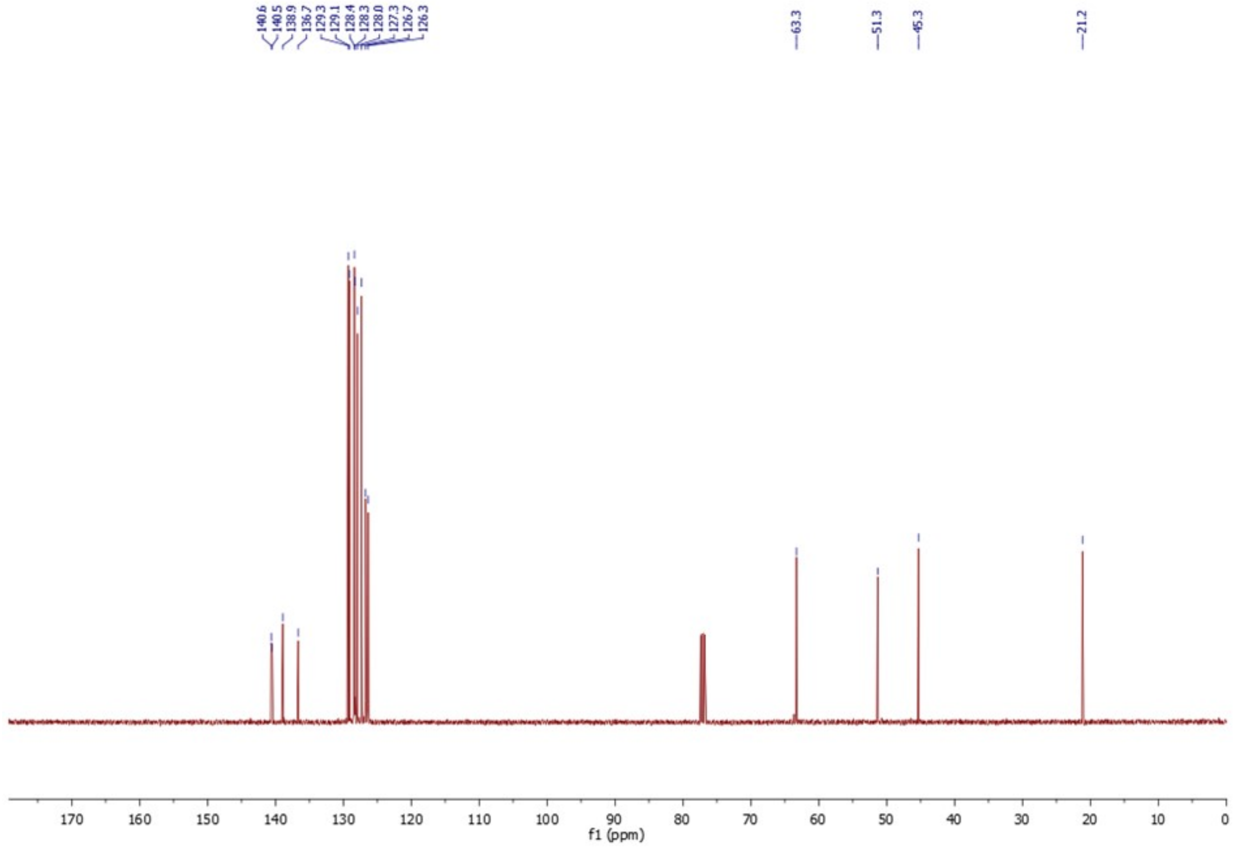
140.6
140.5
138.9
136.7
129.3
129.1
128.4
128.3
128.0
127.3
126.7
126.3
63.3
51.3
45.3
21.2
170 160 150 140 130 120 110 100 90 80 70 60 50 40 30 20 10 0
f1 (ppm)

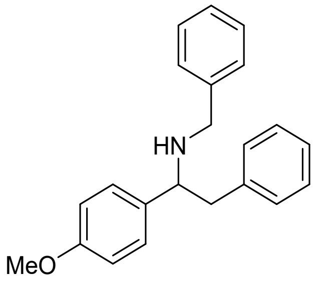

N-benzyl-1-(4-methoxyphenyl)-2-phenylethanamide (**4f**) was obtained following General procedure B.

^{1}H-NMR (400 MHz, $CDCl_3$): δ (ppm) 7.35 – 7.02 (m, 12H), 6.88 (d, *J* = 8.3 Hz, 2H) 3.92 – 3.81 (m, 1H), 3.81 (s, 3H), 3.71 (d, *J* = 12.7 Hz, 1H), 3.49 (d, *J* = 12.7 Hz, 1H), 2.98 (d, *J* = 6.0 Hz, 2H).

^{13}C-NMR (101 MHz, $CDCl_3$): δ (ppm) 158.9, 138.4, 2x 129.3, 128.4,2x 128.3, 128.1, 127.5, 127.1, 126.3, 113.8, 63.0, 55.2, 50.9.

HRMS (ESI-TOF): mass calculated for $C_{22}H_{23}NO$, 317.1780; m/z found, 318.1856 $[M+H]^+$.

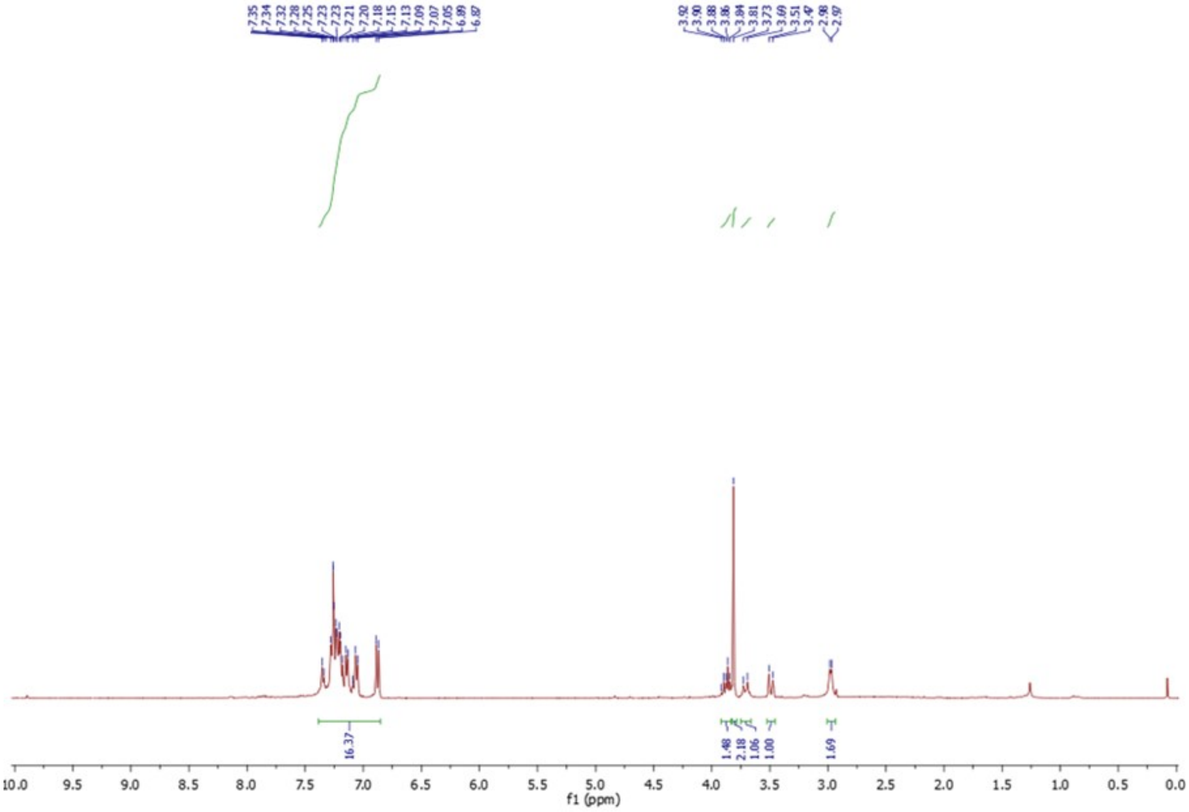

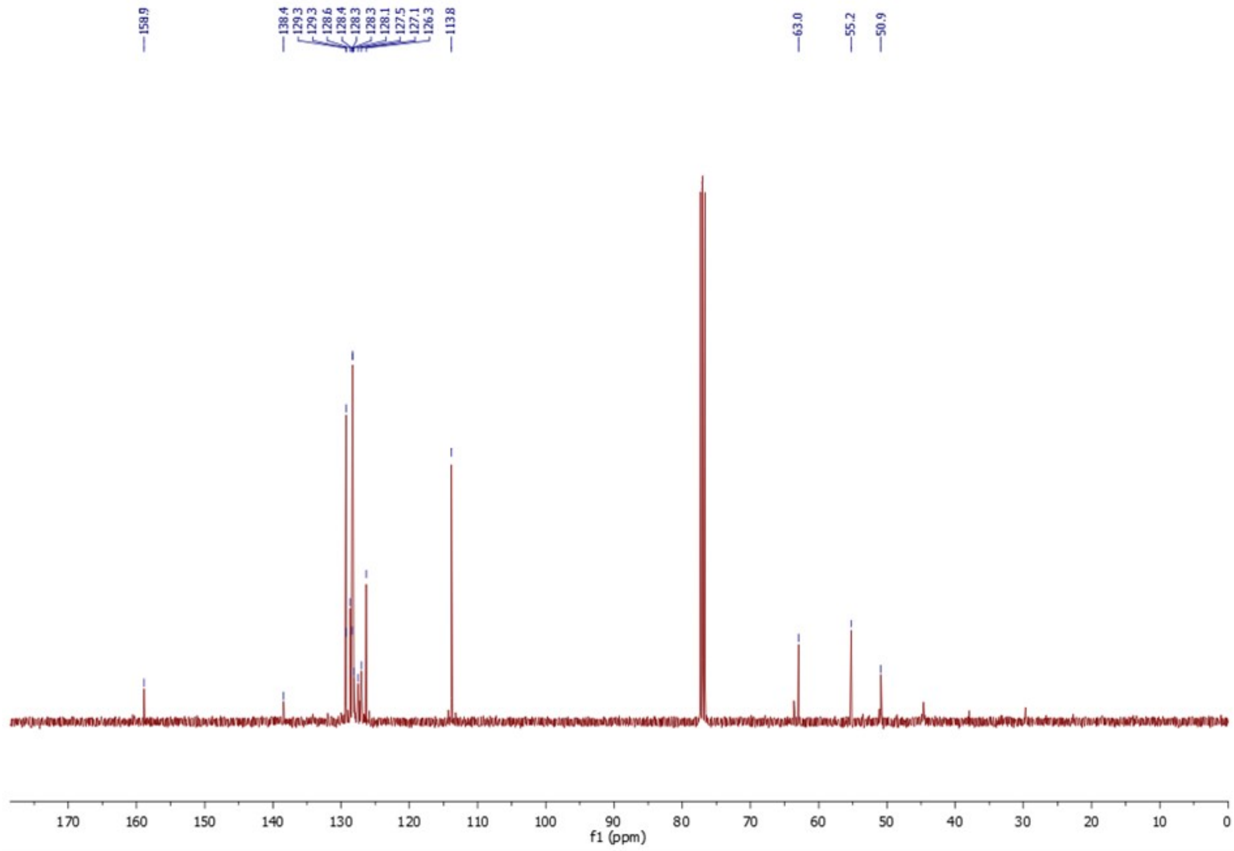

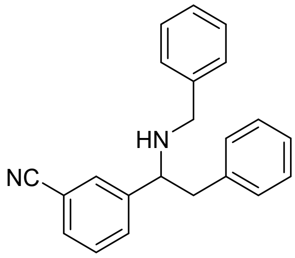

3-(1-(benzylamino)-2-phenylethyl)benzonitrile (**4g**) was obtained following the General procedure B.

^{1}H-NMR (400 MHz, CDCl$_3$): δ (ppm) 7.68 (t, *J* = 1.5 Hz, 1H), 7.59 (dt, *J* = 7.8, 1.5 Hz, 1H), 7.56 (dt, *J* = 7.8, 1.5 Hz, 1H), 7.42 (t, *J* = 7.8 Hz, 1H), 7.30 – 7.21 (m, 6H), 7.11 – 7.06 (m, 4H), 3.93 (dd, *J* = 8.0, 6.2 Hz, 1H), 3.64 (d, *J* = 13.5 Hz, 1H), 3.46 (d, *J* = 13.5, 1H), 2.95 – 2.86 (m, 2H), 2.05 (bs, 1H).

^{13}C-NMR (101 MHz, CDCl$_3$): δ (ppm) 145.4, 139.8, 137.7, 132.0, 2x 129.2, 128.6, 128.4, 127.8, 127.0, 119.0, 112.4, 63.1, 51.4, 45.2.

HRMS (ESI-TOF): mass calculated for $C_{22}H_{20}N_2$, 312.1626; m/z found, 313.1700 [M+H]$^+$.

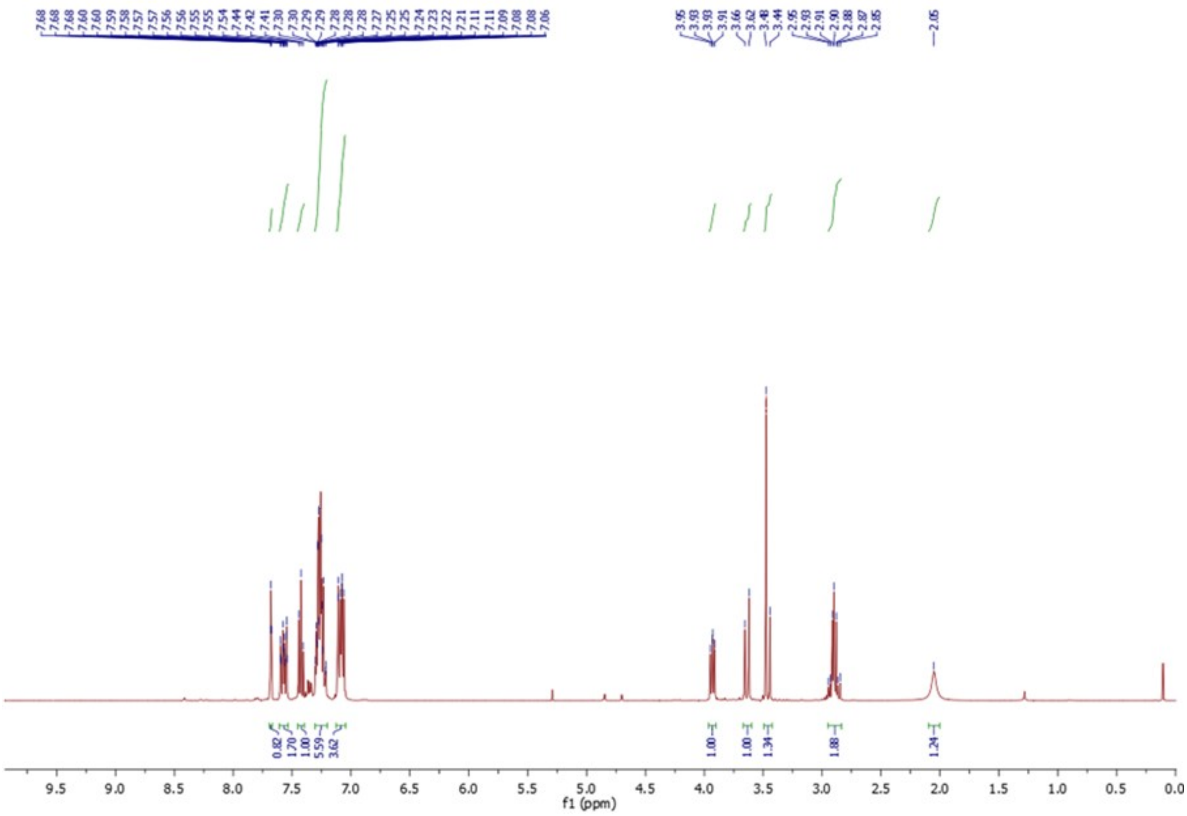
7.68 7.68 7.68 7.60 7.60 7.59 7.58 7.57 7.56 7.56 7.55 7.55 7.54 7.44 7.42 7.41 7.30 7.30 7.29 7.29 7.28 7.28 7.28 7.27 7.25 7.25 7.24 7.23 7.22 7.21 7.11 7.09 7.08 7.08 7.06
3.95 3.93 3.93 3.91 3.66 3.62 3.48 3.44 2.95 2.93 2.91 2.90 2.88 2.87 2.85
2.05
0.82 1.70 1.00 5.59 3.62
1.00 1.00 1.34 1.88 1.24
9.5 9.0 8.5 8.0 7.5 7.0 6.5 6.0 5.5 5.0 4.5 4.0 3.5 3.0 2.5 2.0 1.5 1.0 0.5 0.0
f1 (ppm)

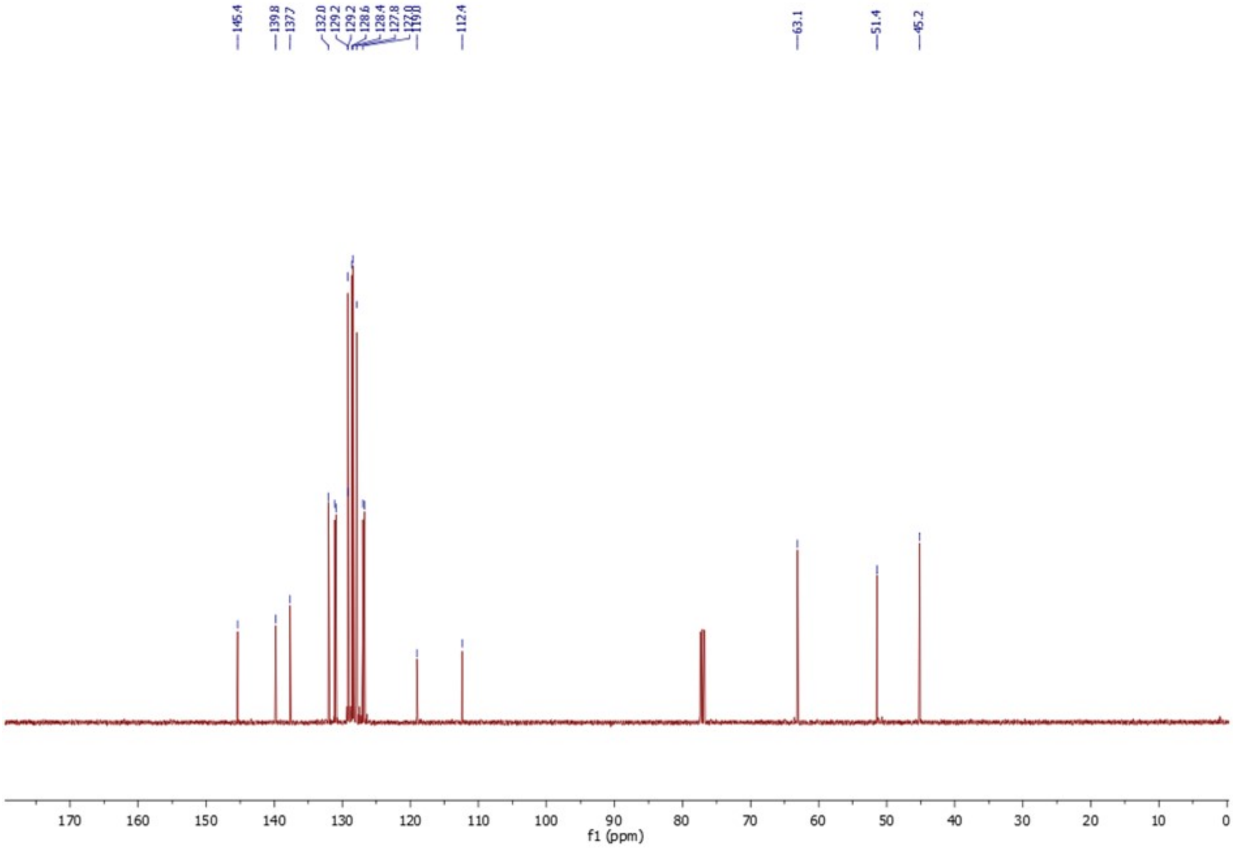
145.4
139.8 137.7
132.0 129.2 129.2 128.6 128.4 127.8 127.0 119.0
112.4
63.1
51.4
45.2
170 160 150 140 130 120 110 100 90 80 70 60 50 40 30 20 10 0
f1 (ppm)

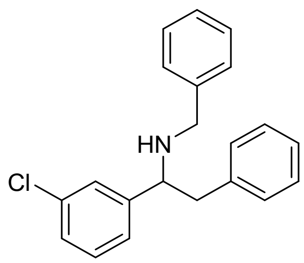

N-benzyl-1-(3-chlorophenyl)-2-phenylethanamide (**4h**) was obtained following the General procedure C.

^{1}H-NMR (400 MHz, CDCl$_3$): δ (ppm) 7.43 – 7.41 (m, 1H), 7.30 – 7.21 (m, 8H), 7.14 – 7.09 (m, 4H), 3.89 (dd, *J* = 8.6, 5.5 Hz, 1H), 3.68 (d, *J* = 13.6 Hz, 1H), 3.48 (d, *J* = 13.6 Hz, 1H), 2.96 (dd, *J* = 13.6, 5.5 Hz, 2H), 2.88 (dd, *J* = 13.6, 8.6 Hz, 2H), 2.01 (bs, 1H).

^{13}C-NMR (101 MHz, CDCl$_3$): δ (ppm) 145.9, 140.0, 138.2, 134.3, 129.6, 129.2, 128.4, 128.3, 127.9, 127.4, 127.3, 126.8, 126.5, 125.6, 63.2, 51.3, 45.1.

HRMS (ESI-TOF): mass calculated for $C_{21}H_{20}NCl$, 321.1284; m/z found, 322.1366 $[M+H]^+$.

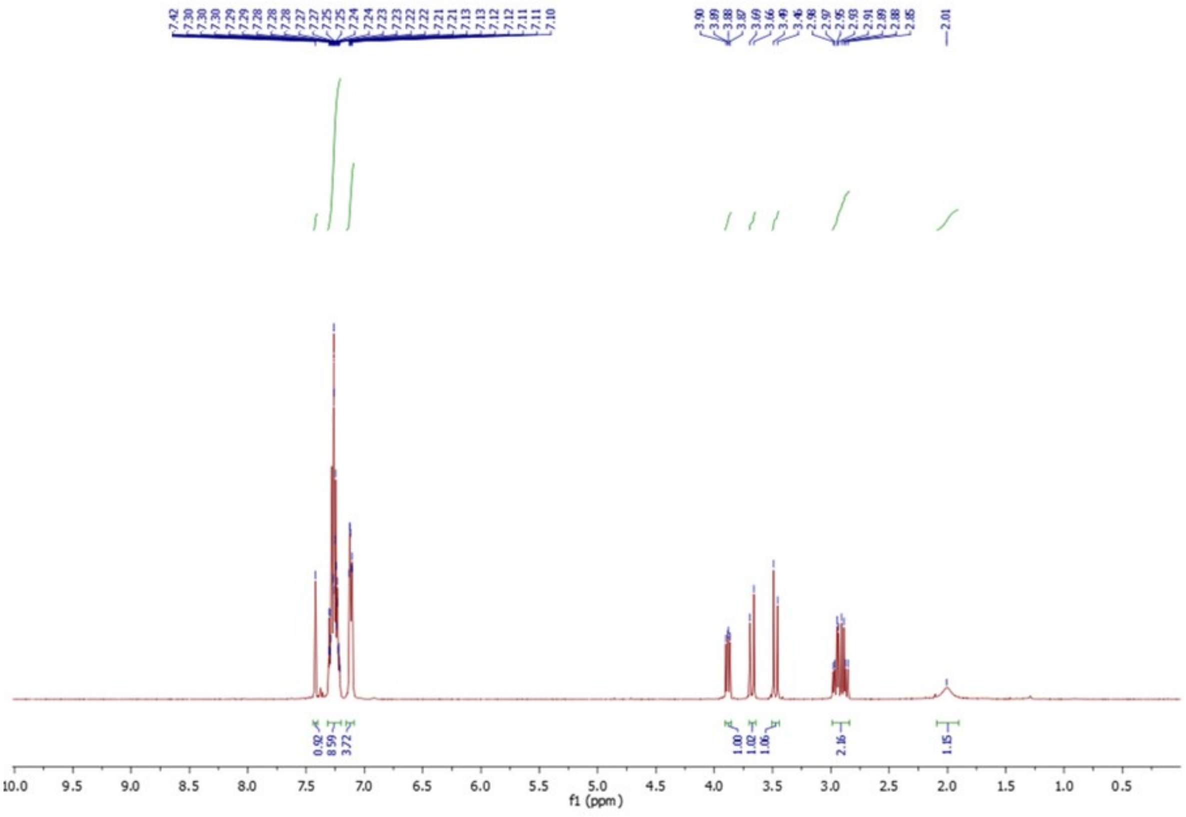

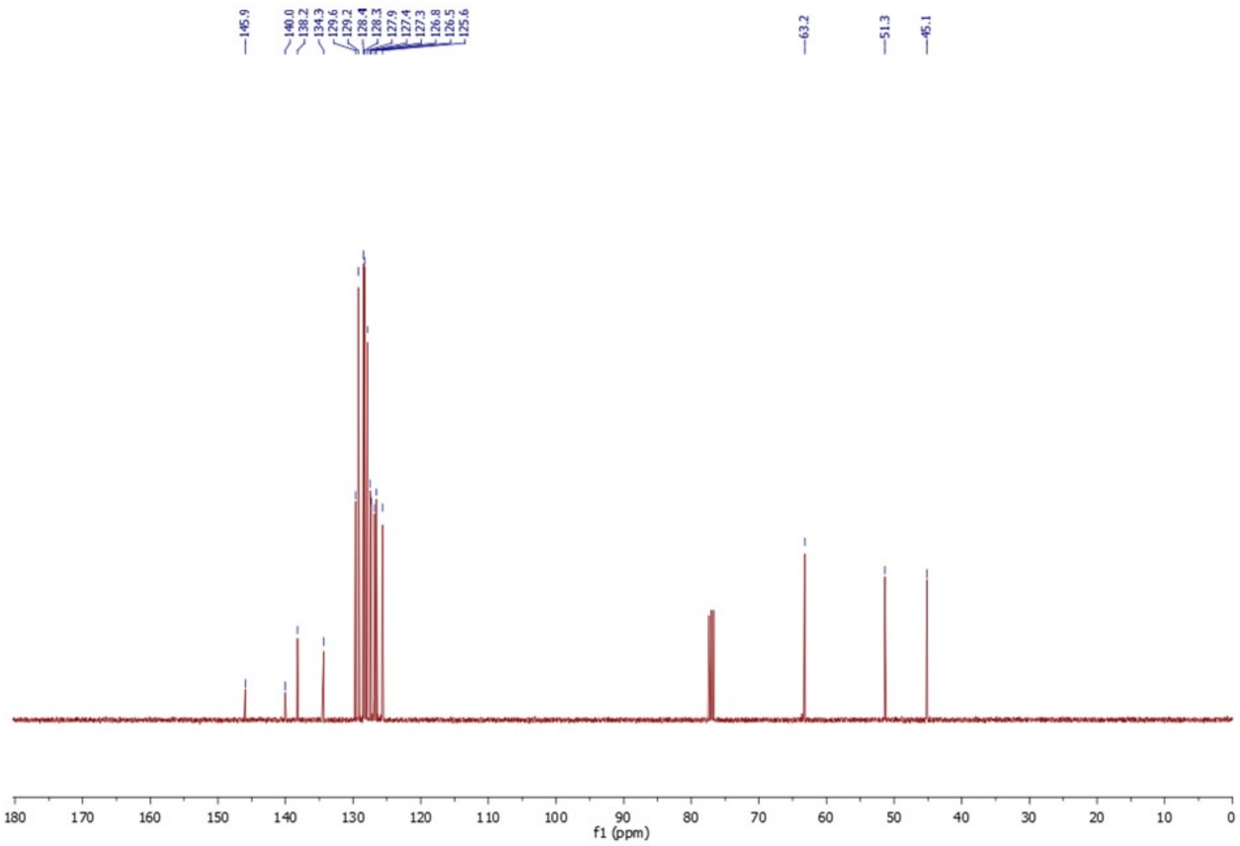

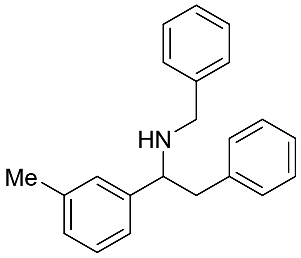

N-benzyl-2-phenyl-1-(m-tolyl)ethan-1-amine (**4i**) was obtained following the General procedure B.

^{1}H-NMR (400 MHz, $CDCl_3$): δ (ppm) 7.33 – 7.09 (m, 14H), 3.89 (dd, *J* = 8.7, 5.4 Hz, 1H), 3.72 (d, *J* = 13.6 Hz, 1H), 3.51 (d, *J* = 13.6 Hz, 1H), 3.01 (dd, *J* = 13.6, 5.4 Hz, 2H), 2.93 (dd, *J* = 13.6, 8.7 Hz, 2H), 2.40 (s, 3H), 2.33 (bs, 1H).

^{13}C-NMR (101 MHz, $CDCl_3$): δ (ppm) 143.5, 140.3, 138.9, 138.0, 129.3, 128.4, 128.3, 128.1, 128.0, 127.9, 126.8, 126.4, 124.5, 63.5, 51.3, 45.2, 21.5.

HRMS (ESI-TOF): mass calculated for $C_{22}H_{23}N$, 301.1830; m/z found, 302.1905 $[M+H]^+$.

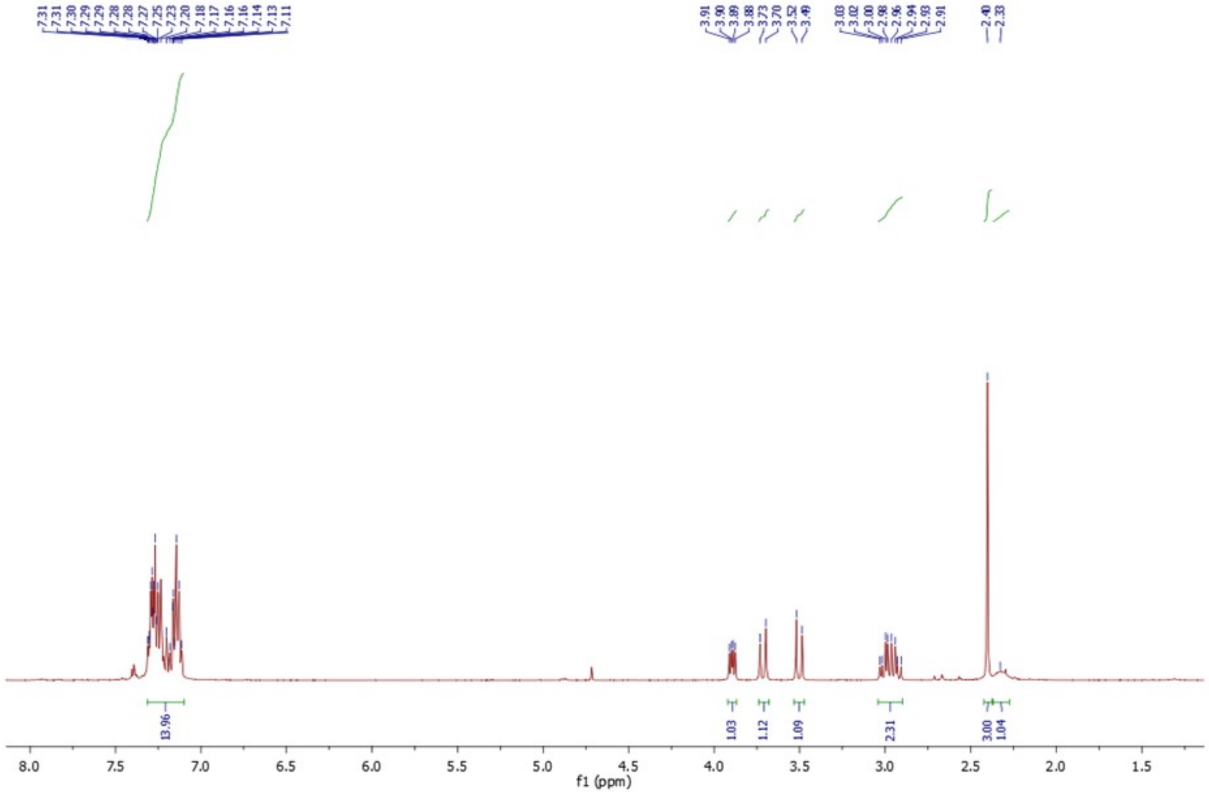
7.31 7.31 7.30 7.29 7.29 7.28 7.28 7.27 7.25 7.23 7.20 7.18 7.17 7.16 7.16 7.14 7.13 7.11
3.91 3.90 3.89 3.88 3.73 3.70 3.52 3.49
3.03 3.02 3.00 2.98 2.96 2.94 2.93 2.91
2.40 2.33
13.96
1.03
1.12
1.09
2.31
3.00
1.04
8.0 7.5 7.0 6.5 6.0 5.5 5.0 4.5 4.0 3.5 3.0 2.5 2.0 1.5
f1 (ppm)

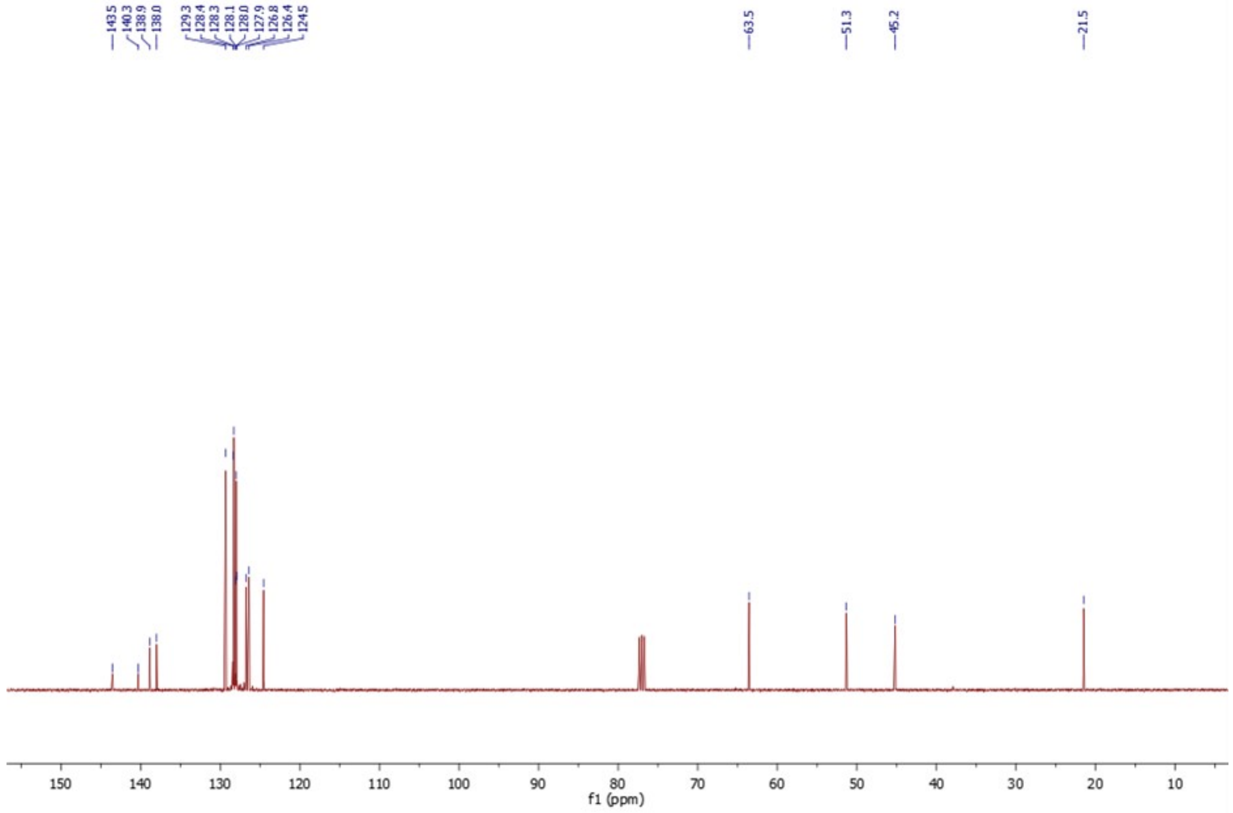
143.5 140.3 138.9 138.0
129.3 128.4 128.3 128.1 128.0 127.9 126.8 126.4 124.5
63.5
51.3
45.2
21.5
150 140 130 120 110 100 90 80 70 60 50 40 30 20 10
f1 (ppm)

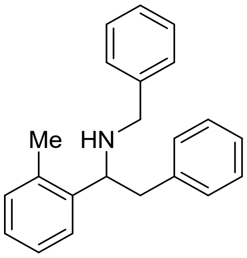

N-benzyl-2-phenyl-1-(o-tolyl)ethan-1-amine (**4j**) was obtained following General procedure B.

^{1}H-NMR (400 MHz, CDCl$_3$): δ (ppm) 7.71 (d, *J* = 7.6 Hz, 1H), 7.33 – 7.13 (m, 13H), 4.20 (dd, *J* = 8.5, 5.2 Hz, 1H), 3.70 (d, *J* = 13.5 Hz, 1H), 3.48 (d, *J* = 13.5 Hz, 1H), 2.97 (dd, *J* = 13.6, 5.2 Hz, 1H), 2.86 (dd, *J* = 13.5, 8.6 Hz, 1H), 2.18 (s, 3H), 1.90 (bs, 1H).

^{13}C-NMR (101 MHz, CDCl$_3$): δ (ppm) 141.5, 140.5, 138.9, 136.0, 130.3, 129.2, 128.4, 128.3, 128.0, 126.7, 126.6, 126.4, 126.2, 58.7, 51.3, 44.3, 19.1.

HRMS (ESI-TOF): mass calculated for $C_{22}H_{23}N$, 301.1830; m/z found, 302.1908 $[M+H]^{+}$.

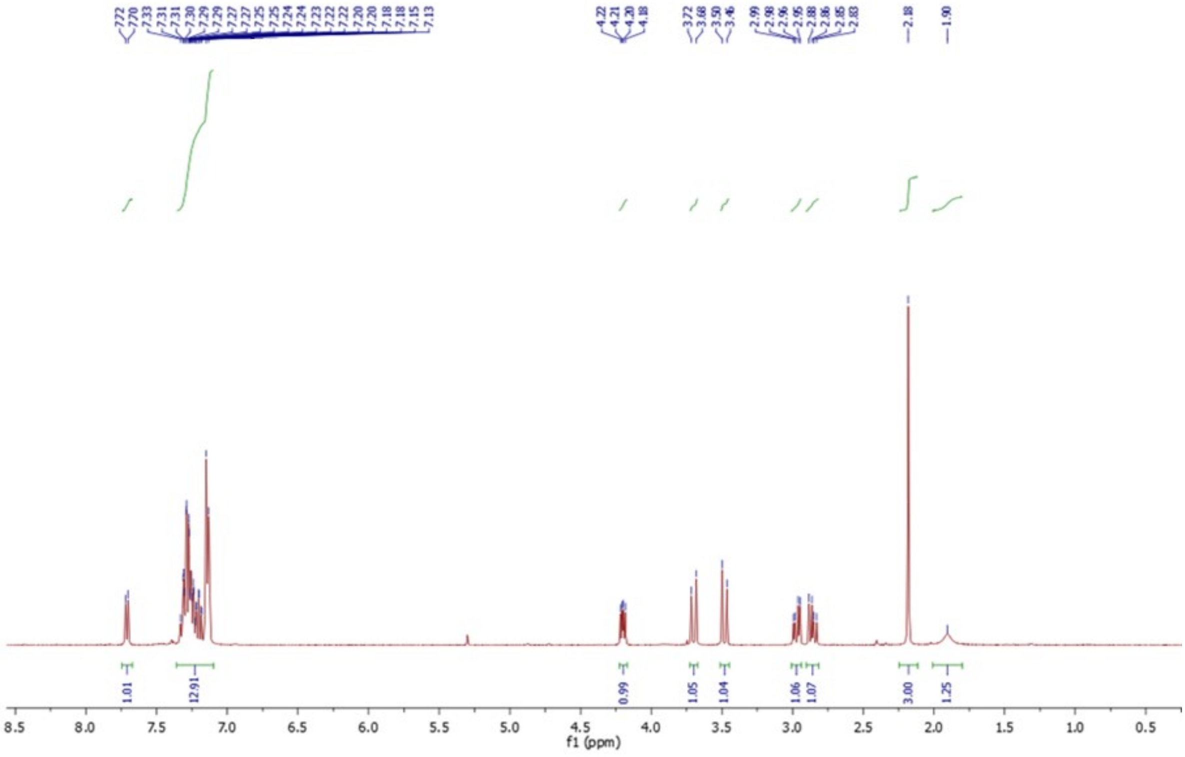

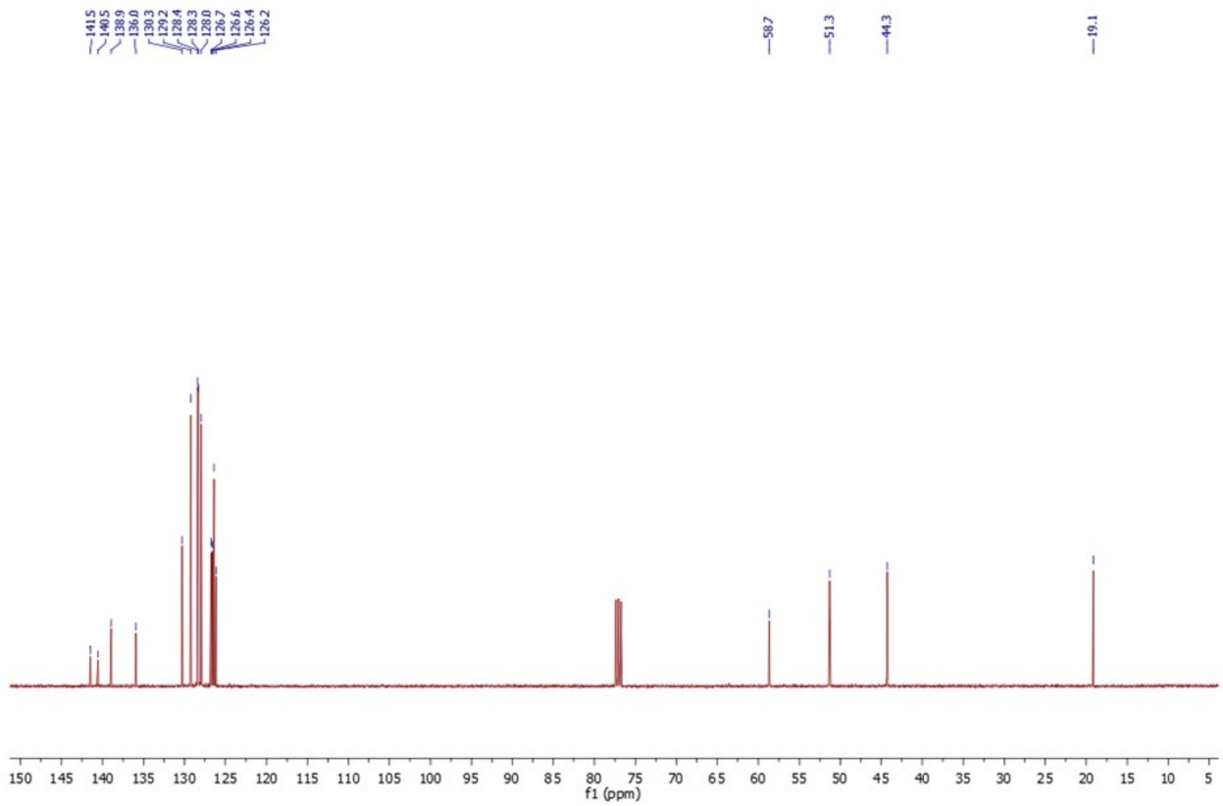

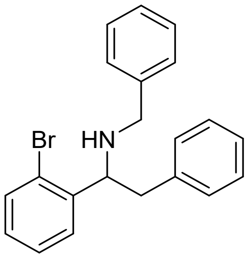

N-benzyl-1-(2-bromophenyl)-2-phenylethanamide (**4k**) was obtained following General procedure C.

^{1}H-NMR (400 MHz, $CDCl_3$): δ (ppm) 7.74 (d, *J* = 7.8 Hz, 1H), 7.57 (dd, *J* = 7.8, 1.1 Hz, 1H), 7.37 (d, *J* = 7.8 Hz, 2H), 7.33 – 7.11 (m, 10H), 4.44 (dd, *J* = 9.6, 4.0 Hz, 1H), 3.63 (d, *J* = 13.4 Hz, 1H), 3.44 (d, *J* = 13.4 Hz, 1H), 3.12 (dd, *J* = 13.7, 4.0 Hz, 1H), 2.66 (dd, *J* = 13.7, 9.6 Hz, 1H),1.92 (bs, 1H).

^{13}C-NMR (101 MHz, $CDCl_3$): δ (ppm) 142.2, 142.1, 138.5, 138.2, 129.2, 128.5, 128.4, 128.3, 128.0, 127.9, 127.7, 127.4, 127.2, 126.8, 126.7, 126.5, 126.3, 124.1, 62.0, 51.5, 43.3.

HRMS (ESI-TOF): mass calculated for $C_{21}H_{20}NBr$, 365.0779; m/z found, 366.0878 $[M+H]^+$.

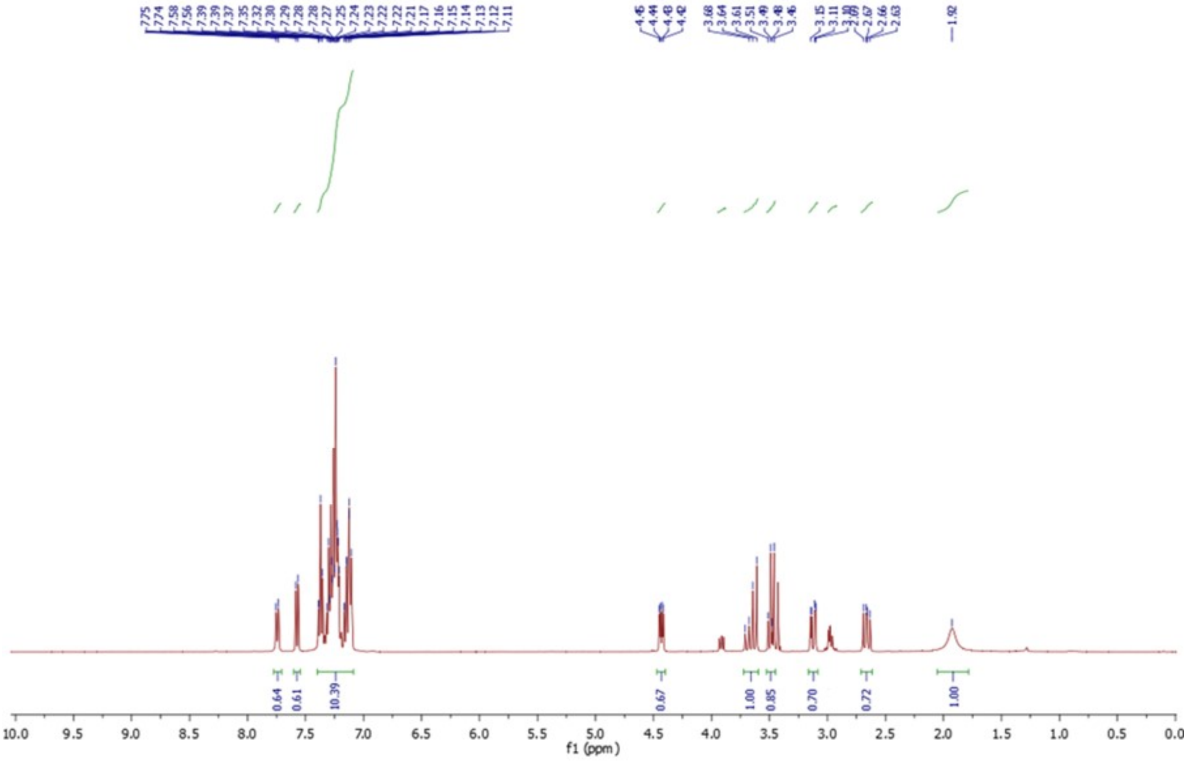
7.75 7.74 7.58 7.56 7.39 7.39 7.37 7.35 7.32 7.30 7.29 7.28 7.28 7.27 7.25 7.24 7.23 7.22 7.22 7.21 7.17 7.16 7.15 7.14 7.13 7.12 7.11
4.45 4.44 4.43 4.42
3.68 3.64 3.61 3.51 3.49 3.48 3.46
3.15 3.11 3.10
2.67 2.66 2.63
1.92
0.64
0.61
10.39
0.67
1.00
0.85
0.70
0.72
1.00
f1 (ppm)

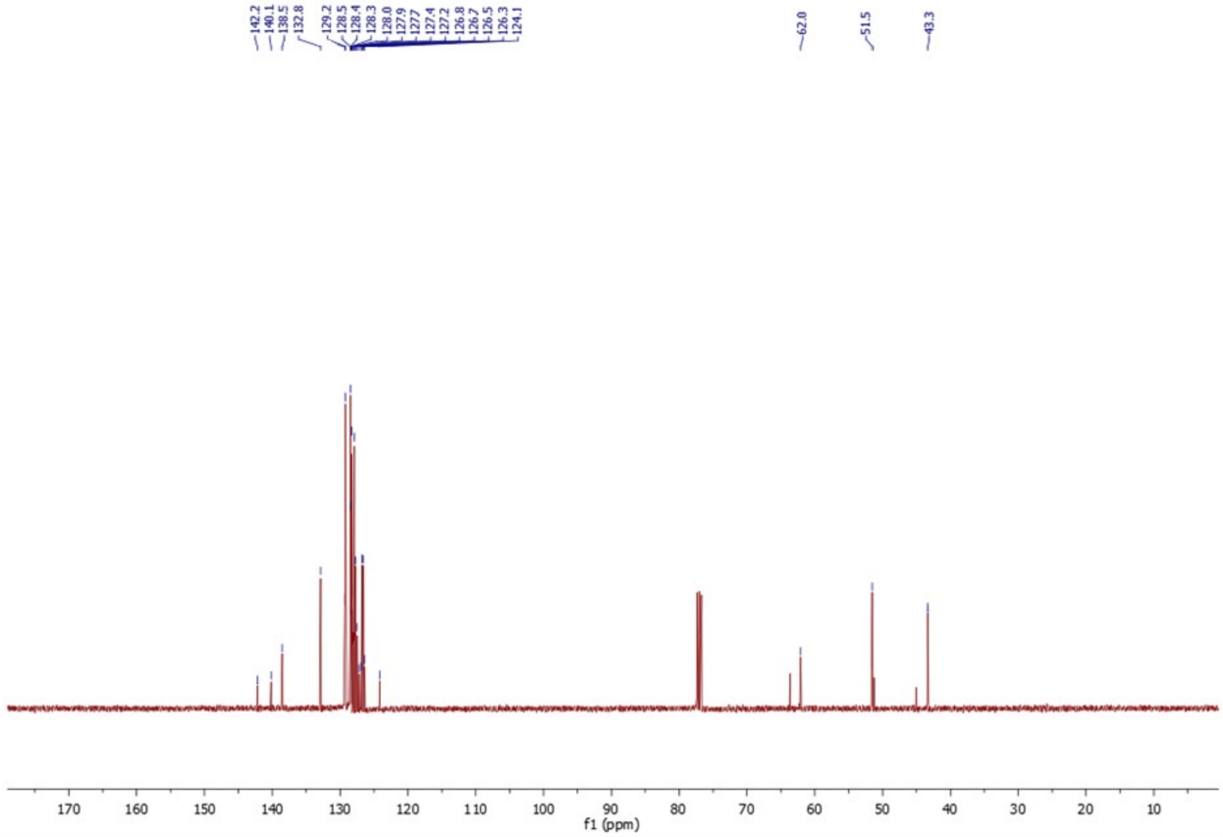
142.2 140.1 138.5 132.8
129.2 128.5 128.4 128.3 128.0 127.9 127.7 127.4 127.2 126.8 126.7 126.5 126.3 124.1
62.0
51.5
43.3
f1 (ppm)

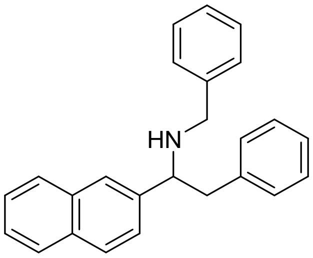

N-benzyl-1-(naphthalen-2-yl)-2-phenylethanamide (**4l**) was obtained following General procedure B.

^{1}H-NMR (400 MHz, $CDCl_3$): δ (ppm) 7.92 – 7.84 (m, 3H), 7.82 (s, 1H), 7.63 (dd, *J* = 8.5, 1.7 Hz, 1H), 7.61 – 7.49 (m, 2H), 7.33 – 7.24 (m, 6H), 7.20 – 7.15 (m, 4H), 4.13 (dd, *J* = 8.3, 5.8 Hz, 1H), 3.75 (d, *J* = 13.6 Hz, 1H), 3.56 (d, *J* = 13.6 Hz, 1H), 3.12 (dd, *J* = 13.6, 5.8 Hz, 1H), 3.06 (dd, *J* = 13.6, 8.3 Hz, 1H), 2.79 (bs, 1H).

^{13}C-NMR (101 MHz, $CDCl_3$): δ (ppm) 140.8, 140.1, 138.6, 133.5, 133.0, 129.3, 128.5, 2x 128.3, 128.1, 127.9, 127.7, 126.9, 2x 126.5, 126.0, 125.6, 125.5, 63.7, 51.3, 45.0.

HRMS (ESI-TOF): mass calculated for $C_{25}H_{23}N$, 337.1864; m/z found, 338.1912 $[M+H]^+$.

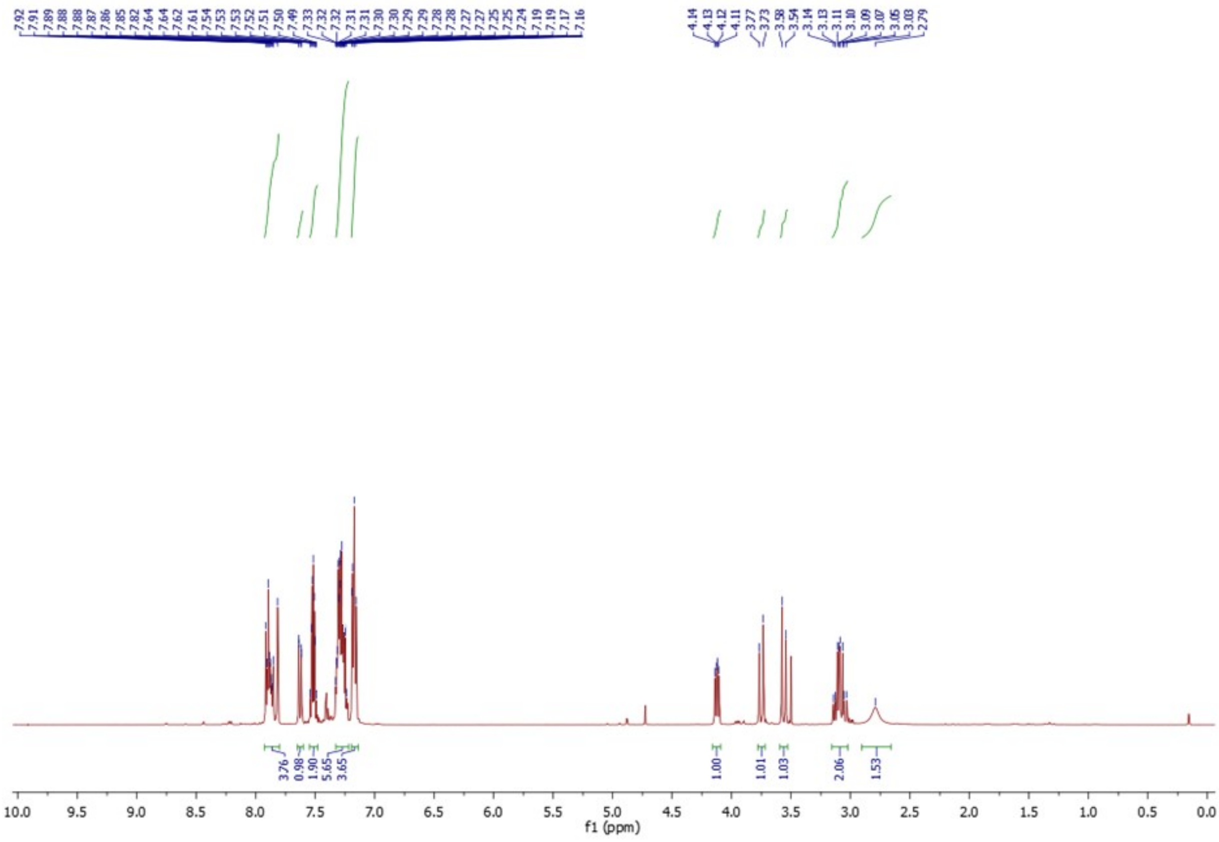

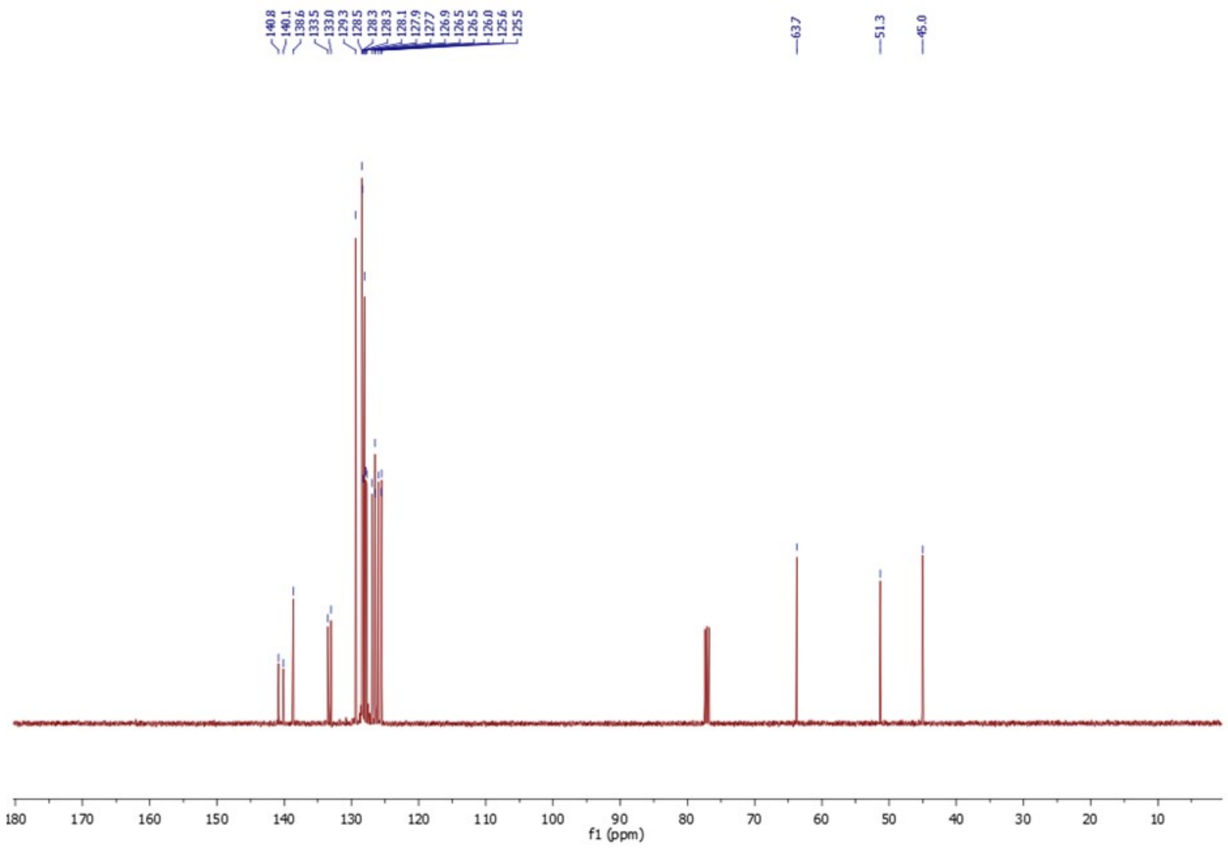

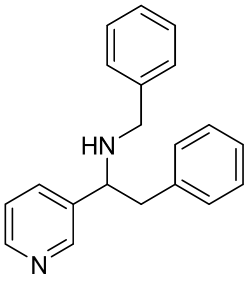

N-benzyl-2-phenyl-1-(pyridin-3-yl)ethan-1-amine (**4m**) was obtained following General procedure B.

^{1}H-NMR (400 MHz, CDCl$_3$): δ (ppm) 8.49 (s, 2H), 7.75 (d, *J* = 9.3 Hz, 1H), 7.31 – 7.15 (m, 7H), 7.11 – 7.05 (m, 4H), 3.93 (t, *J* = 7.1 Hz, 1H), 3.65 (d, *J* = 13.5 Hz, 1H), 3.48 (d, *J* = 13.5 Hz, 1H), 3.02 (bs, 1H), 2.95 (d, *J* = 7.1 Hz, 2H).

^{13}C-NMR (101 MHz, CDCl$_3$): δ (ppm) 149.5, 148.8, 147.9, 139.8, 133.7, 129.3, 129.2, 128.6, 128.5, 128.4, 128.3, 128.2, 127.9, 127.5, 127.0, 126.8, 126.7, 61.2, 51.3, 45.0.

HRMS (ESI-TOF): mass calculated for $C_{20}H_{20}N_2$, 288.1626; m/z found, 289.1705 [M+H]$^+$.

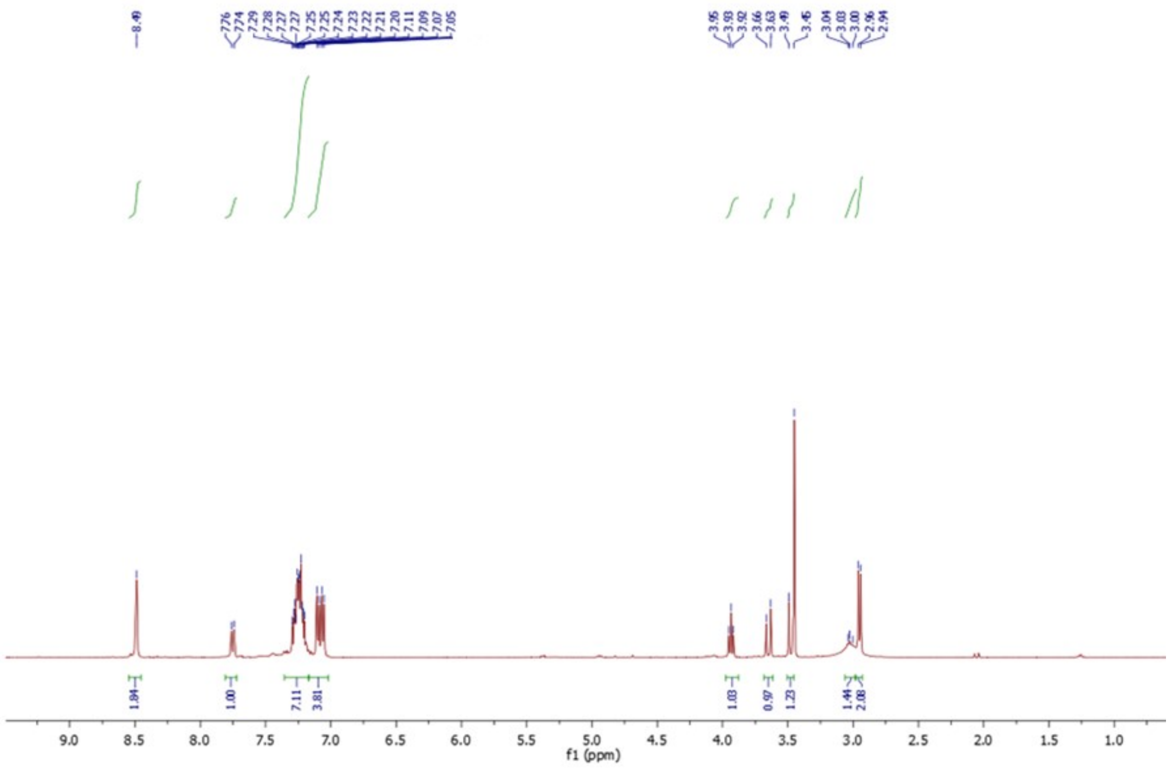
8.49
7.76 7.74 7.29 7.28 7.27 7.27 7.25 7.25 7.24 7.23 7.22 7.21 7.20 7.11 7.09 7.07 7.05
3.95 3.93 3.92 3.66 3.63 3.49 3.45 3.04 3.03 3.00 2.96 2.94
1.84 1.00 7.11 3.81 1.03 0.97 1.23 1.44 2.08
f1 (ppm)

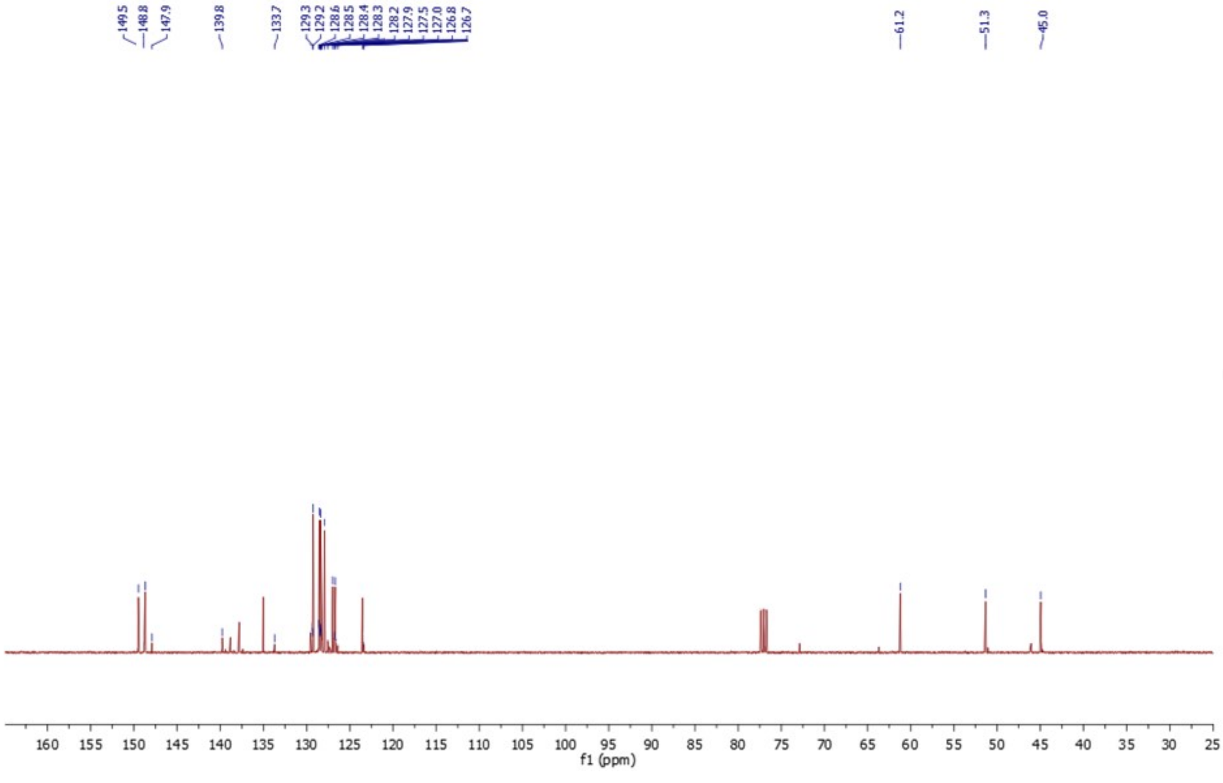
149.5 148.8 147.9 139.8 133.7 129.3 129.2 128.6 128.5 128.4 128.3 128.2 127.9 127.5 127.0 126.8 126.7
61.2 51.3 45.0
f1 (ppm)

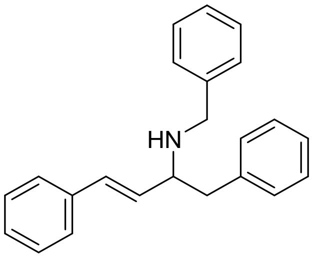

(*E*)-*N*-benzyl-1,4-diphenylbut-3-en-2-amine (**4n**) was obtained following General procedure B.

^{1}H-NMR (400 MHz, $CDCl_3$): δ (ppm) 7.42 – 7.19 (m, 15H), 6.49 (d, *J* = 15.9 Hz, 1H), 6.18 (dd, *J* = 15.9, 8.0 Hz, 1H), 3.91 (d, *J* = 13.6 Hz, 1H), 3.70 (d, *J* = 13.6 Hz, 1H), 3.53 (td, *J* = 8.0, 5.8 Hz, 1H), 2.95 (dd, *J* = 13.5, 5.8 Hz, 1H), 2.89 (dd, *J* = 13.5, 8.0 Hz, 1H), 2.34 (bs, 1H).

^{13}C-NMR (101 MHz, $CDCl_3$): δ (ppm) 140.1, 138.3, 137.0, 132.2, 131.6, 129.5, 128.6, 128.5, 128.4, 128.0, 127.5, 126.9, 2x 126.4, 61.3, 51.2, 42.7.

HRMS (ESI-TOF): mass calculated for $C_{23}H_{23}N$, 313.1830; m/z found, 314.1909 $[M+H]^+$.

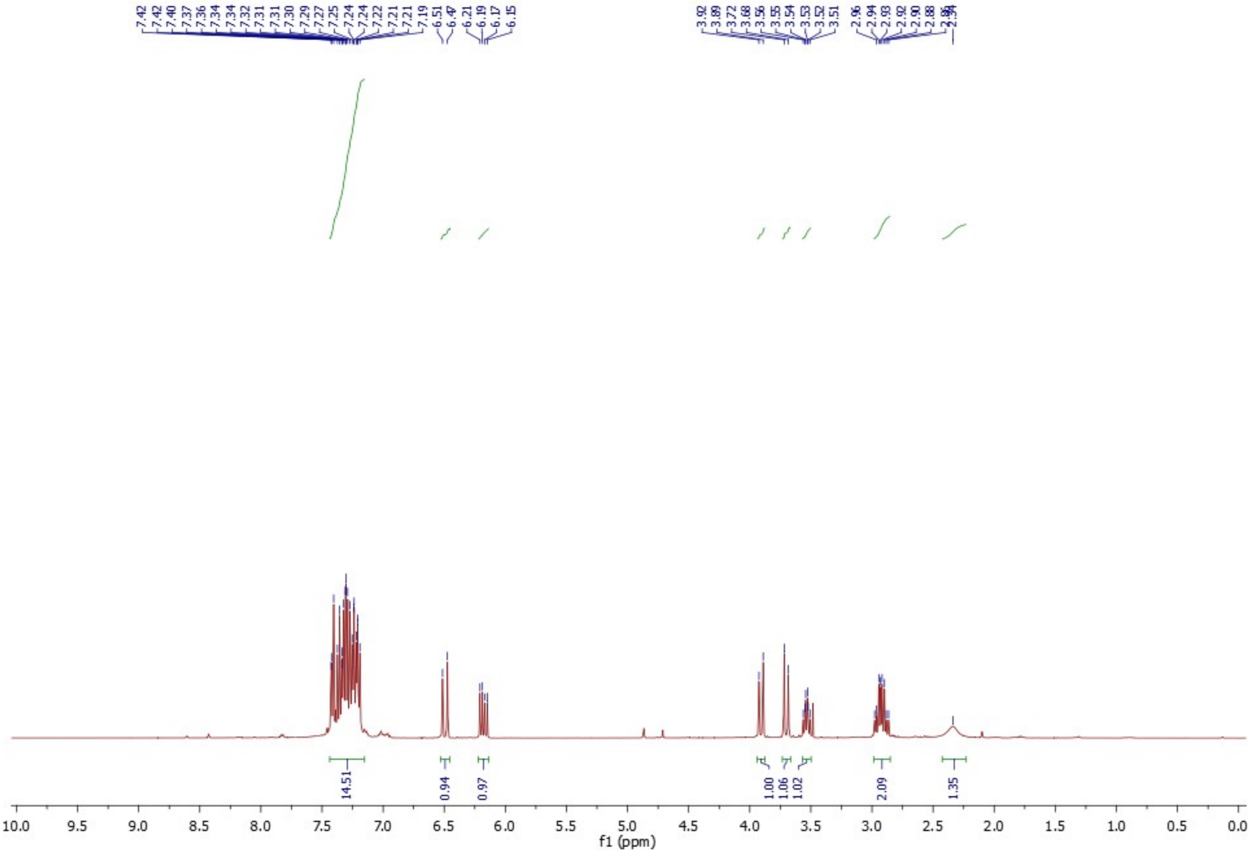

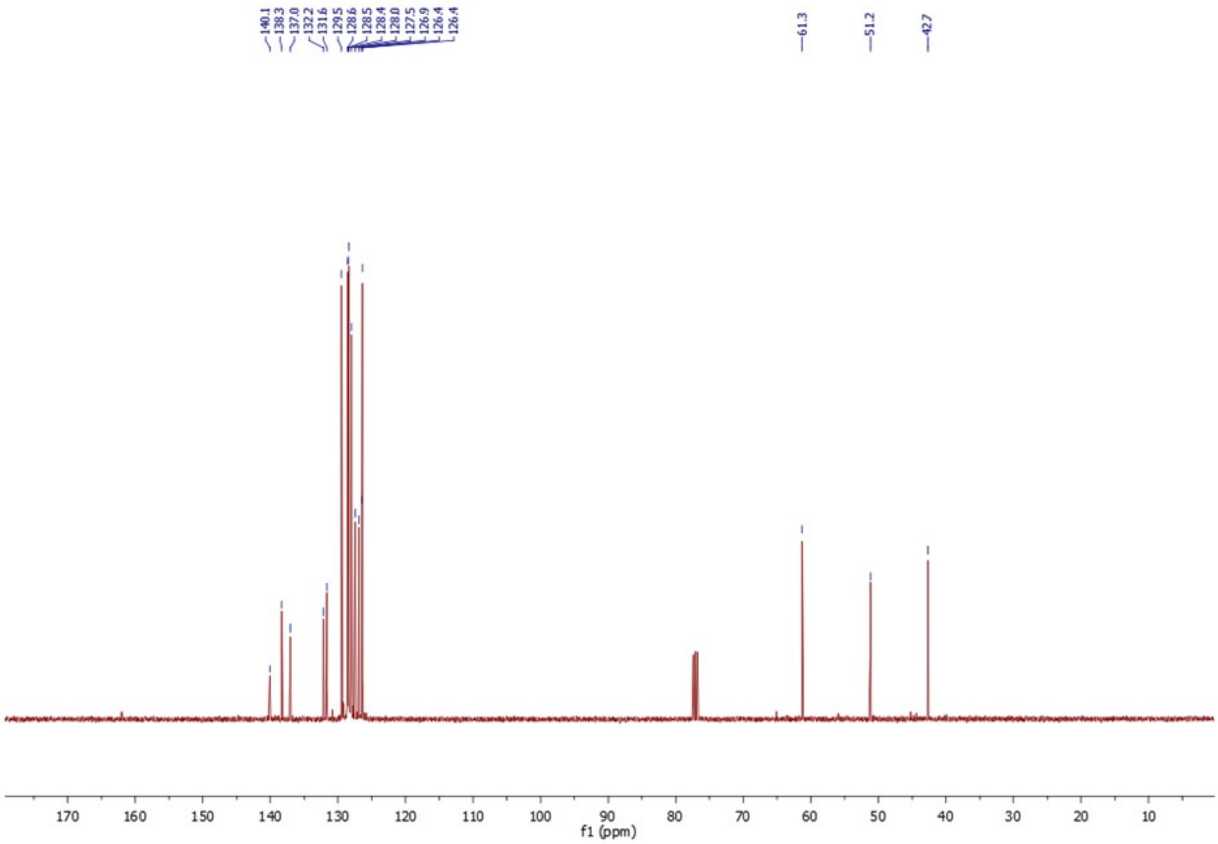

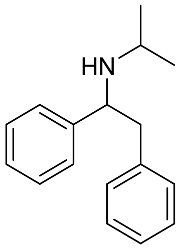

N-(1,2-diphenylethyl)propan-2-amine (**4o**) was obtained following General procedure B.

^{1}H-NMR (400 MHz, CDCl$_3$): δ (ppm) 7.31 – 7.06 (m, 10H), 4.04 (t, *J* = 7.0 Hz, 1H), 3.06 – 2.96 (m, 2H), 2.69 – 2.60 (m, 1H), 1.02 (dd, *J* = 6.4 Hz, 3H), 0.97 (d, *J* = 6.2 Hz, 3H).

^{13}C-NMR (101 MHz, CDCl$_3$): δ (ppm) 143.1, 138.6, 129.3, 128.3, 128.2, 127.4, 127.1, 126.2, 61.9, 2x 45.0, 23.8, 21.4.

HRMS (ESI-TOF): mass calculated for $C_{17}H_{21}N$, 239.1674; m/z found, 240.1744 $[M+H]^+$.

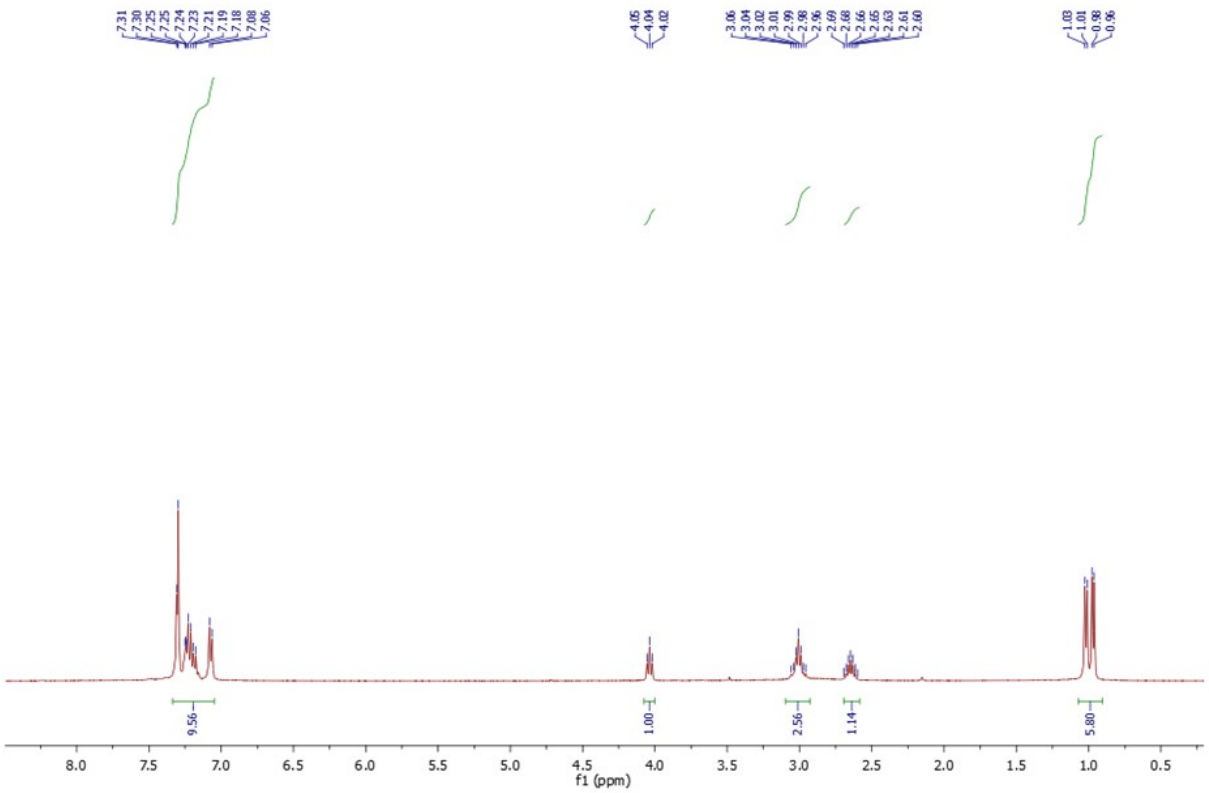
7.31
7.30
7.25
7.25
7.24
7.23
7.21
7.19
7.18
7.08
7.06
4.05
4.04
4.02
3.06
3.04
3.02
3.01
2.99
2.98
2.96
2.69
2.68
2.66
2.65
2.63
2.61
2.60
1.03
1.01
0.98
0.96
9.56
1.00
2.56
1.14
5.80
f1 (ppm)

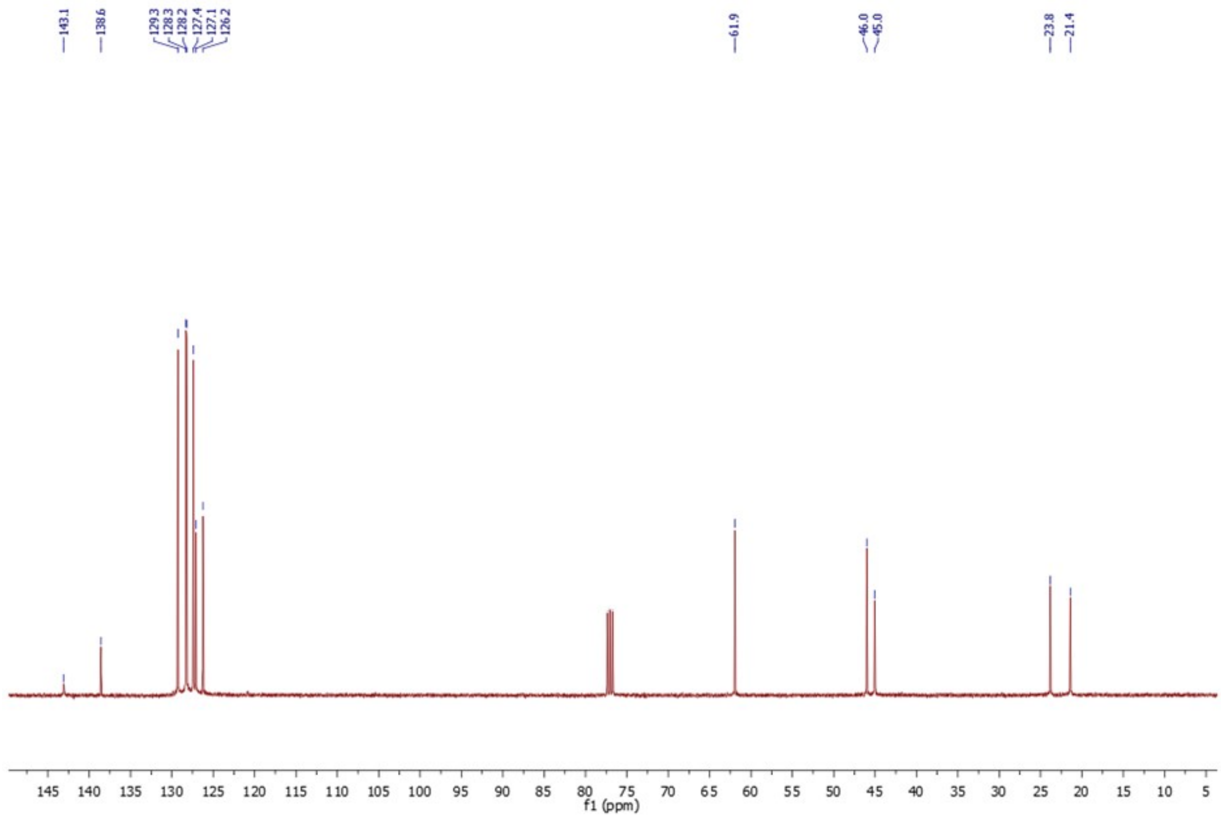
143.1
138.6
129.3
128.3
128.2
127.4
127.1
126.2
61.9
46.0
45.0
23.8
21.4
f1 (ppm)

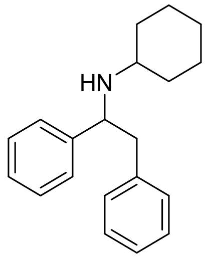

N-(1,2-diphenylethyl)cyclohexanamine (**4p**) was obtained following General procedure C.

^{1}H-NMR (400 MHz, $CDCl_3$): δ (ppm) 7.33 – 7.28 (m, 4H), 7.27 – 7.21 (m, 3H), 7.21 – 7.16 (m, 1H), 7.13 – 7.08 (m, 2H), 4.08 (t, *J* = 7.1 Hz, 1H), 2.99 – 2.89 (m, 2H), 2.31 – 2.21 (m, 1H), 1.86 (d, *J* = 11.2 Hz, 1H), 1.71 (d, *J* = 11.2 Hz, 1H), 1.63 – 1.45 (m, 3H), 1.14 – 0.97 (m, 4H), 0.94 – 0.82 (m, 1H).

^{13}C-NMR (101 MHz, $CDCl_3$): δ (ppm) 143.8, 138.8, 129.2, 128.3, 127.3, 127.0, 126.2, 61.2, 53.6, 45.3, 34.4, 32.3, 26.0, 25.1, 24.7.

HRMS (ESI-TOF): mass calculated for $C_{20}H_{25}N$, 279.1987; m/z found, 280.2063 $[M+H]^+$.

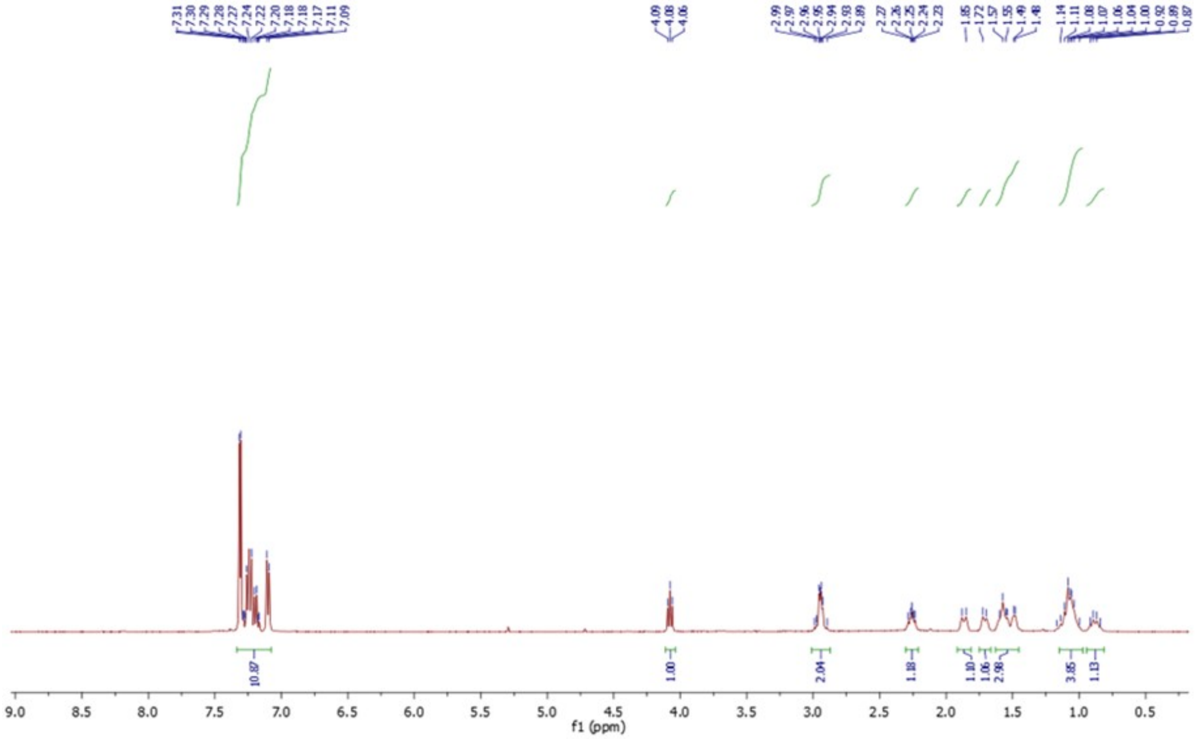

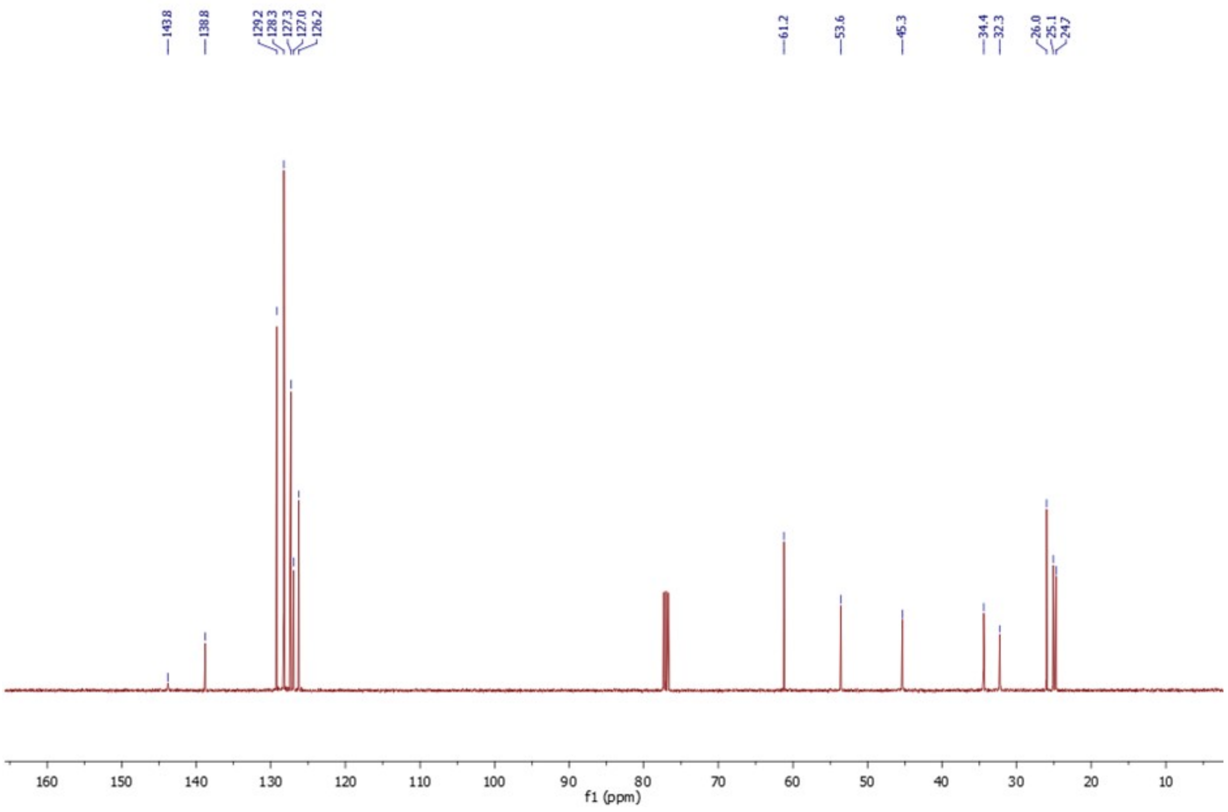

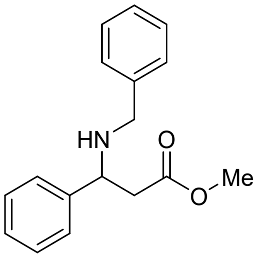

Methyl 3-(benzylamino)-3-phenylpropanoate (**4q**) was obtained following General procedure B.

^{1}H-NMR (400 MHz, CDCl$_3$): δ (ppm) 7.38 – 7.24 (m, 10H), 4.13 (dd, *J* = 8.8, 5.2 Hz, 1H), 3.68 (d, *J* = 13.2 Hz, 1H), 3.64 (s, 3H), 3.55 (d, *J* = 13.2 Hz, 1H), 2.76 (dd, *J* = 15.6, 8.8 Hz, 1H), 2.65 (dd, *J* = 15.6, 5.2 Hz, 1H), 2.54 (bs, 1H).

^{13}C-NMR (101 MHz, CDCl$_3$): δ (ppm) 172.2, 142.3, 140.0, 128.6, 128.3, 128.2, 127.6, 127.2, 126.9, 58.8, 51.6, 51.2, 42.8.

HRMS (ESI-TOF): mass calculated for $C_{17}H_{19}NO_2$, 269.1416; m/z found, 270.1491 $[M+H]^+$.

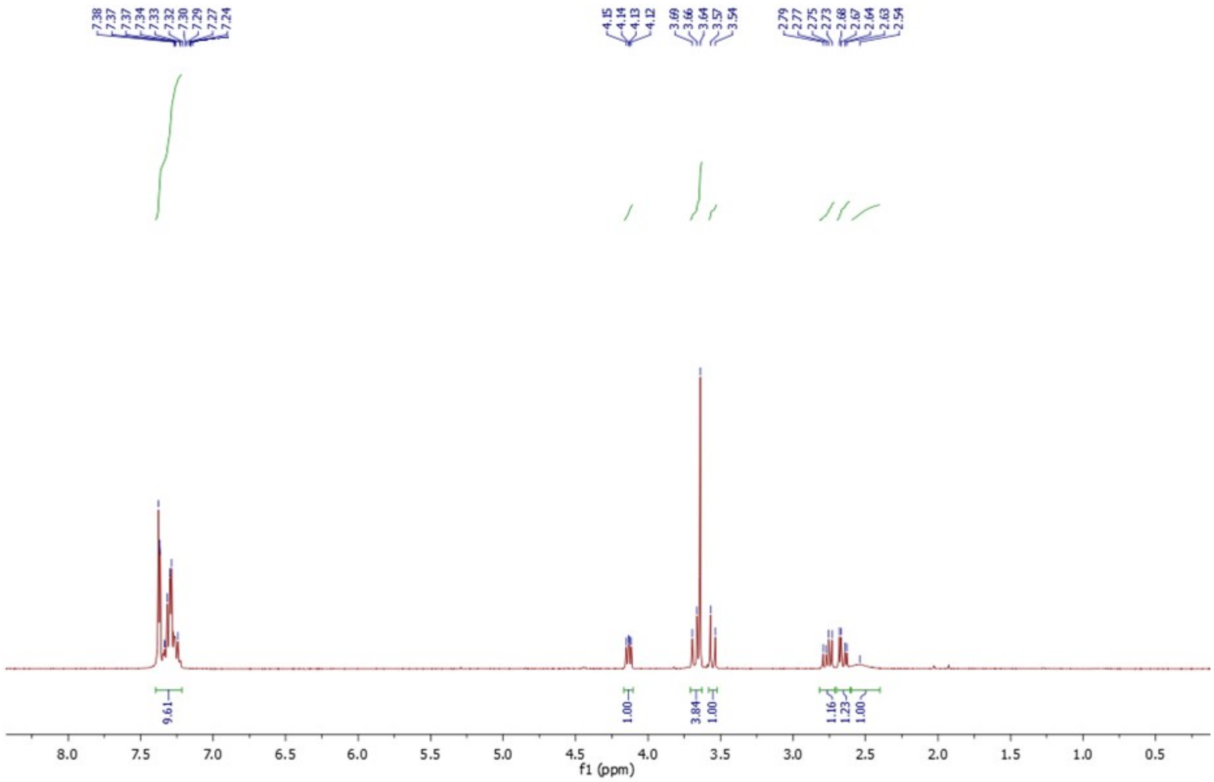
7.38 7.37 7.37 7.34 7.33 7.32 7.30 7.29 7.27 7.24
4.15 4.14 4.13 4.12
3.69 3.66 3.64 3.57 3.54
2.79 2.77 2.75 2.73 2.68 2.67 2.64 2.63 2.54
9.61
1.00
3.84
1.00
1.16
1.23
1.00
f1 (ppm)

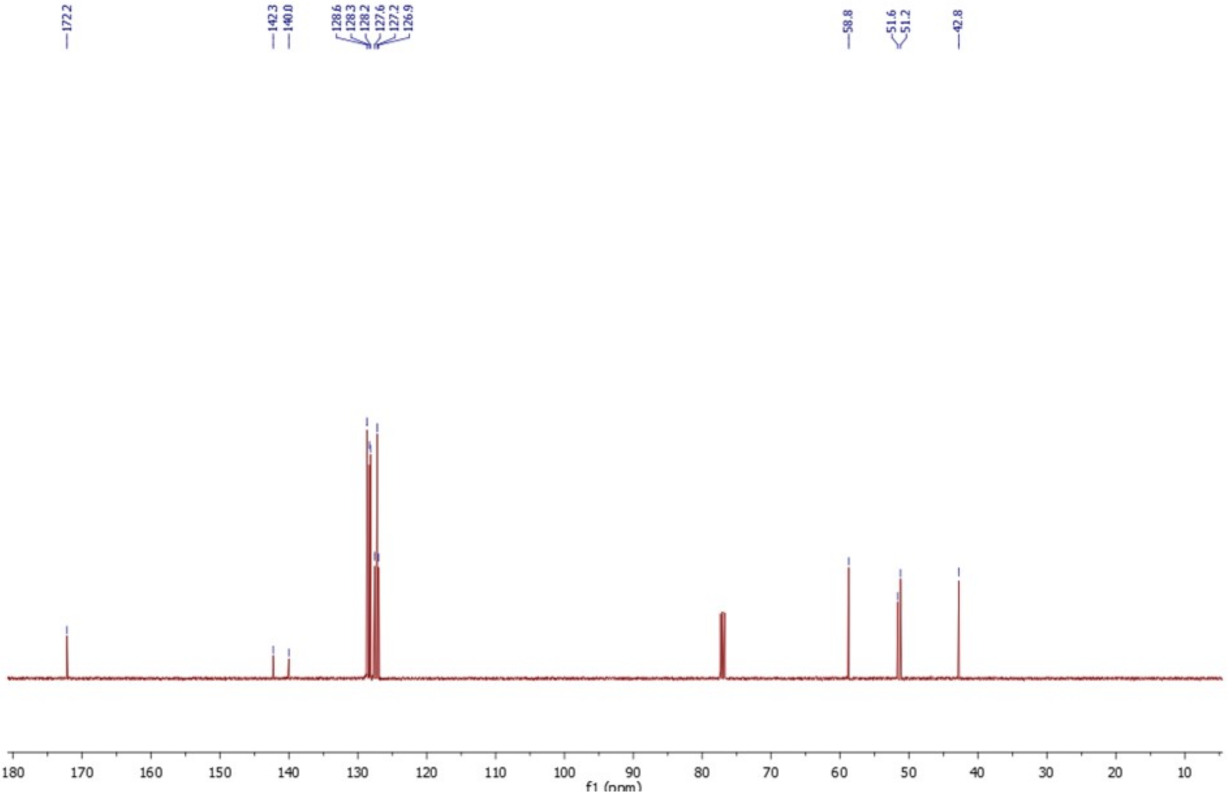
172.2
142.3
140.0
128.6 128.3 128.2 127.6 127.2 126.9
58.8
51.6 51.2
42.8
f1 (ppm)

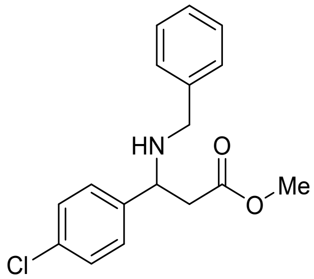

Methyl 3-(benzylamino)-3-(4-chlorophenyl)propanoate (**4r**) was obtained following General procedure B.

^{1}H-NMR (400 MHz, $CDCl_3$): δ (ppm) 7.35 – 7.25 (m, 9H), 4.10 (dd, *J* = 8.3, 5.0 Hz, 1H), 3.65 (d , *J* = 13.2, 1H), 3.64 (s, 3H), 3.53 (d, *J* = 13.2, 1H), 3.72 (dd, *J* = 15.7, 8.3 Hz, 1H), 2.61 (dd, *J* = 15.7, 5.0 Hz, 1H), 2.47 (bs, 1H).

^{13}C-NMR (101 MHz, $CDCl_3$): δ (ppm) 171.9, 140.9, 139.8, 133.2, 128.8, 128.6, 128.4, 128.1, 127.0, 58.1, 51.7, 51.2, 42.6.

HRMS (ESI-TOF): mass calculated for $C_{17}H_{18}NO_2Cl$, 303.1026; m/z found, 304.1099 $[M+H]^+$.

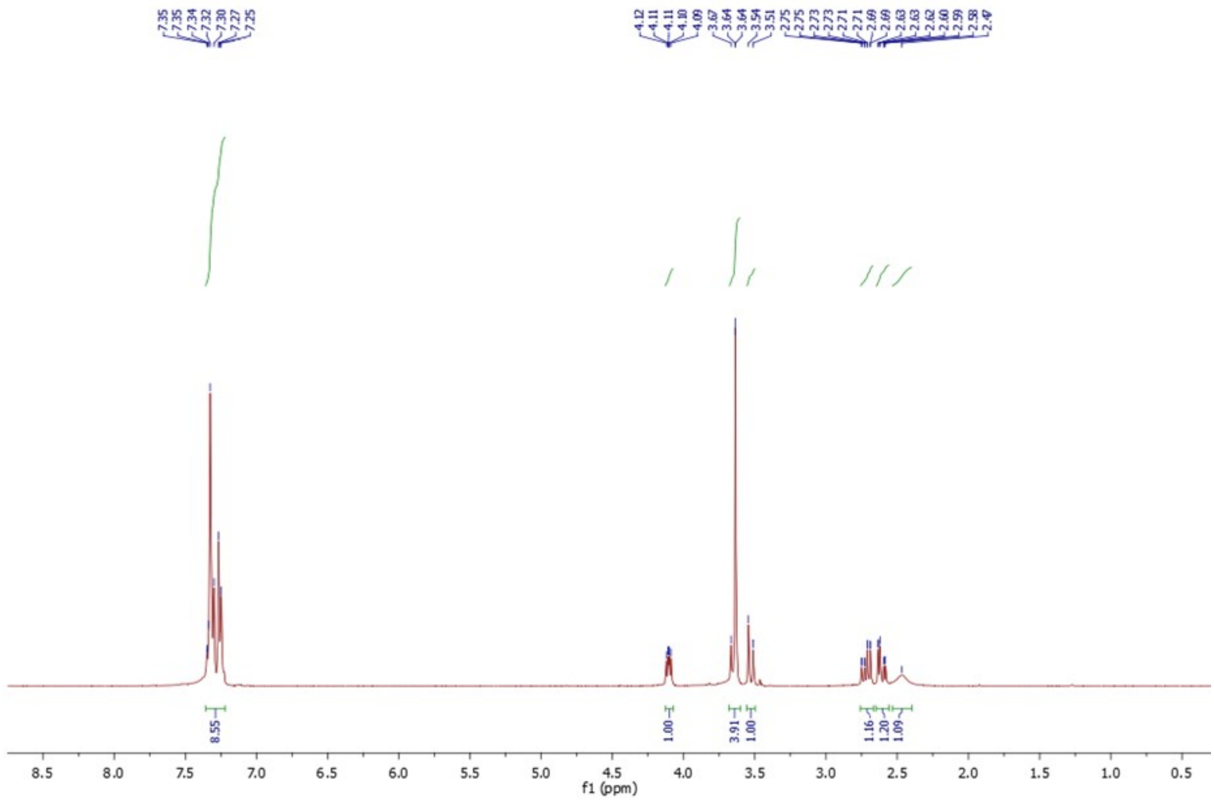

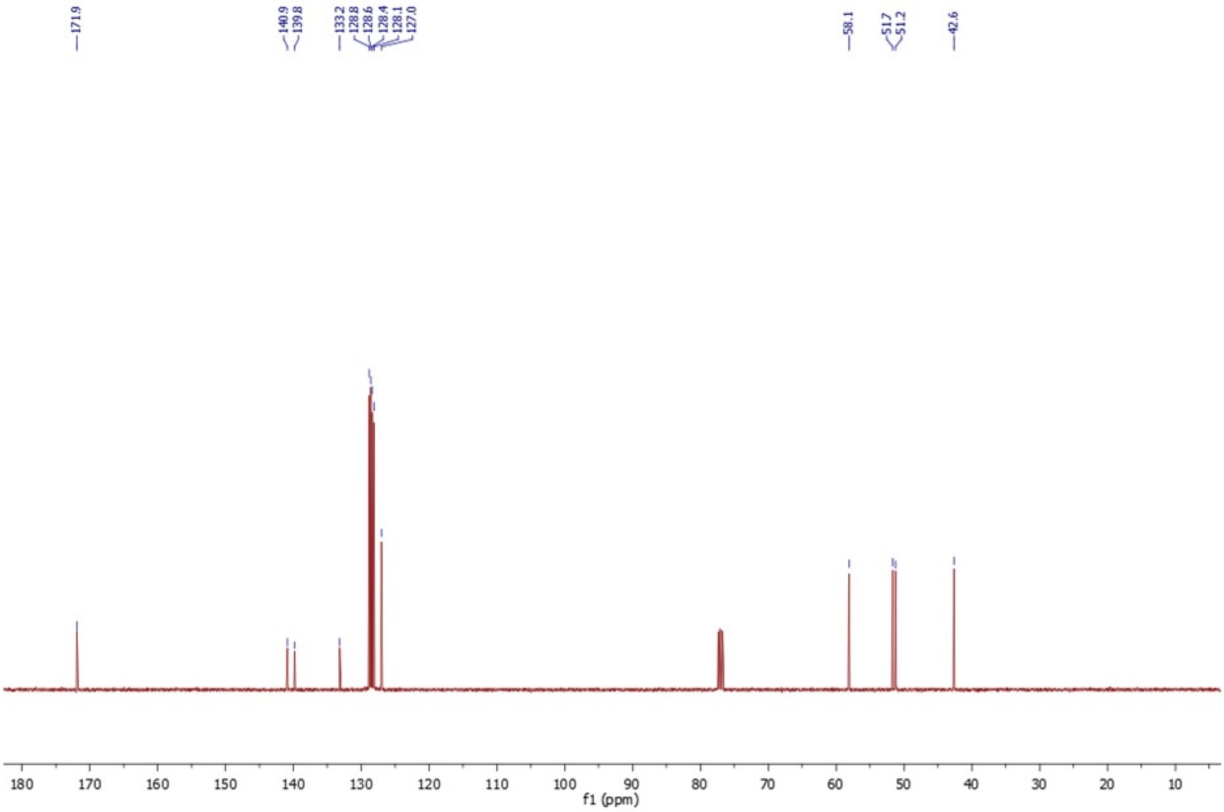

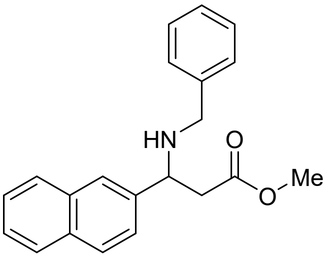

Methyl 3-(benzylamino)-3-(naphthalen-2-yl)propanoate (**4s**) was obtained following General procedure B.

^{1}H-NMR (400 MHz, CDCl$_3$): δ (ppm) 7.88 – 7.80 (m, 4H), 7.59 – 7.48 (m, 3H), 7.34 – 7.25 (m, 5H), 4.30 (dd, *J* = 8.7, 5.2 Hz, 1H), 3.70 (d, *J* = 13.2 Hz, 1H), 3.64 (s, 3H), 3.58 (d, *J* = 13.2 Hz, 1H) 2.84 (dd, *J* = 15.6, 8.7 Hz, 1H), 2.73 (dd, *J* = 15.7, 5.2 Hz, 1H), 2.57 (bs, 1H).

^{13}C-NMR (101 MHz, CDCl$_3$): δ (ppm) 172.2, 140.1, 139.7, 133.4, 133.1, 128.6, 128.4, 128.2, 127.8, 127.7, 127.0, 126.3, 126.1, 125.8, 124.9, 58.9, 51.7, 51.3, 42.7.

HRMS (ESI-TOF): mass calculated for $C_{21}H_{21}NO_2$, 319.1572; m/z found, 320.1652 [M+H]$^+$.

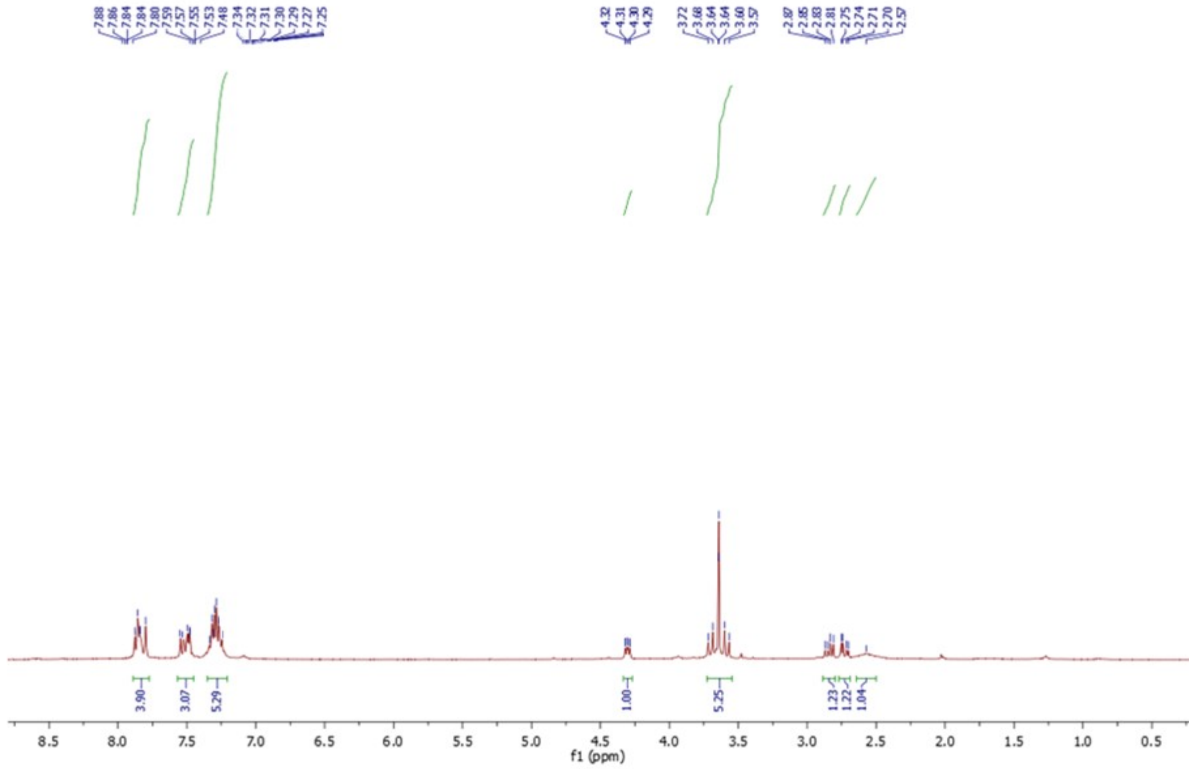
7.88
7.86
7.84
7.84
7.80
7.59
7.57
7.55
7.53
7.48
7.34
7.32
7.31
7.30
7.29
7.27
7.25
4.32
4.31
4.30
4.29
3.72
3.68
3.64
3.64
3.60
3.57
2.87
2.85
2.83
2.81
2.75
2.74
2.71
2.70
2.57
3.90
3.07
5.29
1.00
5.25
1.23
1.22
1.04
f1 (ppm)

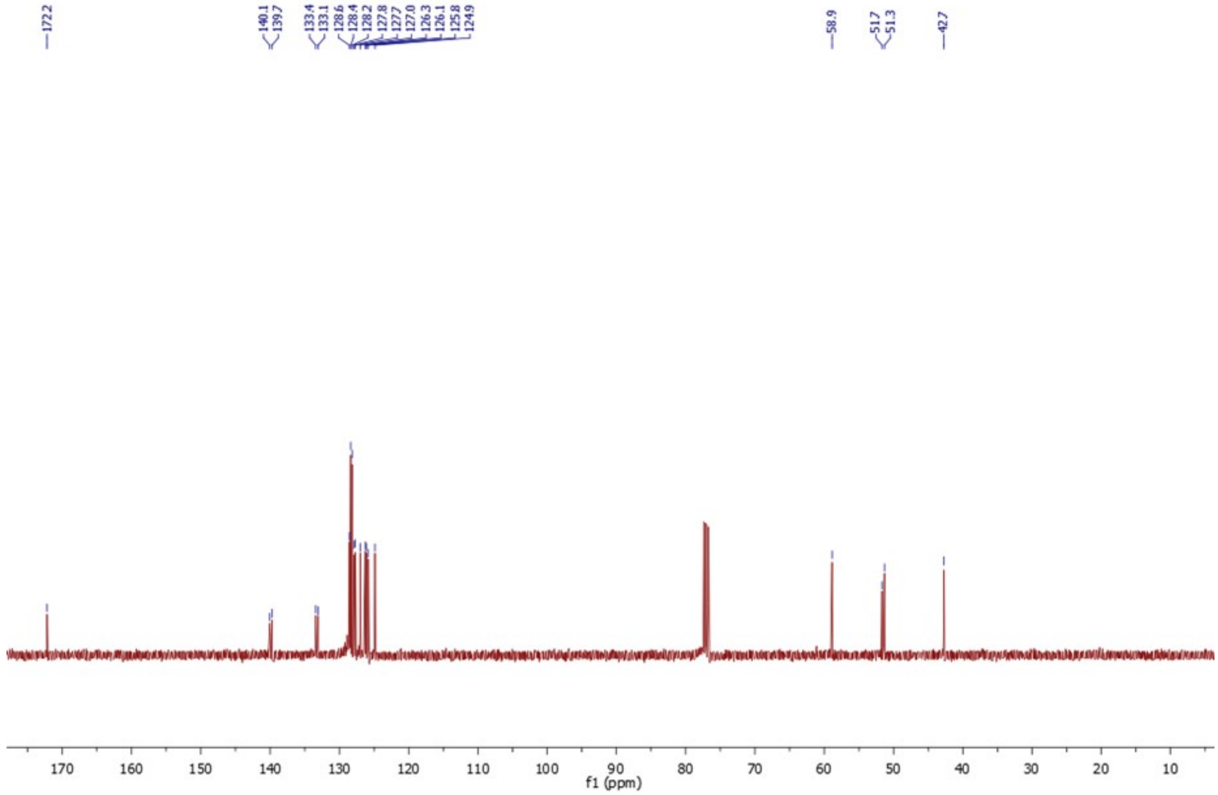
172.2
140.1
139.7
133.4
133.1
128.6
128.4
128.2
127.8
127.7
127.0
126.3
126.1
125.8
124.9
58.9
51.7
51.3
42.7
f1 (ppm)

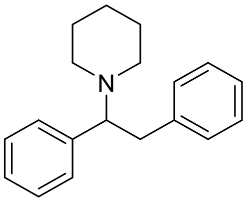

1-(1,2-diphenylethyl)piperidine (**5a**) was obtained following General procedure C.

^{1}H-NMR (400 MHz, $CDCl_3$): δ (ppm) 7.26 – 6.99 (m, 10H), 3.59 (dd, *J* = 9.2, 5.2 Hz, 1H), 3.30 (dd, *J* = 13.3, 5.2 Hz, 1H), 3.00 (dd, *J* = 13.3, 9.2 Hz, 1H), 2.50 – 2.35 (m, 4H), 1.63 – 1.49 (m, 4H), 1.41 – 1.31 (m, 2H).

^{13}C-NMR (101 MHz, $CDCl_3$): δ (ppm) 129.3, 128.9, 127.8, 127.6, 126.8, 125.6, 72.3, 51.4, 39.2, 26.3, 24.6.

HRMS (ESI-TOF): mass calculated for $C_{19}H_{23}N$, 265.1830; m/z found, 266.1901 $[M+H]^+$.

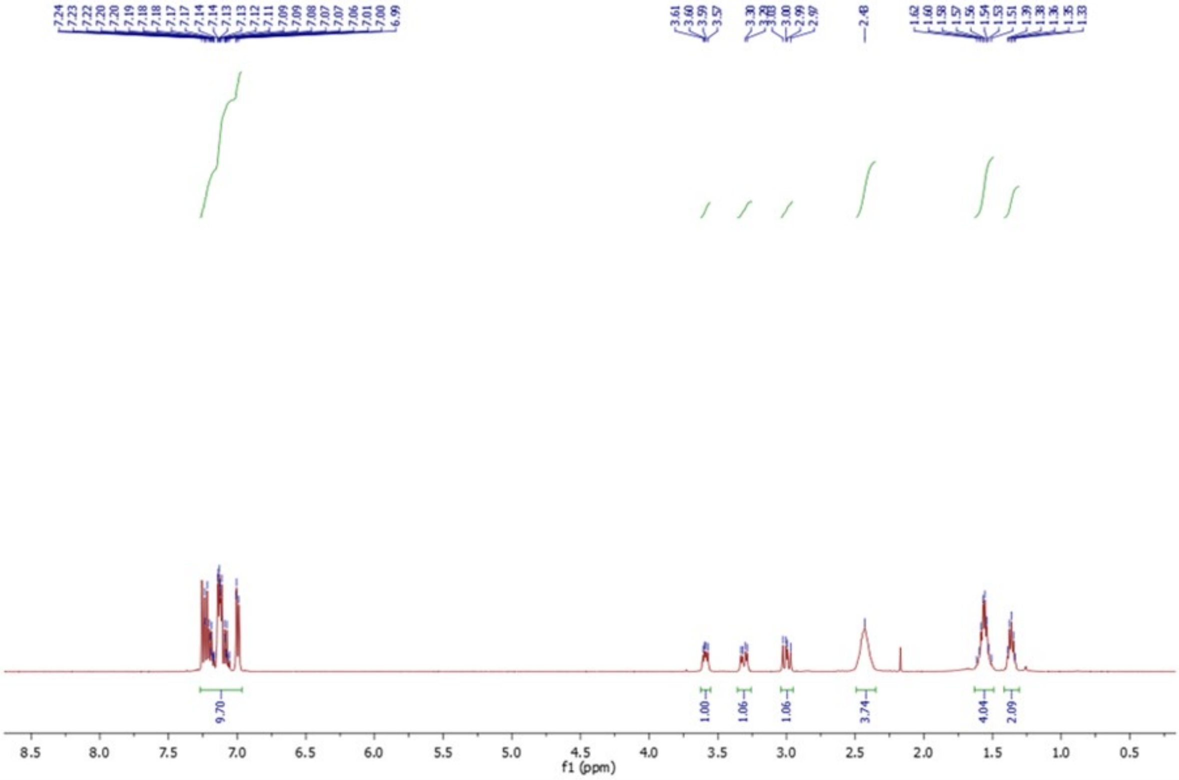

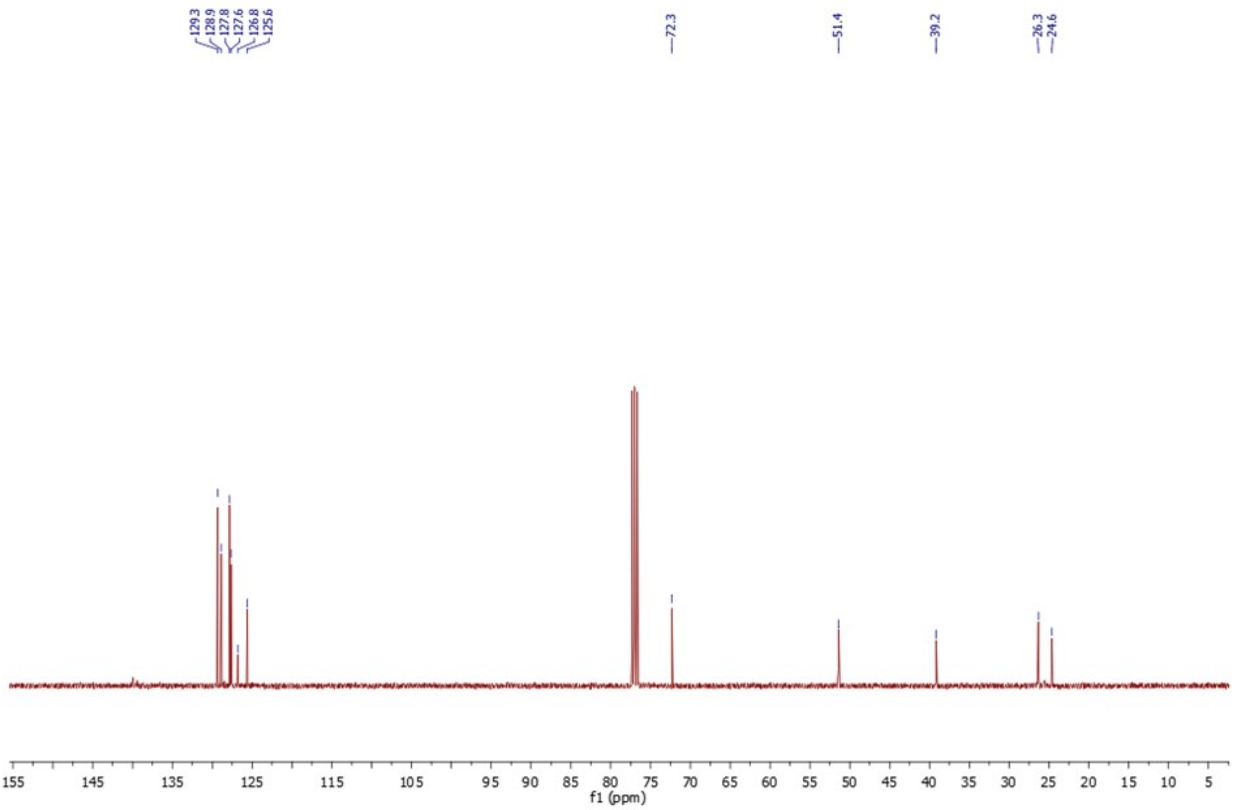

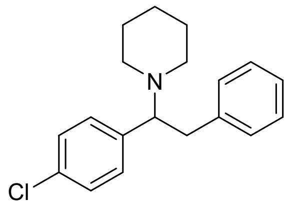

1-(1-(4-chlorophenyl)-2-phenylethyl)piperidine (**5b**) was obtained following General procedure C.

^{1}H-NMR (400 MHz, $CDCl_3$): δ (ppm) 7.21 (d, *J* = 8.4 Hz, 2H), 7.17 – 6.04 (m, 5H), 7 .01 – 6.95 (m, 2H), 3.68 – 3.54 (m, 1H), 3.42 – 3.27 (m, 1H), 2.95 (t, *J* = 10.6 Hz, 1H), 2.57 – 2.34 (m, 4H), 1.68 – 1.48 (m, 4H), 1.45 – 1.31 (m, 2H).

^{13}C-NMR (101 MHz, $CDCl_3$): δ (ppm) 139.2, 130.2, 129.3, 128.0, 127.9, 125.8, 71.6, 51.4 39.0, 26.1, 24.5.

HRMS (ESI-TOF): mass calculated for $C_{22}H_{23}N$, 299.1441; m/z found, 300.1519 $[M+H]^+$.

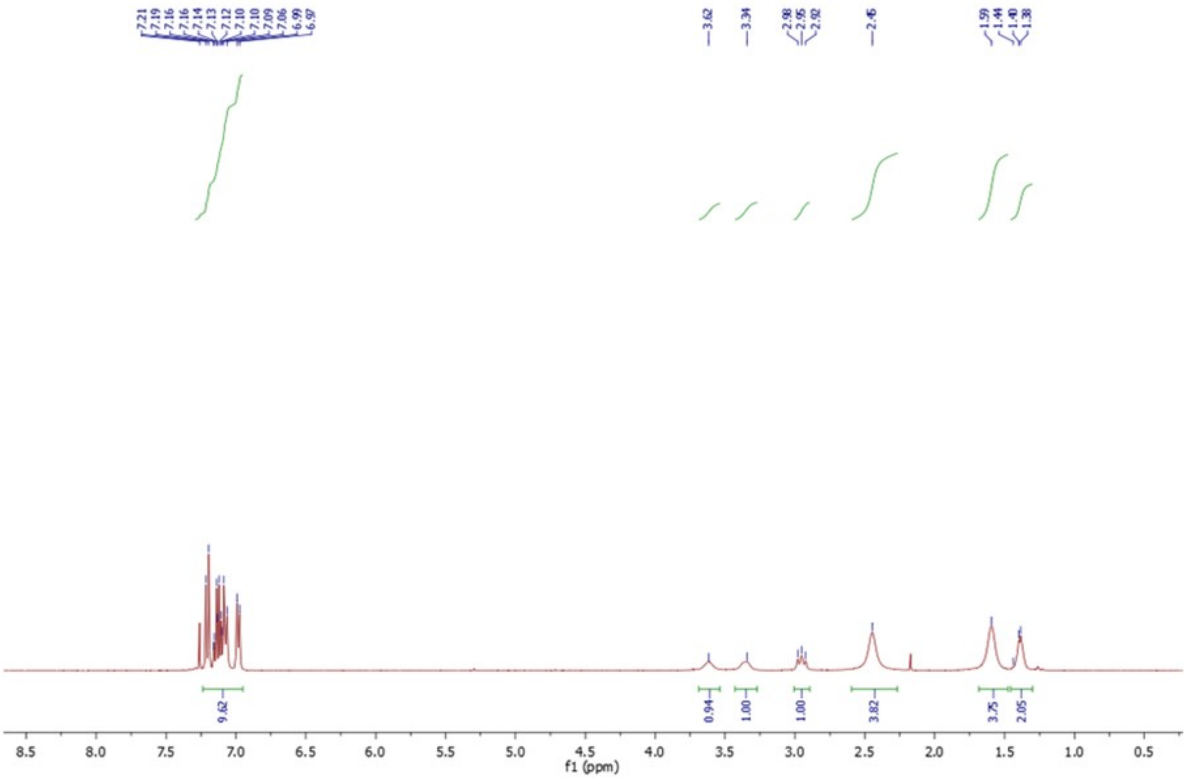
7.21
7.19
7.16
7.14
7.13
7.12
7.10
7.09
7.06
6.99
6.97
3.62
3.34
2.98
2.95
2.92
2.45
1.59
1.44
1.40
1.38
9.62
0.94
1.00
1.00
3.82
3.75
2.05
8.5 8.0 7.5 7.0 6.5 6.0 5.5 5.0 4.5 4.0 3.5 3.0 2.5 2.0 1.5 1.0 0.5
f1 (ppm)

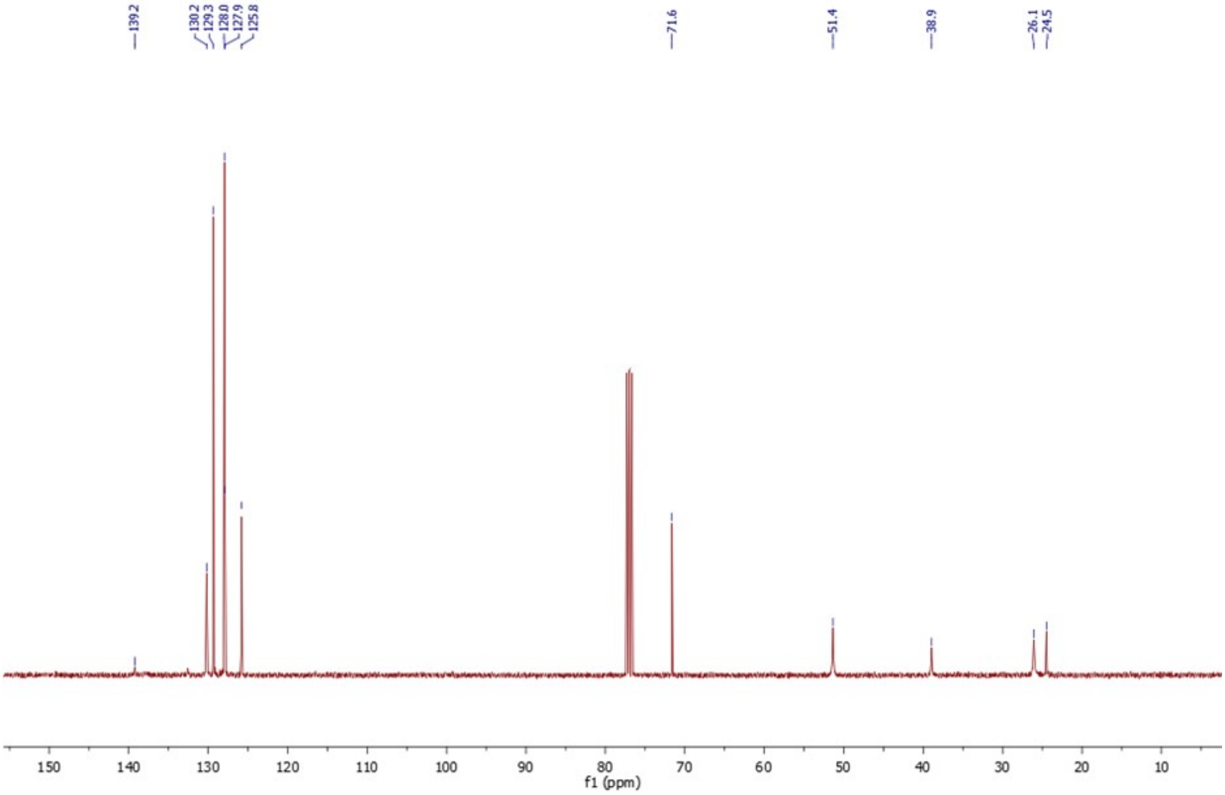
139.2
130.2
129.3
128.0
127.9
125.8
71.6
51.4
38.9
26.1
24.5
150 140 130 120 110 100 90 80 70 60 50 40 30 20 10
f1 (ppm)

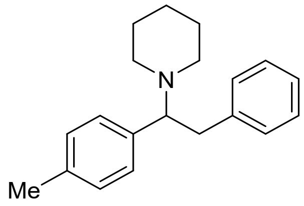

1-(2-phenyl-1-(p-tolyl)ethyl)piperidine (**5c**) was obtained following General procedure C.

^{1}H-NMR (400 MHz, $CDCl_3$): δ (ppm) 7.18 – 7.04 (m, 9H), 3.63 (dd, *J* = 9.4, 5.2 Hz, 1H), 3.31 (dd, *J* = 13.4, 5.2 Hz, 1H), 3.03 (dd, *J* = 13.4, 9.4 Hz, 1H), 2.49 – 2.43 (m, 4H), 2.32 (s, 3H), 1.65 – 1.54 (m, 4H), 1.41 – 1.35 (m, 2H).

^{13}C-NMR (101 MHz, $CDCl_3$): δ (ppm) 140.1, 136.4, 135.9, 129.4, 128.9, 128.4, 127.8, 125.6, 71.9, 51.3, 39.0, 26.3, 24.6, 21.1.

HRMS (ESI-TOF): mass calculated for $C_{20}H_{25}N$, 279.1987; m/z found, 280.2063 $[M+H]^+$.

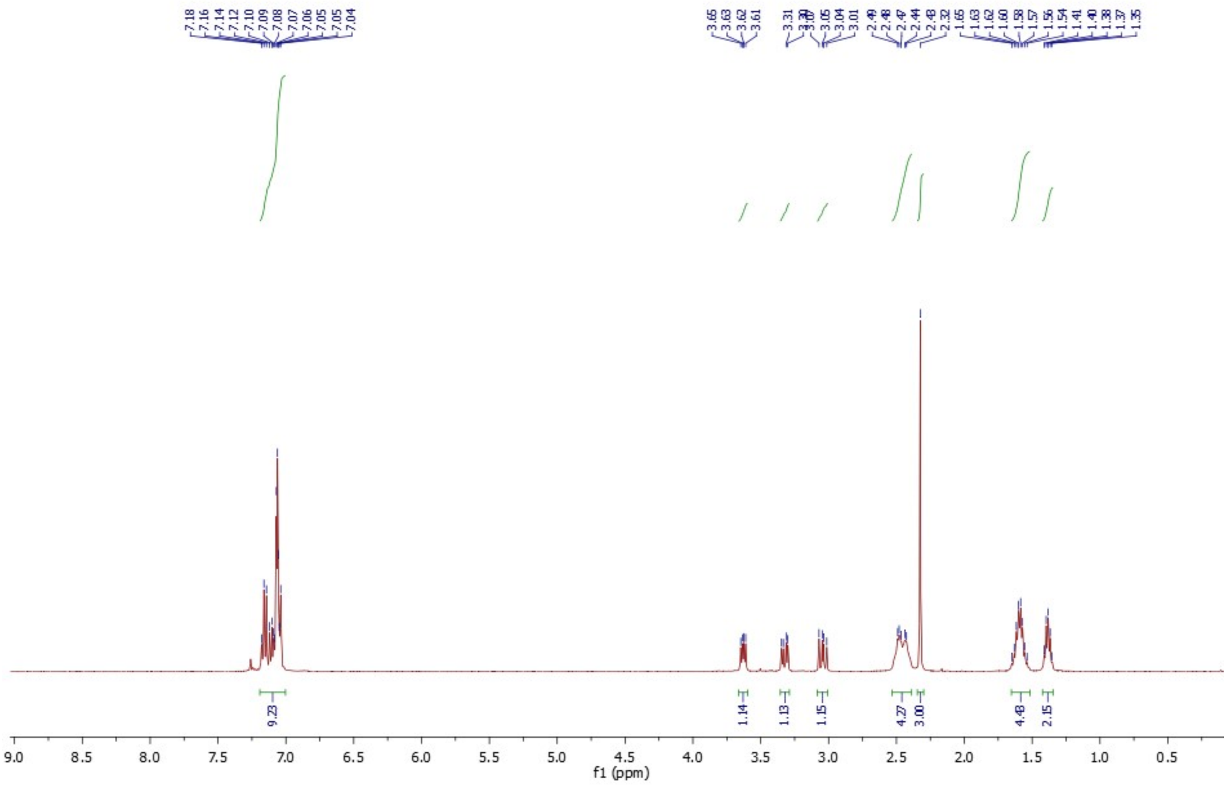

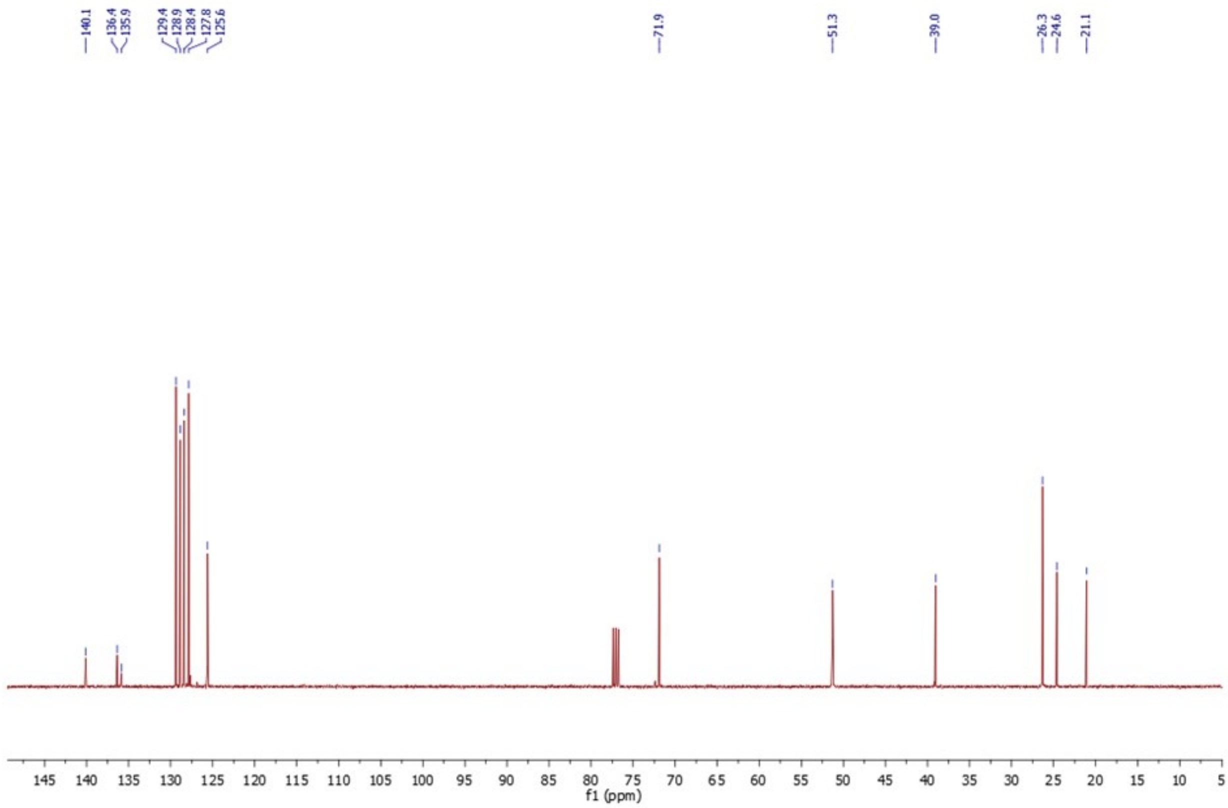

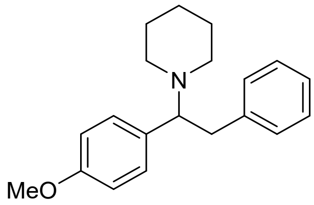

1-(1-(4-methoxyphenyl)-2-phenylethyl)piperidine (**5d**) was obtained following General procedure C.

^{1}H-NMR (400 MHz, $CDCl_3$): δ (ppm) 7.16 – 7.06 (m, 5H), 7.02 – 6.99 (m, 2H), 6.79 (d, *J* = 8.8 Hz, 2H), 3.77 (s, 3H), 3.73 – 3.65 (m, 1H), 3.45 – 3.37 (m, 1H), 3.06 (dd, *J* = 13.1, 10.9 Hz, 1H), 2.61 – 2.43 (m, 4H), 1.71 – 1.54 (m, 4H), 1.45 – 1.35 (m, 2H).

^{13}C-NMR (101 MHz, $CDCl_3$): δ (ppm) 158.8, 130.2, 129.3, 129.0, 2x 127.9, 127.8, 125.8, 113.3, 71.7, 55.1, 51.2, 38.7, 25.6, 24.2.

HRMS (ESI-TOF): mass calculated for $C_{20}H_{25}NO$, 295.1936; m/z found, 296.2012 $[M+H]^+$.

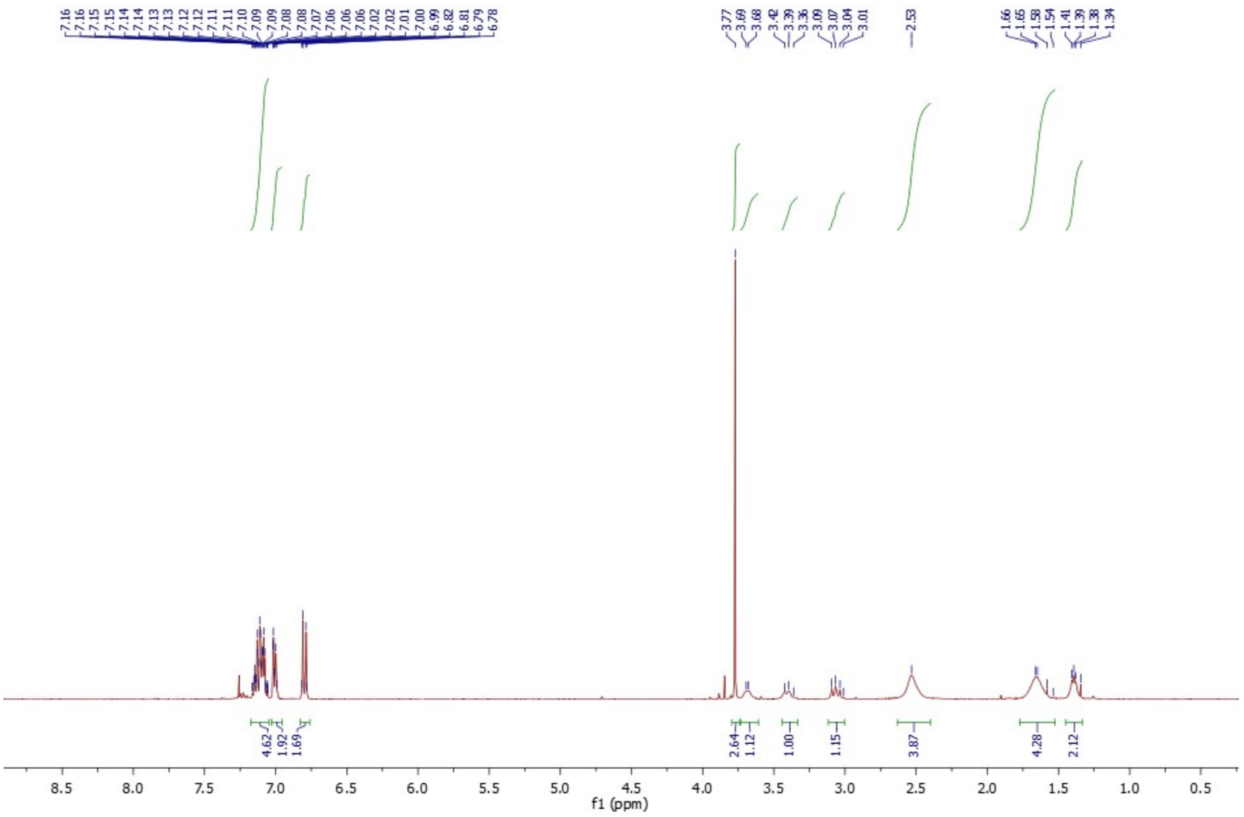
7.16 7.16 7.15 7.14 7.14 7.13 7.13 7.12 7.12 7.11 7.10 7.09 7.09 7.08 7.08 7.07 7.06 7.06 7.06 7.02 7.02 7.01 7.00 6.99 6.82 6.81 6.79 6.78
3.77 3.69 3.68 3.42 3.39 3.36 3.09 3.07 3.04 3.01
2.53
1.66 1.65 1.58 1.54 1.41 1.39 1.38 1.34
4.62 1.92 1.69 2.64 1.12 1.00 1.15 3.87 4.28 2.12
f1 (ppm)

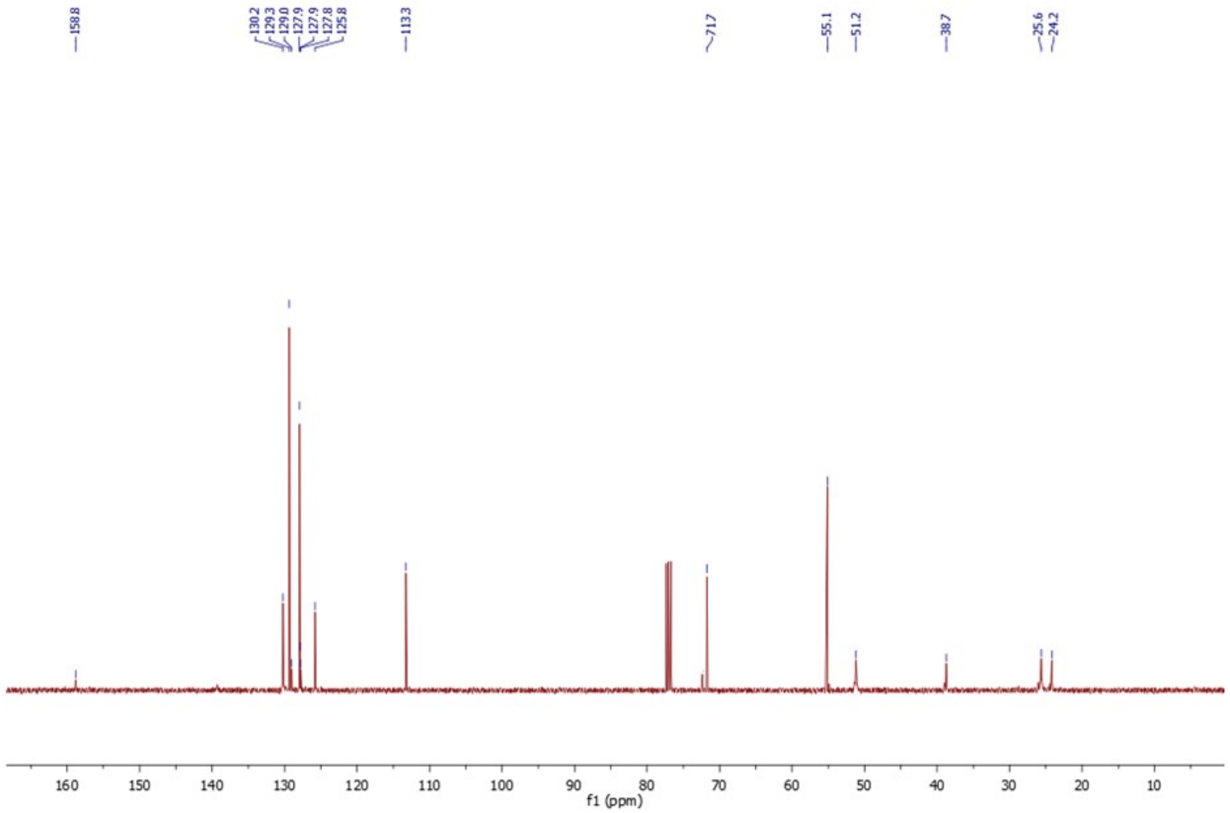
158.8
130.2 129.3 129.0 127.9 127.9 127.8 125.8
113.3
71.7
55.1
51.2
38.7
25.6 24.2
f1 (ppm)

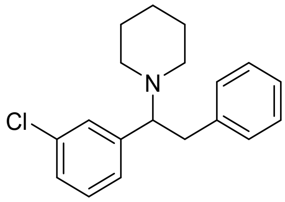

1-(1-(3-chlorophenyl)-2-phenylethyl)piperidine (**5e**) was obtained following General procedure C.

^{1}H-NMR (400 MHz, CDCl$_3$): δ (ppm) 7.19 – 6.98 (m, 9H), 3.76 – 3.66 (m, 1H), 3.46 – 3.39 (m, 1H), 3.02 (t, *J* = 10.6 Hz, 1H), 2.65 – 2.49 (m, 4H), 1.75 – 1.5 (m, 4H), 1.47 – 1.37 (m, 2H).

^{13}C-NMR (101 MHz, CDCl$_3$): δ (ppm) 133.9, 2x 129.3, 129.2, 129.0, 128.1, 127.3, 126.0, 71.9, 51.3, 38.5, 25.6, 24.1.

HRMS (ESI-TOF): mass calculated for $C_{19}H_{22}NCl$, 299.1441; m/z found, 300.1512 [M+H]$^{+}$.

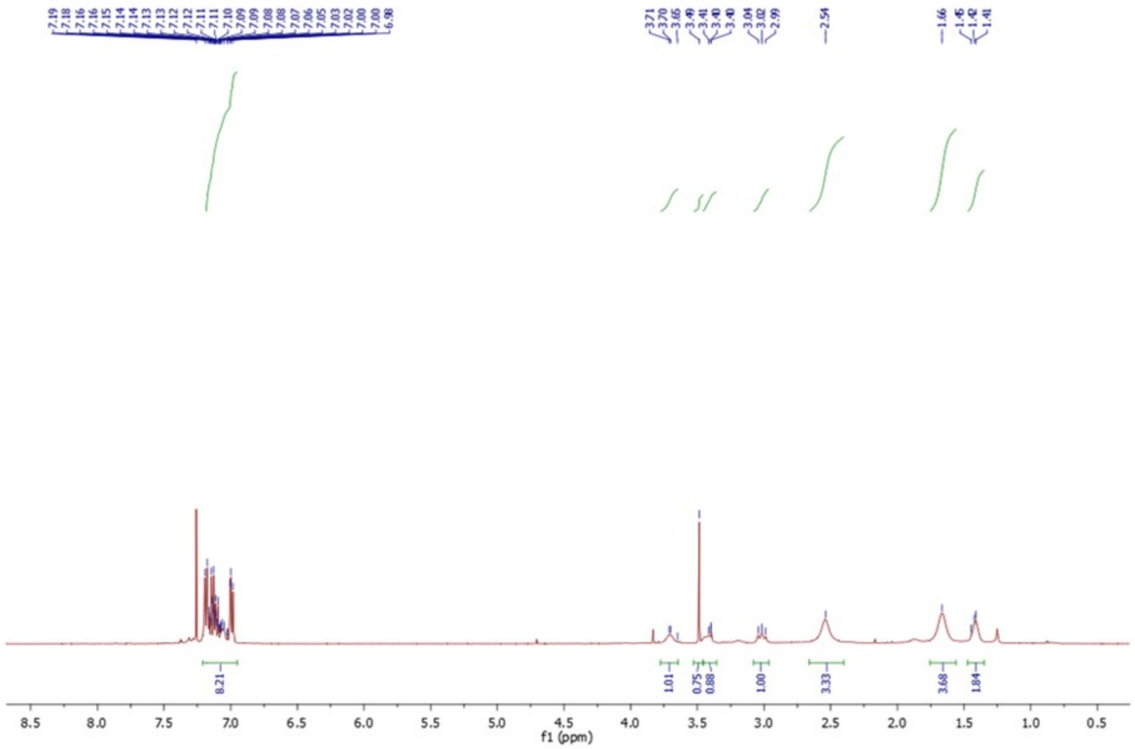

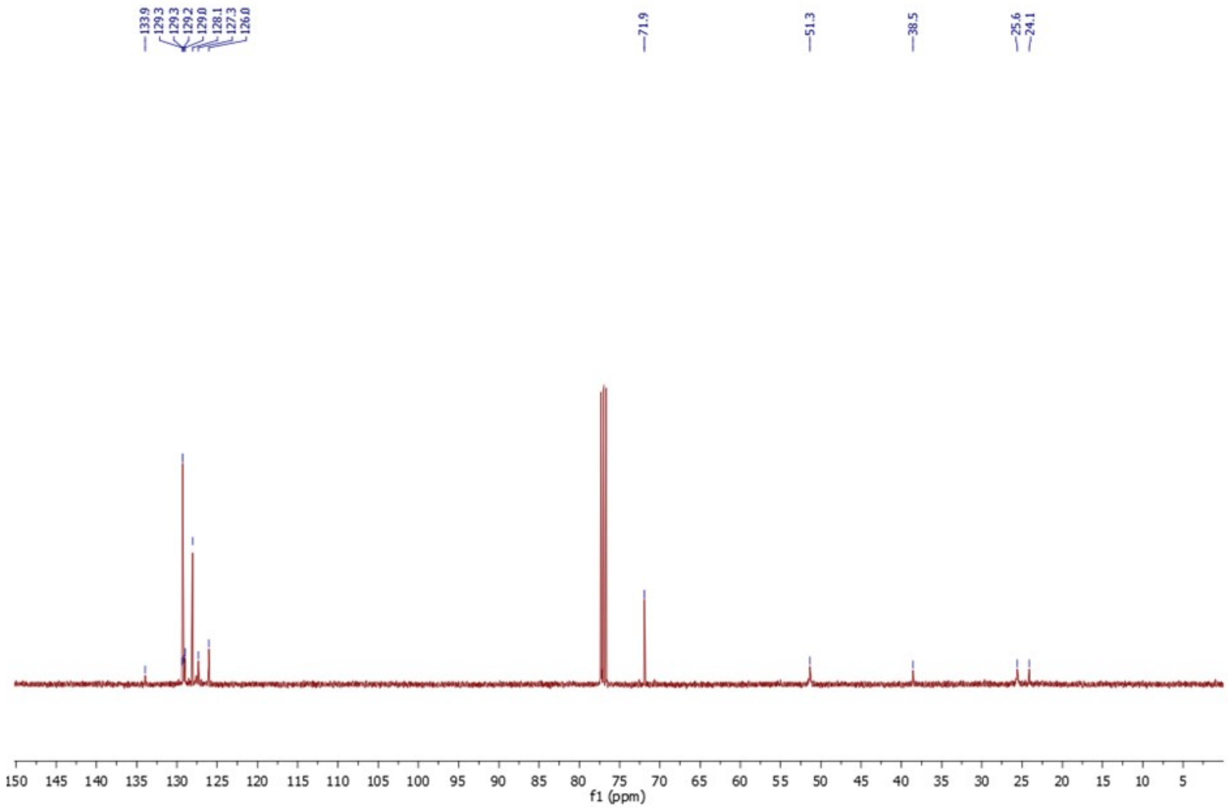

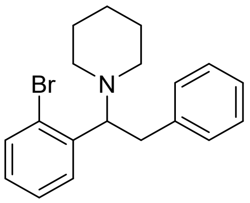

1-(1-(2-bromophenyl)-2-phenylethyl)piperidine (**5f**) was obtained following General procedure C.

^{1}H-NMR (400 MHz, $CDCl_3$): δ (ppm) 7.50 – 7.43 (m,1H), 7.39 (dd, *J* = 8.1, 1.0 Hz, 1H), 7.16 – 7.06 (m, 4H), 7.05 – 6.97 (m, 3H), 4.35 – 4.25 (m, 1H), 3.33 – 3.23 (m, 1H), 2.98 – 2.87 (m, 1H), 2.67 -2.54 (m, 2H), 2.50 – 2.36 (m, 2H), 1.61 – 1.49 (m, 4H), 1.44 – 1.36 (m, 2H).

^{13}C-NMR (101 MHz, $CDCl_3$): δ (ppm) 132.6, 129.4, 127.8, 126.1, 68.6, 51.5, 39.2, 26.3, 24.6.

HRMS (ESI-TOF): mass calculated for $C_{19}H_{22}NBr$, 343.0936; m/z found, 344.1011 $[M+H]^+$.

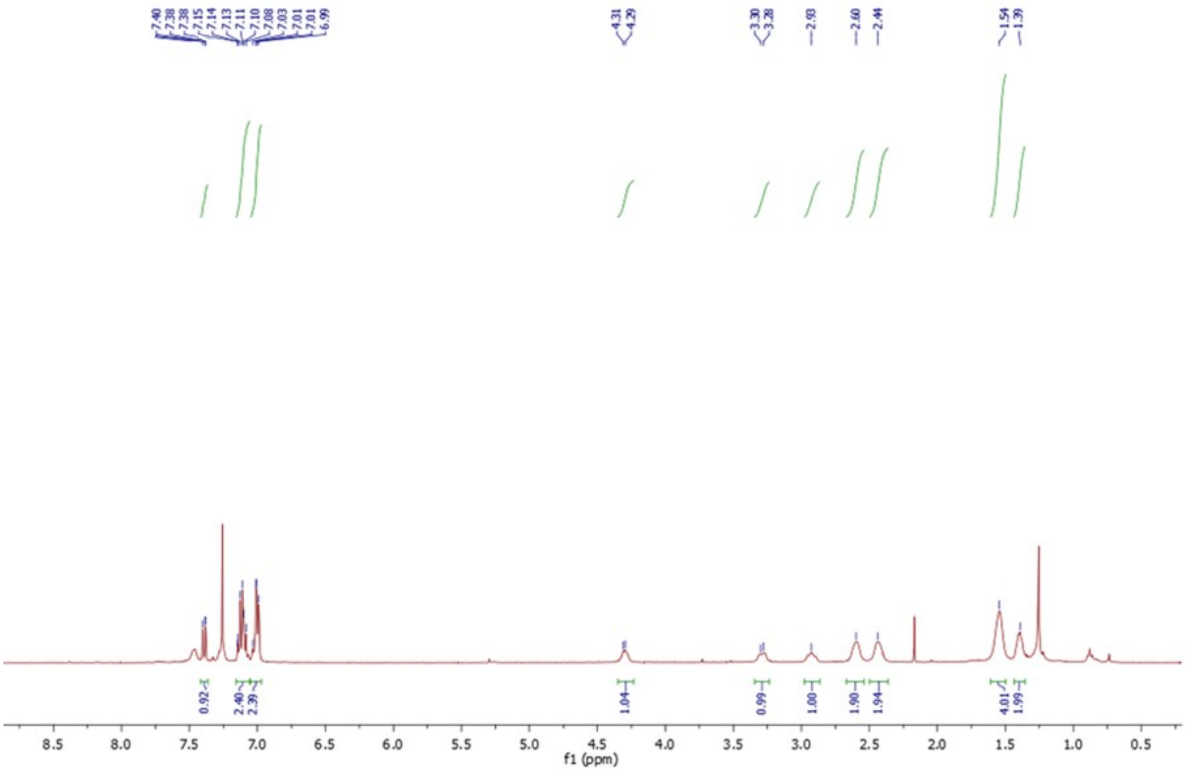

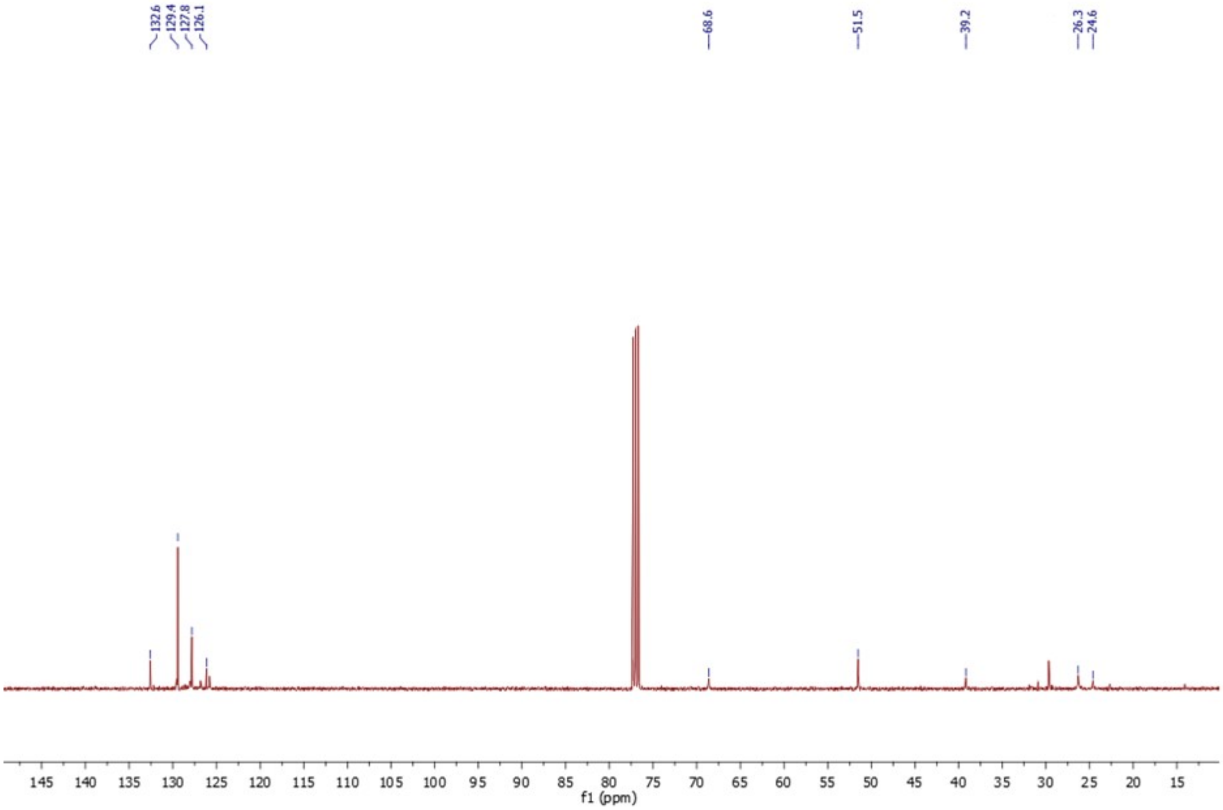

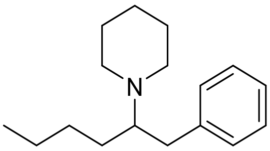

1-(1-phenylhexan-2-yl)piperidine (**5g**) was obtained following the General procedure C.

^{1}H-NMR (400 MHz, CDCl$_3$): δ (ppm) 7.30 – 7.22 (m, 2H), 7.20 – 7.14 (m, 3H), 3.02 – 2.93 (m, 1H), 2.69 – 2.55 (m, 3H), 2.54 – 2.42 (m, 2H), 2.40 – 2.31 (m, 1H), 1.64 – 1.51 (m, 4H), 1.48 – 1.40 (m, 2H), 1.37 – 1.06 (m, 6H), 0.81 (t, *J* = 7.1 Hz, 3H).

^{13}C-NMR (101 MHz, CDCl$_3$): δ (ppm) 129.2, 128.1, 125.5, 66.9, 49.6, 35.8, 30.2, 29.3, 26.5, 25.0, 22.7, 14.1.

HRMS (ESI-TOF): mass calculated for $C_{17}H_{27}N$, 245.2143; m/z found, 246.2221 $[M+H]^+$.

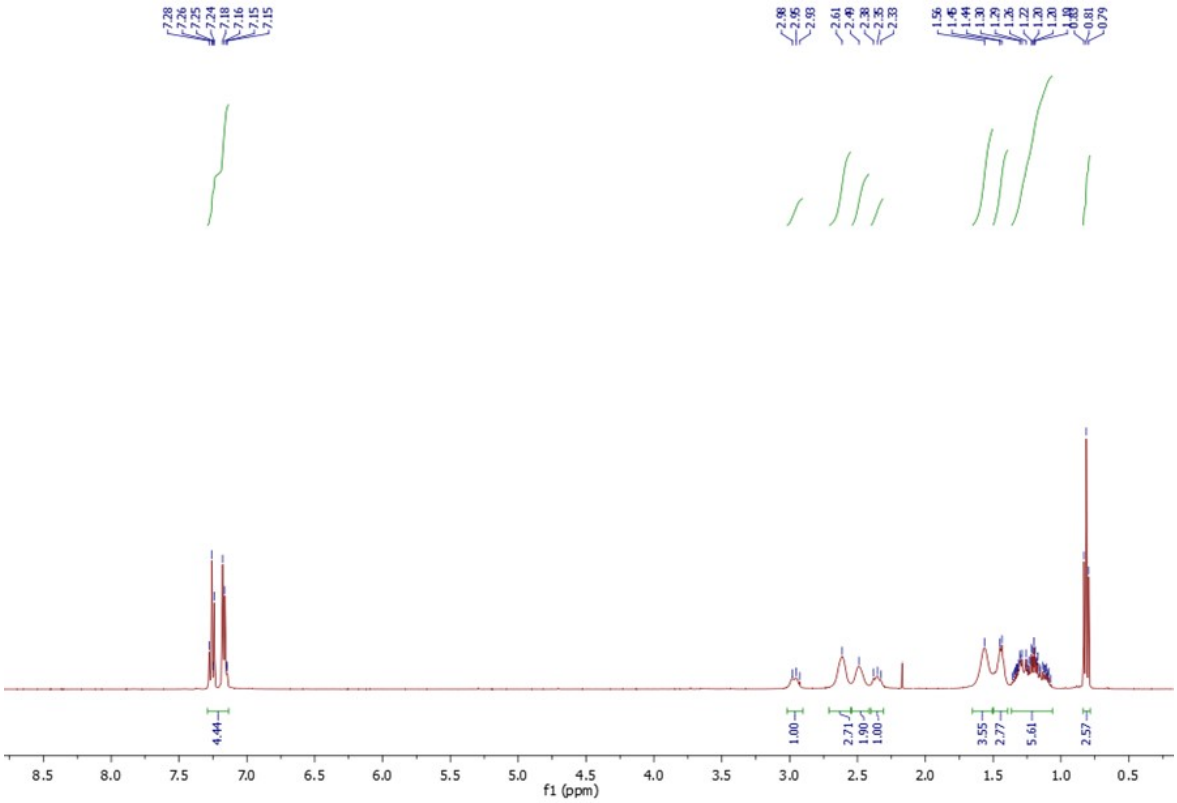

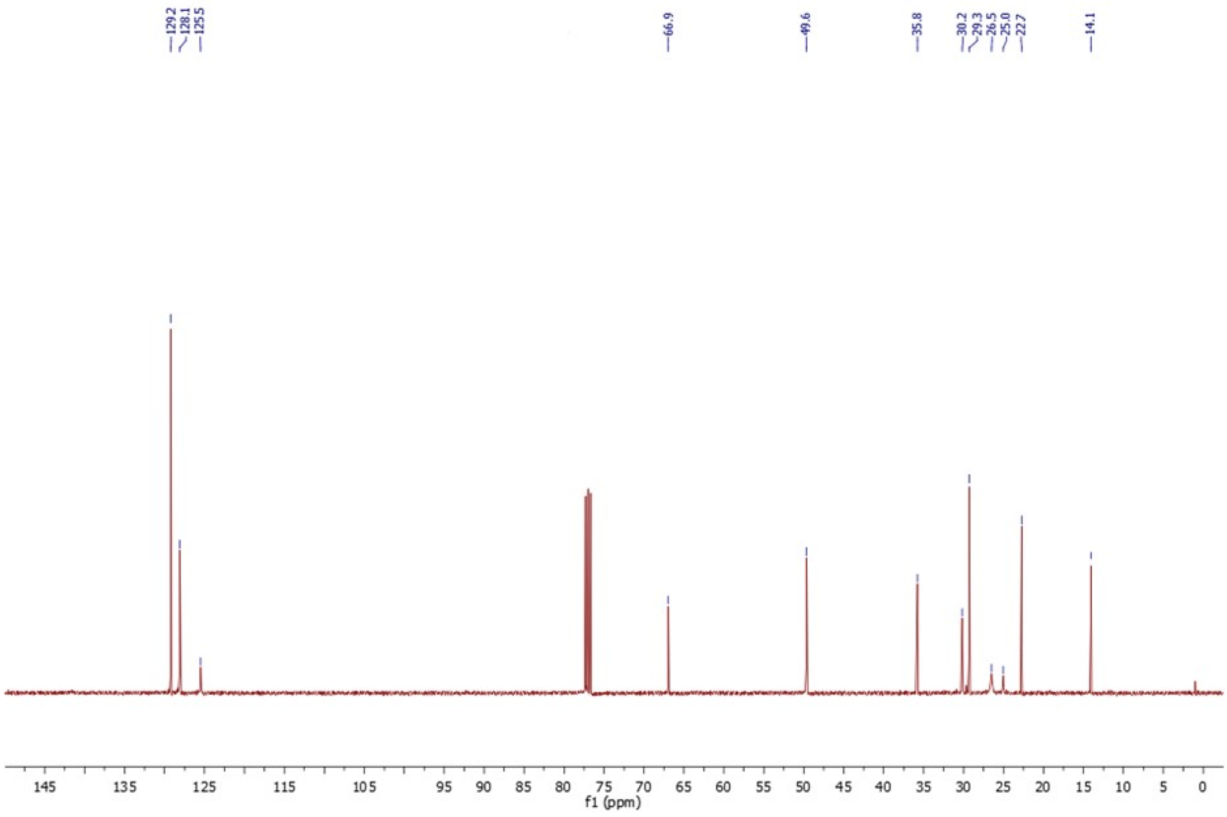
129.2
128.1
125.5
66.9
49.6
35.8
30.2
29.3
26.5
25.0
22.7
14.1
145
135
125
115
105
95
90
85
80
75
70
65
60
55
50
45
40
35
30
25
20
15
10
5
0
f1 (ppm)

Computational studies

Cartesian coordinates for benzylzinc bromide

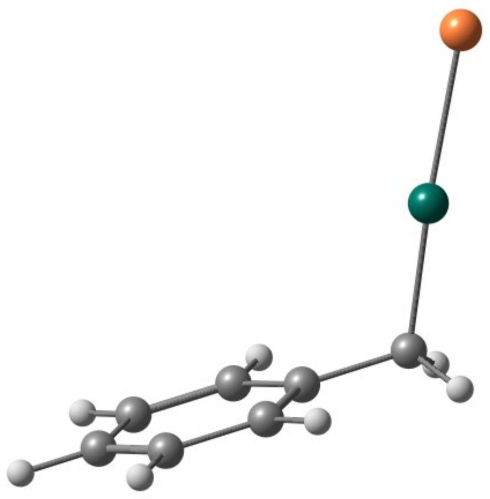

C	-0.014397	0.038463	-1.444078
C	-1.132729	-0.416285	-0.750255
C	1.124441	0.396778	-0.727268
H	-2.027394	-0.706045	-1.293032
H	2.007755	0.748141	-1.251948
C	-1.112033	-0.508099	0.636896
C	1.142134	0.303784	0.659727
H	-1.992241	-0.868377	1.162853
H	2.039976	0.585039	1.203778
C	0.024811	-0.149902	1.375329
H	-0.028611	0.108578	-2.526536
C	0.043336	-0.243296	2.860197
H	1.049187	-0.451327	3.235866
H	-0.626770	-1.028400	3.220787
Zn	-0.521666	1.425618	3.767166
Br	-0.988539	3.361466	4.973639

Cartesian coordinates $ZnC_{19}NH_{24}OBr$

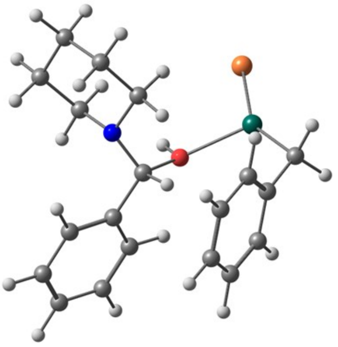

C	-0.928834000	-2.855850000	-0.760406000
C	-0.116511000	-1.725227000	-0.783828000
C	-1.820701000	-3.036362000	0.290006000
H	0.581543000	-1.573573000	-1.600671000
H	-2.460606000	-3.912251000	0.316287000
C	-0.194146000	-0.787384000	0.238174000
C	-1.902091000	-2.094220000	1.312331000
H	0.441082000	0.091485000	0.225661000
H	-2.609469000	-2.238464000	2.120673000
C	-1.083603000	-0.965902000	1.300383000
H	-0.866897000	-3.589579000	-1.557212000
C	-1.192687000	0.110952000	2.364018000
H	-1.851072000	0.905798000	2.001248000
N	0.057496000	0.691781000	2.698773000
C	-0.032088000	1.958914000	3.416547000
H	-0.358340000	1.800131000	4.460564000
H	-0.792207000	2.575144000	2.927458000
C	1.313206000	2.670381000	3.419016000
8	-1.940931000	-0.402745000	3.520197000
H	-1.440789000	-1.104714000	3.954758000
C	-3.636315000	2.581074000	-0.115268000
C	-3.214929000	3.487530000	0.853764000
C	-4.365064000	1.461716000	0.281064000
H	-3.405681000	2.746257000	-1.162326000
H	-2.654007000	4.370876000	0.563347000
C	-3.510784000	3.273491000	2.196008000
H	-4.707905000	0.747181000	-0.461260000
C	-4.656977000	1.249232000	1.622936000
H	-3.177741000	3.993677000	2.938849000
C	-4.232162000	2.145785000	2.616399000
H	-5.221775000	0.367464000	1.914507000
C	-4.511164000	1.895546000	4.053030000
Zn	-3.211635000	0.626900000	4.891732000
H	-5.492098000	1.430583000	4.192796000
H	-4.486969000	2.820850000	4.634666000
Br	-2.360753000	-0.489949000	6.827801000
C	2.400160000	1.775562000	4.004625000
C	2.424002000	0.431132000	3.285719000
C	1.043129000	-0.209377000	3.289844000
H	1.572965000	2.942485000	2.388891000
H	2.197121000	1.610967000	5.071362000
H	3.138035000	-0.250325000	3.758753000
H	1.053778000	-1.142182000	2.719642000
H	1.227020000	3.600881000	3.989141000
H	3.377555000	2.263898000	3.941115000
H	2.745784000	0.571418000	2.246811000
H	0.772629000	-0.459521000	4.332653000

Trisubstituted Hemiaminal

Cartesian coordinatesSN1 reaction profile

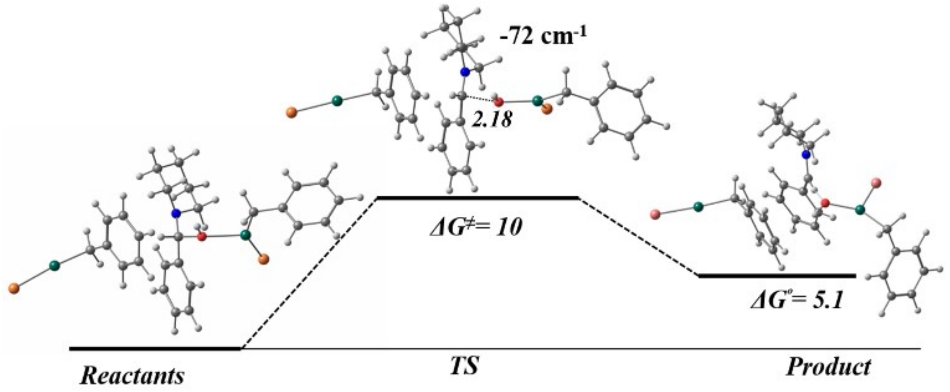

Reactants

C	3.045242000	3.966397000	3.274843000
C	2.331434000	4.138191000	2.092798000
C	2.862541000	2.808148000	4.022534000
H	2.467827000	5.037017000	1.500250000
H	3.415744000	2.663124000	4.944742000
C	1.441493000	3.159647000	1.664275000
C	1.970334000	1.830228000	3.594807000
H	0.889754000	3.288223000	0.739632000
H	1.834113000	0.929879000	4.181497000
C	1.245533000	1.999642000	2.415414000
H	3.740131000	4.729460000	3.609699000
C	0.353178000	0.898207000	1.884589000
H	0.974241000	0.212879000	1.296452000
C	2.191753000	-2.884666000	0.989860000
C	1.682048000	-2.713951000	-0.294554000
C	3.085644000	-1.941427000	1.491846000
H	0.982065000	-3.437573000	-0.701343000
H	3.491837000	-2.057049000	2.492182000
C	2.057350000	-1.616214000	-1.061655000
C	3.459970000	-0.846047000	0.722182000
H	1.644206000	-1.494122000	-2.059483000
H	4.153901000	-0.116546000	1.131354000
C	2.952562000	-0.654545000	-0.571684000
H	1.898354000	-3.739543000	1.589920000
C	3.341716000	0.526624000	-1.386930000
H	3.510737000	1.405762000	-0.758143000
H	2.572710000	0.775079000	-2.123263000
Zn	5.030394000	0.288755000	-2.398972000
Br	7.114030000	0.196961000	-3.453164000
N	-0.688787000	1.350820000	1.039925000
C	-1.285044000	0.324540000	0.192595000
H	-1.960626000	-0.327366000	0.776824000
O	-0.103064000	0.093663000	3.035989000
H	-0.214255000	-0.815537000	2.740419000
C	-4.832043000	-2.052651000	8.223500000
C	-3.478113000	-2.360758000	8.330778000
C	-5.319806000	-1.581000000	7.007247000
H	-5.495838000	-2.180212000	9.072031000
H	-3.079567000	-2.734420000	9.269340000
C	-2.628765000	-2.196626000	7.242517000
H	-6.373442000	-1.339765000	6.902257000
C	-4.467819000	-1.417860000	5.920946000
H	-1.574910000	-2.442553000	7.344943000
C	-3.100001000	-1.718815000	6.010015000
H	-4.866576000	-1.048497000	4.979567000
C	-2.186698000	-1.518648000	4.856236000
Zn	-1.377303000	0.321863000	4.794992000
H	-2.713219000	-1.650534000	3.905670000
H	-1.350855000	-2.224902000	4.885334000
Br	-1.270079000	2.455006000	5.806754000
C	-1.697088000	2.221883000	1.633546000
C	-2.485151000	2.936542000	0.544463000
C	-3.121950000	1.929135000	-0.407526000
C	-2.070711000	0.962964000	-0.944576000
H	-0.482749000	-0.305915000	-0.203567000
H	-2.537537000	0.177350000	-1.547384000
H	-1.371927000	1.501634000	-1.596011000
H	-3.891334000	1.361193000	0.132825000
H	-3.629344000	2.441864000	-1.230717000

H	-1.204513000	2.939653000	2.293152000
H	-2.401924000	1.637998000	2.252159000
H	-1.808377000	3.597386000	-0.010679000
H	-3.249719000	3.568516000	1.007524000

TS

C	2.964348000	3.269721000	3.798289000
C	2.384234000	3.661233000	2.596951000
C	2.674326000	2.015924000	4.329290000
H	2.616408000	4.629611000	2.167375000
H	3.127715000	1.703979000	5.264056000
C	1.508338000	2.809254000	1.935471000
C	1.805257000	1.158286000	3.669262000
H	1.088339000	3.111987000	0.982716000
H	1.554199000	0.186912000	4.075045000
C	1.199606000	1.555090000	2.474271000
H	3.645093000	3.936857000	4.316277000
C	0.377607000	0.612973000	1.690067000
H	0.854714000	-0.314245000	1.392095000
C	2.533710000	-2.680441000	0.210690000
C	1.928670000	-2.347395000	-0.998513000
C	3.325023000	-1.730813000	0.853426000
H	1.308172000	-3.074652000	-1.513419000
H	3.803882000	-1.972747000	1.797375000
C	2.107932000	-1.083792000	-1.551541000
C	3.503243000	-0.469294000	0.297356000
H	1.622904000	-0.837994000	-2.492563000
H	4.118673000	0.261514000	0.815154000
C	2.895148000	-0.112879000	-0.915597000
H	2.392216000	-3.664455000	0.644834000
C	3.070526000	1.243605000	-1.497850000
H	3.152423000	2.002945000	-0.714423000
H	2.231926000	1.511344000	-2.146721000
Zn	4.702133000	1.462176000	-2.595584000
Br	6.625248000	1.860016000	-3.842160000
N	-0.656249000	0.962856000	0.979570000
C	-1.241173000	0.025785000	0.016990000
H	-2.163216000	-0.371673000	0.458856000
O	-0.431493000	-0.635346000	3.281840000
H	-0.328190000	-1.536009000	2.961549000
C	-4.803964000	-1.095813000	9.131967000
C	-3.501041000	-1.589003000	9.125857000
C	-5.469339000	-0.946232000	7.917533000
H	-5.291876000	-0.835328000	10.065304000
H	-2.965870000	-1.717852000	10.062233000
C	-2.877384000	-1.922232000	7.929301000
H	-6.487252000	-0.567600000	7.900195000
C	-4.842914000	-1.280987000	6.722508000
H	-1.860104000	-2.305448000	7.945898000
C	-3.528836000	-1.776462000	6.693072000
H	-5.379924000	-1.157785000	5.785395000
C	-2.851313000	-2.105577000	5.418122000
Zn	-1.826990000	-0.556739000	4.617377000
H	-3.571654000	-2.411363000	4.652266000
H	-2.128568000	-2.917176000	5.548993000
Br	-2.291782000	1.797402000	5.102557000
C	-1.500012000	2.130514000	1.252738000
C	-1.809082000	2.884781000	-0.032085000
C	-2.426142000	1.957111000	-1.073915000
C	-1.541540000	0.734876000	-1.296135000
H	-0.542741000	-0.799870000	-0.125309000
H	-2.021846000	0.021965000	-1.972198000
H	-0.595697000	1.035482000	-1.761650000
H	-3.415886000	1.632368000	-0.728727000
H	-2.578339000	2.491197000	-2.015822000
H	-1.016702000	2.756401000	1.999060000
H	-2.426288000	1.747752000	1.698400000
H	-0.887701000	3.329319000	-0.426411000
H	-2.486422000	3.709235000	0.206901000

Products

C	2.299779000	3.882691000	4.164226000
C	2.309439000	4.147716000	2.797211000
C	1.403585000	2.956978000	4.690441000
H	3.027627000	4.846660000	2.383586000
H	1.404582000	2.738862000	5.752391000
C	1.406103000	3.514749000	1.956492000
C	0.518340000	2.297639000	3.851324000
H	1.452943000	3.704499000	0.890918000

H	-0.176982000	1.565869000	4.249057000
C	0.487370000	2.594228000	2.480806000
H	3.003142000	4.387899000	4.817280000
C	-0.462919000	1.831036000	1.686968000
H	-0.668699000	0.791040000	1.994263000
C	1.434386000	-3.273382000	1.456725000
C	1.178159000	-2.978683000	0.119418000
C	2.170491000	-2.368127000	2.218181000
H	0.603651000	-3.671361000	-0.488081000
H	2.376659000	-2.579940000	3.262972000
C	1.648904000	-1.798467000	-0.444288000
C	2.638176000	-1.188163000	1.651738000
H	1.436984000	-1.582273000	-1.488157000
H	3.204851000	-0.489510000	2.261600000
C	2.387428000	-0.873991000	0.308281000
H	1.068950000	-4.195008000	1.897498000
C	2.884130000	0.392032000	-0.293192000
H	2.950900000	1.189102000	0.452489000
H	2.234336000	0.733200000	-1.104195000
Zn	4.698469000	0.248644000	-1.087022000
Br	6.792959000	0.172713000	-2.119734000
N	-1.118811000	2.221385000	0.646483000
C	-1.967032000	1.264491000	-0.084470000
H	-2.992232000	1.646453000	-0.034052000
O	-1.276867000	-0.970960000	2.222089000
H	-0.685635000	-1.722301000	2.121597000
C	-2.051491000	-2.205518000	8.813140000
C	-1.422728000	-3.046102000	7.896828000
C	-3.166042000	-1.479266000	8.400716000
H	-1.682294000	-2.121696000	9.829860000
H	-0.555904000	-3.626404000	8.199690000
C	-1.897670000	-3.153341000	6.595219000
H	-3.674818000	-0.823030000	9.100939000
C	-3.640347000	-1.590078000	7.098431000
H	-1.393448000	-3.815094000	5.895256000
C	-3.018755000	-2.427626000	6.157243000
H	-4.513139000	-1.016868000	6.796943000
C	-3.505902000	-2.524031000	4.760260000
Zn	-2.593433000	-1.194614000	3.556364000
H	-4.578049000	-2.315689000	4.697655000
H	-3.321361000	-3.520014000	4.345881000
Br	-3.408177000	1.217162000	3.801065000
C	-1.143409000	3.571022000	0.072985000
C	-0.641365000	3.535971000	-1.365628000
C	-1.457784000	2.548535000	-2.195107000
C	-1.495440000	1.175555000	-1.531374000
H	-1.913630000	0.307738000	0.438805000
H	-2.168501000	0.501357000	-2.067984000
H	-0.499664000	0.717794000	-1.550650000
H	-2.481010000	2.929002000	-2.302653000
H	-1.041523000	2.469846000	-3.202994000
H	-0.578891000	4.249344000	0.707169000
H	-2.192920000	3.883959000	0.093501000
H	0.417804000	3.253435000	-1.375103000
H	-0.710231000	4.545933000	-1.778042000

Integrated QTAIM Charges for reactants, iminium salt and benzylzinc bromide

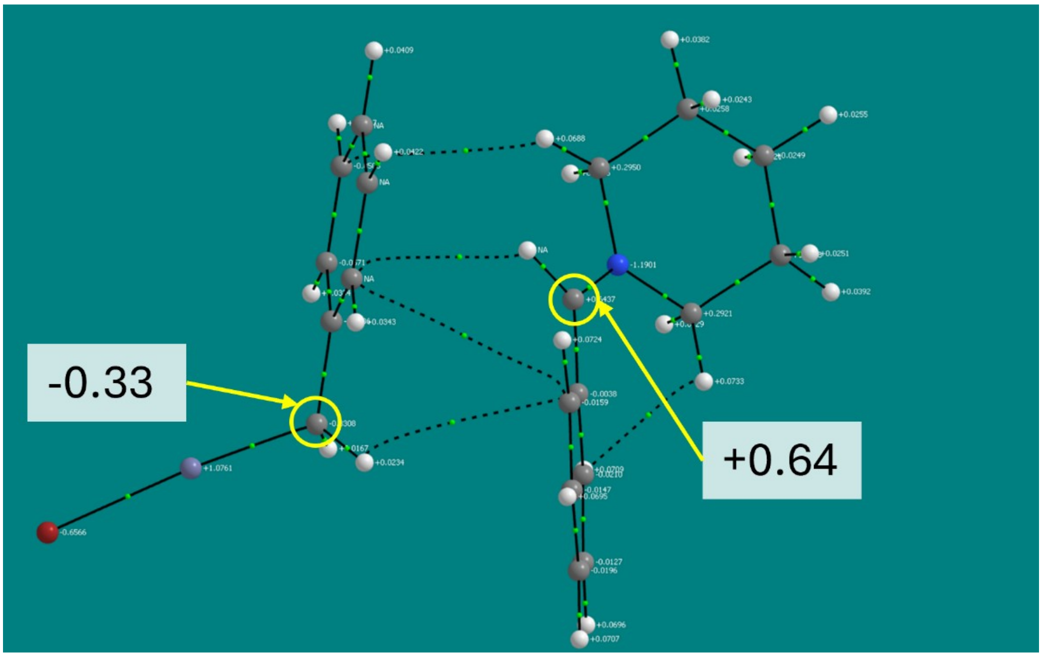

Cartesian coordinates, Mannich reaction profile

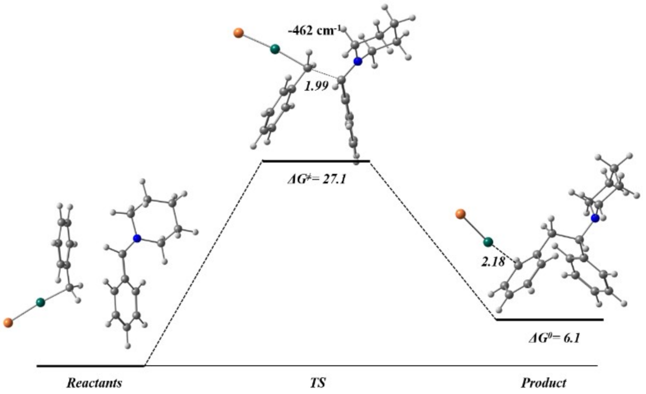

Reactants

C	-0.680658000	3.917279000	0.781363000
C	-1.148532000	3.565292000	-0.482139000
C	-0.840781000	3.047032000	1.855870000
H	-0.994162000	4.229869000	-1.324405000
H	-0.462051000	3.313412000	2.835929000
C	-1.807621000	2.360828000	-0.672011000
C	-1.468758000	1.826015000	1.667408000
H	-2.133842000	2.083738000	-1.667255000
H	-1.572870000	1.133801000	2.495964000
C	-1.989317000	1.487141000	0.409915000
H	-0.174130000	4.865510000	0.924360000
C	-2.618160000	0.183512000	0.302082000
H	-2.184705000	-0.614031000	0.905079000
C	-5.523229000	-1.712101000	-0.016138000
C	-6.432459000	-0.810589000	-0.839949000
C	-5.945765000	0.628723000	-0.760159000
C	-4.486182000	0.767716000	-1.180053000
C	-4.074668000	-1.565751000	-0.453887000
H	-5.800822000	-2.763667000	-0.124986000
H	-5.604803000	-1.463786000	1.047916000
H	-7.462357000	-0.879438000	-0.480919000
H	-6.434368000	-1.144255000	-1.884672000
H	-6.533859000	1.284096000	-1.407785000
H	-6.054267000	1.005295000	0.262902000
H	-4.137161000	1.783047000	-1.015401000
H	-4.355790000	0.517682000	-2.237536000
H	-3.392063000	-2.147802000	0.164989000
H	-3.953721000	-1.889269000	-1.493186000
N	-3.640903000	-0.156760000	-0.407091000
C	-0.773600000	-3.195190000	1.260908000
C	-0.608274000	-3.078689000	-0.116452000
C	-0.211196000	-2.227325000	2.092252000
H	-1.038726000	-3.822643000	-0.779878000
H	-0.328893000	-2.302721000	3.168883000
C	0.100008000	-2.007926000	-0.653815000
C	0.503599000	-1.164400000	1.552640000
H	0.210690000	-1.927230000	-1.731773000
H	0.938013000	-0.420652000	2.215114000
C	0.673046000	-1.025216000	0.166316000
H	-1.328210000	-4.027161000	1.681517000
C	1.415355000	0.126120000	-0.408763000
H	1.210144000	1.045008000	0.149448000
H	1.140691000	0.295011000	-1.453381000
Zn	3.384749000	-0.048819000	-0.377668000
Br	5.711265000	-0.066478000	-0.374388000

TS

C	-0.772630000	0.999773000	-4.113804000
C	-1.663916000	1.489322000	-3.163360000
C	-0.038206000	-0.148350000	-3.842140000
H	-2.250298000	2.377405000	-3.372221000
H	0.650854000	-0.543670000	-4.580483000
C	-1.813874000	0.839157000	-1.945516000
C	-0.192450000	-0.802029000	-2.624548000
H	-2.531501000	1.228365000	-1.230750000
H	0.377163000	-1.703331000	-2.421623000
C	-1.070426000	-0.309128000	-1.656309000
H	-0.659585000	1.507509000	-5.065593000
C	-1.169870000	-1.072978000	-0.380185000
H	-0.827519000	-2.101376000	-0.489640000
C	0.477062000	-1.000822000	2.873689000
C	0.319264000	0.456310000	3.288102000
C	0.221624000	1.338659000	2.051197000
C	-0.894080000	0.881885000	1.119866000
C	-0.649301000	-1.422818000	1.942266000
H	0.474806000	-1.661784000	3.745120000
H	1.435664000	-1.140842000	2.361015000
H	1.159008000	0.769126000	3.915037000
H	-0.589536000	0.568960000	3.893524000
H	0.034858000	2.381021000	2.325193000
H	1.170956000	1.313115000	1.503650000
H	-0.866221000	1.461525000	0.198882000
H	-1.873515000	1.049901000	1.594312000
H	-0.488653000	-2.438917000	1.574510000
H	-1.603973000	-1.413127000	2.492589000
N	-0.756195000	-0.532381000	0.790521000
C	-3.152957000	-4.434880000	-3.601645000
C	-2.794775000	-4.856158000	-2.324145000
C	-3.515319000	-3.106663000	-3.806586000
H	-2.511148000	-5.889075000	-2.151367000
H	-3.797599000	-2.766683000	-4.797469000
C	-2.798588000	-3.961270000	-1.262254000
C	-3.518379000	-2.206306000	-2.749401000
H	-2.521935000	-4.302432000	-0.268651000
H	-3.800835000	-1.172402000	-2.922349000
C	-3.163433000	-2.619311000	-1.456035000
H	-3.152870000	-5.136049000	-4.428947000
C	-3.076122000	-1.657484000	-0.330505000
H	-3.407131000	-0.627700000	-0.460307000
H	-2.986184000	-2.049348000	0.684538000
Zn	-5.208145000	-1.917813000	-0.024556000
Br	-7.493244000	-2.270553000	0.090215000

Products

C	-3.689275000	3.496964000	-1.284643000
C	-2.773294000	2.734275000	-1.998775000
C	-4.027552000	3.123850000	0.013567000
H	-2.503832000	3.014608000	-3.011750000
H	-4.738613000	3.714095000	0.582294000
C	-2.197674000	1.605358000	-1.419819000
C	-3.452466000	1.995636000	0.584439000
H	-1.491117000	1.025265000	-2.004360000
H	-3.722097000	1.707390000	1.596541000
C	-2.530570000	1.218126000	-0.121571000
H	-4.135740000	4.377558000	-1.734346000
C	-1.975570000	-0.024692000	0.551531000
H	-1.979199000	0.183736000	1.627697000
C	-3.829863000	-3.214377000	1.330653000
C	-3.988702000	-3.798459000	-0.068277000
C	-4.120851000	-2.677596000	-1.092512000
C	-2.967256000	-1.691365000	-0.969341000
C	-2.686823000	-2.209110000	1.369485000
H	-3.638684000	-4.004627000	2.063828000
H	-4.756604000	-2.709875000	1.628770000
H	-4.853434000	-4.468012000	-0.112191000
H	-3.105568000	-4.405816000	-0.308818000
H	-4.141814000	-3.080642000	-2.110228000
H	-5.064258000	-2.141382000	-0.935030000
H	-3.109216000	-0.862142000	-1.667436000
H	-2.029381000	-2.205554000	-1.255121000
H	-2.622075000	-1.746711000	2.359648000
H	-1.736017000	-2.752273000	1.201642000
N	-2.886445000	-1.155524000	0.383321000
C	2.319631000	2.575202000	1.688283000
C	1.598699000	1.749338000	2.547182000
C	2.122764000	2.463905000	0.318122000
H	1.745680000	1.833797000	3.618368000
H	2.638277000	3.122060000	-0.372946000
C	0.698571000	0.809060000	2.058023000
C	1.213244000	1.501039000	-0.189224000
H	0.162617000	0.173665000	2.755572000

H	0.957211000	1.522458000	-1.247455000
C	0.472754000	0.665005000	0.686680000
H	3.016103000	3.305462000	2.081872000
C	-0.505937000	-0.343079000	0.167136000
H	-0.398421000	-0.447161000	-0.914683000
H	-0.258891000	-1.316683000	0.602560000
Zn	2.932505000	0.174924000	-0.436069000
Br	4.569030000	-1.453507000	-0.544893000

Cartesian coordinates $Zn_2C_{14}H_{15}OBr_2$

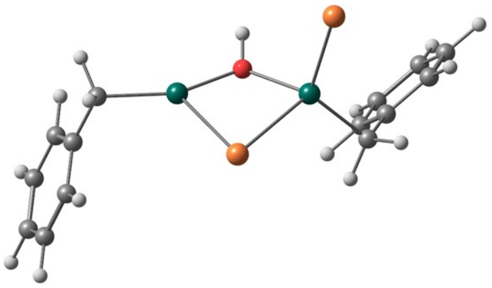

C	3.384517000	-6.115755000	-1.992257000
C	2.124870000	-5.573508000	-1.745720000
C	4.503115000	-5.295047000	-1.864913000
H	1.239780000	-6.197187000	-1.833326000
H	5.494985000	-5.698950000	-2.046637000
C	1.988173000	-4.238985000	-1.382596000
C	4.364060000	-3.960881000	-1.500545000
H	0.996746000	-3.835531000	-1.192450000
H	5.249645000	-3.337941000	-1.403744000
C	3.102981000	-3.392444000	-1.249440000
H	3.492377000	-7.157982000	-2.273672000
C	2.952092000	-1.964412000	-0.890756000
H	3.831580000	-1.588945000	-0.359558000
H	2.070091000	-1.802120000	-0.263719000
Zn	2.715015000	-0.810201000	-2.538988000
Br	4.691248000	0.022036000	-3.802081000
O	1.337270000	-1.176230000	-3.952082000
H	1.605510000	-1.676452000	-4.725247000
C	-5.176542000	0.337826000	-3.286995000
C	-4.693197000	-0.456693000	-4.324038000
C	-4.416253000	1.424517000	-2.862226000
H	-6.130524000	0.115872000	-2.820513000
H	-5.273967000	-1.305360000	-4.673222000
C	-3.471692000	-0.169653000	-4.921875000
H	-4.778566000	2.059073000	-2.058820000
C	-3.195104000	1.709796000	-3.462736000
H	-3.110819000	-0.799625000	-5.730948000
C	-2.689799000	0.919908000	-4.507059000
H	-2.616396000	2.563589000	-3.120120000
C	-1.376475000	1.214212000	-5.136461000
Zn	0.123010000	0.285432000	-4.210026000
H	-1.145142000	2.282366000	-5.094312000
H	-1.359961000	0.899090000	-6.183842000
Br	1.166076000	1.374467000	-2.087750000

Cartesian coordinate, Mannich reaction profile

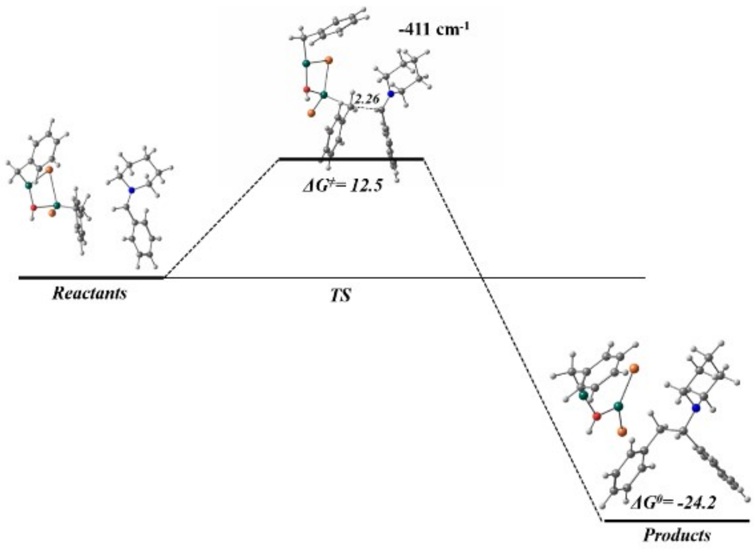

Reactants

C	6.761375000	-1.839341000	1.210848000
C	6.542847000	-1.682401000	-0.155989000
C	5.977333000	-1.149301000	2.130371000
H	7.130840000	-2.248238000	-0.869362000
H	6.133708000	-1.286935000	3.194024000
C	5.568143000	-0.809719000	-0.611868000
C	4.977724000	-0.299620000	1.683801000
H	5.383629000	-0.729217000	-1.675893000
H	4.343847000	0.218315000	2.395936000
C	4.788255000	-0.092484000	0.308515000
H	7.533006000	-2.517159000	1.559093000
C	3.713642000	0.809896000	-0.054528000
H	2.860471000	0.832930000	0.623078000
C	2.625395000	3.846578000	-1.414963000
C	3.678436000	4.144371000	-2.473090000
C	4.950396000	3.367798000	-2.167288000
C	4.695425000	1.869394000	-2.043168000
C	2.375282000	2.351691000	-1.304695000
H	1.673008000	4.326908000	-1.653877000
H	2.947183000	4.231396000	-0.440801000
H	3.890819000	5.215890000	-2.507079000
H	3.299234000	3.861290000	-3.462555000
H	5.698886000	3.505638000	-2.951899000
H	5.393796000	3.728551000	-1.232590000
H	5.597163000	1.356494000	-1.720413000
H	4.372217000	1.446143000	-2.999785000
H	1.684704000	2.110486000	-0.496401000
H	1.959623000	1.961820000	-2.240067000
N	3.626445000	1.606368000	-1.066903000
C	0.639100000	1.285708000	3.803599000
C	0.049588000	1.743018000	2.627345000
C	1.277501000	0.047590000	3.795527000
H	-0.456270000	2.703913000	2.610544000
H	1.745579000	-0.326587000	4.701442000
C	0.097638000	0.977515000	1.467809000
C	1.328321000	-0.715393000	2.634460000
H	-0.384757000	1.346609000	0.565694000
H	1.834981000	-1.676964000	2.648220000
C	0.739226000	-0.275336000	1.435031000
H	0.602193000	1.882664000	4.708632000
C	0.775992000	-1.098312000	0.208131000
H	1.602910000	-1.814058000	0.223861000
H	0.853629000	-0.485125000	-0.695995000
Zn	-0.949160000	-2.140331000	-0.074215000
Br	-0.889198000	-4.401387000	-1.057361000
O	-2.581691000	-1.979324000	1.074372000
H	-2.453230000	-2.078399000	2.020391000
C	-3.182173000	4.902727000	-0.048063000
C	-3.065627000	4.368303000	1.232875000
C	-3.861695000	4.174017000	-1.021219000
H	-2.755454000	5.872295000	-0.282466000
H	-2.545784000	4.924506000	2.007560000
C	-3.616440000	3.127469000	1.532236000
H	-3.968694000	4.576423000	-2.024244000
C	-4.411045000	2.933300000	-0.718704000

H	-3.518311000	2.727372000	2.538155000
C	-4.301572000	2.376164000	0.564802000
H	-4.938852000	2.379289000	-1.490420000
C	-4.875928000	1.043826000	0.882795000
Zn	-3.609446000	-0.450293000	0.512691000
H	-5.763776000	0.840437000	0.276467000
H	-5.158944000	0.975341000	1.937186000
Br	-2.483762000	-0.684271000	-1.816204000

Ts

C	-0.362178000	0.873400000	-3.719551000
C	-1.190779000	1.411090000	-2.737126000
C	0.096275000	-0.432187000	-3.594855000
H	-1.571123000	2.421592000	-2.839341000
H	0.729603000	-0.865003000	-4.361566000
C	-1.544718000	0.655907000	-1.628483000
C	-0.264994000	-1.193757000	-2.489209000
H	-2.219908000	1.081651000	-0.894747000
H	0.089876000	-2.215414000	-2.399773000
C	-1.070785000	-0.653656000	-1.481169000
H	-0.088084000	1.467887000	-4.584425000
C	-1.396713000	-1.540195000	-0.350350000
H	-1.296819000	-2.598822000	-0.572757000
C	-0.450172000	-2.249005000	3.069555000
C	-0.365866000	-0.858524000	3.684786000
C	-0.046939000	0.164488000	2.603470000
C	-1.052643000	0.112494000	1.460122000
C	-1.455122000	-2.275010000	1.929095000
H	-0.752546000	-2.993189000	3.812040000
H	0.532983000	-2.549440000	2.689289000
H	0.394708000	-0.832980000	4.470136000
H	-1.323516000	-0.607973000	4.158985000
H	-0.047207000	1.180112000	3.009352000
H	0.955827000	-0.022661000	2.202013000
H	-0.735061000	0.773086000	0.655908000
H	-2.040263000	0.449323000	1.807103000
H	-1.450787000	-3.243432000	1.425064000
H	-2.469545000	-2.107028000	2.322043000
N	-1.178179000	-1.242776000	0.931452000
C	-3.438023000	-4.145043000	-4.306488000
C	-3.287754000	-4.763803000	-3.068112000
C	-3.643791000	-2.766734000	-4.350930000
H	-3.120379000	-5.835254000	-3.013162000
H	-3.760140000	-2.267850000	-5.308316000
C	-3.347516000	-4.020914000	-1.894672000
C	-3.705865000	-2.020139000	-3.183284000
H	-3.222595000	-4.520538000	-0.936758000
H	-3.875620000	-0.949039000	-3.236345000
C	-3.567119000	-2.628665000	-1.920885000
H	-3.394375000	-4.725647000	-5.221561000
C	-3.610160000	-1.857735000	-0.690805000
H	-3.694712000	-0.777063000	-0.711361000
H	-3.558665000	-2.368088000	0.265765000
Zn	-5.823268000	-1.885434000	-0.323372000
Br	-7.081284000	-0.279014000	-1.592691000
O	-6.344349000	-3.806696000	-0.469669000
H	-5.876035000	-4.303418000	-1.147126000
C	-2.999309000	-6.742422000	4.636057000
C	-3.519063000	-7.560214000	3.635450000
C	-3.728735000	-5.627831000	5.041577000
H	-2.044230000	-6.973477000	5.095901000
H	-2.968436000	-8.438410000	3.311687000
C	-4.743919000	-7.263877000	3.048848000
H	-3.343377000	-4.983234000	5.825972000
C	-4.954718000	-5.334806000	4.453986000
H	-5.135328000	-7.913503000	2.270166000
C	-5.490999000	-6.142766000	3.440610000
H	-5.512181000	-4.462460000	4.784171000
C	-6.791090000	-5.815832000	2.793772000
Zn	-6.553047000	-4.666212000	1.202384000
H	-7.449423000	-5.265389000	3.471150000
H	-7.311066000	-6.719657000	2.464247000
Br	-6.213031000	-1.884133000	2.140240000

Products

C	6.726071000	0.279778000	-0.181294000
C	5.729897000	-0.588925000	-0.611255000
C	6.375526000	1.540096000	0.295634000
H	5.990724000	-1.574510000	-0.983280000
H	7.143531000	2.225152000	0.640180000
C	4.392591000	-0.201919000	-0.564193000
C	5.039785000	1.921293000	0.335259000
H	3.635200000	-0.901952000	-0.900570000
H	4.772839000	2.906828000	0.706776000
C	4.026563000	1.059991000	-0.094220000
H	7.767420000	-0.023362000	-0.213056000
C	2.590766000	1.555531000	-0.060551000
H	2.545378000	2.297957000	0.745208000
C	1.055193000	4.166206000	-2.312809000
C	0.950370000	3.387896000	-3.619376000
C	2.093015000	2.384808000	-3.726087000
C	2.156948000	1.500979000	-2.487324000
C	1.156798000	3.217290000	-1.126337000
H	0.187402000	4.820610000	-2.178238000
H	1.945681000	4.806054000	-2.333694000
H	0.949768000	4.068489000	-4.476951000
H	-0.006402000	2.848659000	-3.642468000
H	1.975260000	1.754263000	-4.613556000
H	3.045409000	2.918719000	-3.830124000
H	3.017469000	0.829639000	-2.553023000
H	1.249299000	0.868070000	-2.454114000
H	1.295563000	3.783782000	-0.199603000
H	0.198731000	2.670463000	-1.030056000
N	2.284909000	2.308786000	-1.281756000
C	1.884296000	-0.829943000	4.404499000
C	1.342003000	0.414599000	4.094924000
C	2.316577000	-1.661763000	3.376833000
H	0.995419000	1.069249000	4.888313000
H	2.736921000	-2.635920000	3.605749000
C	1.237009000	0.820337000	2.769123000
C	2.211214000	-1.249778000	2.051417000
H	0.802297000	1.789709000	2.538585000
H	2.549607000	-1.908800000	1.257507000
C	1.671687000	-0.003764000	1.725790000
H	1.965544000	-1.149417000	5.438218000
C	1.558873000	0.460052000	0.299378000
H	1.642865000	-0.393141000	-0.378839000
H	0.560557000	0.882205000	0.141927000
Zn	-1.084707000	-3.019317000	-0.601097000
Br	-0.022390000	-5.125327000	-0.535433000
O	-1.603634000	-2.174092000	1.098294000
H	-1.367584000	-2.644652000	1.901099000
C	-2.392811000	4.506711000	1.344166000
C	-2.654912000	3.833278000	2.534821000
C	-2.747886000	3.908206000	0.138386000
H	-1.922384000	5.484229000	1.356519000
H	-2.391083000	4.287883000	3.485000000
C	-3.258894000	2.581335000	2.517462000
H	-2.556455000	4.421234000	-0.799314000
C	-3.352641000	2.656008000	0.124174000
H	-3.458159000	2.069979000	3.455655000
C	-3.619730000	1.961669000	1.312355000
H	-3.625617000	2.203724000	-0.825669000
C	-4.253158000	0.613273000	1.295578000
Zn	-2.915771000	-0.838058000	1.267007000
H	-4.876642000	0.473303000	0.408505000
H	-4.873067000	0.450510000	2.181372000
Br	-1.816802000	-1.565554000	-2.329147000

Disubstituted hemiaminal

Cartesian coordinates $ZnC_{21}NH_{22}OBr$

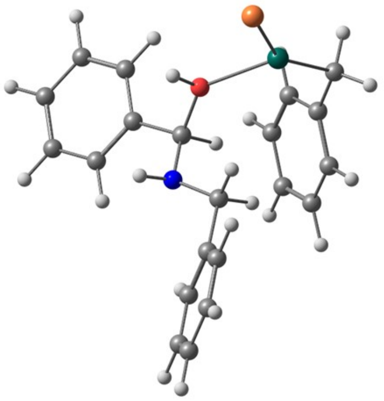

C	-1.739708000	3.747980000	-2.417635000
H	1.414272000	4.984130000	-2.530472000
H	-2.752919000	3.986190000	-2.724062000
C	0.847830000	3.124399000	-1.621969000
C	-1.489009000	2.564925000	-1.731225000
H	1.855313000	2.883497000	-1.303072000
H	-2.305155000	1.888347000	-1.505372000
C	-0.191466000	2.238665000	-1.332159000
H	-0.894364000	5.545144000	-3.246574000
C	0.075153000	0.972606000	-0.544689000
H	-0.109281000	1.144195000	0.520808000
N	1.401075000	0.509970000	-0.707685000
H	1.656183000	0.402907000	-1.683947000
C	1.778625000	-0.649190000	0.087779000
H	1.237447000	-1.559033000	-0.206619000
H	1.491878000	-0.433228000	1.124555000
C	3.260404000	-0.912464000	0.016314000
C	4.178890000	0.102984000	0.294131000
C	3.739996000	-2.179613000	-0.311145000
C	5.545028000	-0.146945000	0.250666000
C	5.108684000	-2.434486000	-0.353792000
C	6.014680000	-1.418416000	-0.072618000
H	3.813634000	1.094598000	0.542314000
H	3.035476000	-2.975214000	-0.536778000
H	6.247061000	0.650755000	0.471210000
H	5.465081000	-3.426454000	-0.611752000
H	7.081497000	-1.613191000	-0.107175000
O	-0.945770000	-0.032555000	-0.869106000
H	-0.880315000	-0.282687000	-1.799840000
C	-0.181850000	2.606635000	3.352707000
C	0.340777000	1.344407000	3.622698000
C	-1.425880000	2.701790000	2.733287000
H	0.368241000	3.501041000	3.625162000
H	1.304961000	1.249898000	4.113313000
C	-0.364906000	0.198812000	3.271156000
H	-1.852916000	3.677430000	2.521397000
C	-2.128589000	1.554965000	2.381917000
H	0.057116000	-0.778387000	3.491488000
C	-1.613598000	0.273721000	2.634576000
H	-3.096086000	1.648397000	1.895451000
C	-2.343073000	-0.949496000	2.215921000
Zn	-1.947665000	-1.484740000	0.330520000
H	-3.426585000	-0.802833000	2.257218000
H	-2.089694000	-1.804690000	2.848425000
Br	-2.208232000	-3.079308000	-1.425819000

Cartesian coordinates S_N1 reaction profile

TS

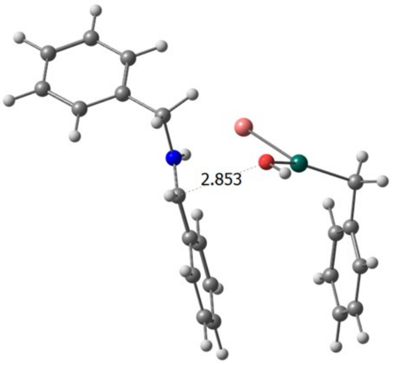

C	1.715940000	0.364868000	5.458179000
C	0.694317000	1.188297000	4.983765000
C	2.348410000	-0.534583000	4.606150000
H	0.205552000	1.886260000	5.653692000
H	3.143148000	-1.172065000	4.975931000
C	0.299950000	1.115862000	3.659572000
C	1.958128000	-0.612684000	3.278034000
H	-0.501527000	1.756080000	3.307102000
H	2.445170000	-1.313090000	2.607423000
C	0.928997000	0.207212000	2.794743000
H	2.019125000	0.428762000	6.497631000
C	0.577794000	0.062547000	1.403501000
H	1.162895000	-0.635569000	0.810748000
N	-0.335830000	0.714010000	0.778191000
H	-1.012653000	1.282539000	1.304660000
C	-0.687799000	0.498606000	-0.622280000
H	-1.697675000	0.082420000	-0.632080000
H	-0.004862000	-0.250723000	-1.025571000
C	-0.623625000	1.782577000	-1.408601000
C	-1.789478000	2.479818000	-1.720040000
C	0.608040000	2.289823000	-1.825556000
C	-1.726689000	3.670083000	-2.438263000
C	0.672165000	3.478115000	-2.542814000
C	-0.496279000	4.170857000	-2.849484000
H	-2.750117000	2.089555000	-1.398125000
H	1.520909000	1.749137000	-1.592972000
H	-2.639881000	4.204468000	-2.677441000
H	1.633592000	3.862082000	-2.866883000
H	-0.446561000	5.097606000	-3.411205000
O	-1.608415000	-1.733715000	1.771319000
H	-1.448986000	-2.659447000	1.969859000
C	-1.780332000	-0.091884000	8.166434000
C	-1.624334000	-1.385054000	7.671360000
C	-2.659191000	0.772282000	7.518110000
H	-1.231478000	0.233747000	9.043872000
H	-0.949496000	-2.077384000	8.166478000
C	-2.329541000	-1.801324000	6.548692000
H	-2.801052000	1.782170000	7.892163000
C	-3.365011000	0.352665000	6.395955000
H	-2.194520000	-2.814492000	6.178128000
C	-3.218227000	-0.944563000	5.876815000
H	-4.048778000	1.040971000	5.906150000
C	-3.946590000	-1.382965000	4.661249000
Zn	-2.859502000	-1.044217000	3.005206000
H	-4.886313000	-0.837129000	4.539705000
H	-4.166964000	-2.454247000	4.697668000
Br	-3.231237000	1.397089000	2.170974000

Products

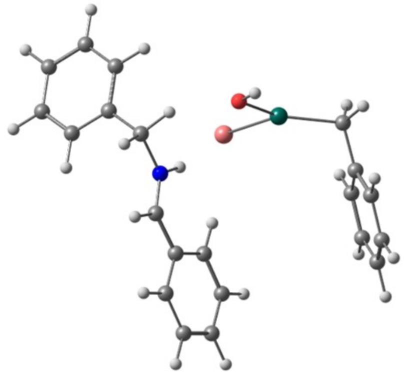

C	2.675754000	0.730694000	5.460898000
C	1.412208000	1.235615000	5.150183000
C	3.414911000	0.041773000	4.504882000
H	0.841276000	1.768472000	5.901934000
H	4.395420000	-0.349889000	4.749138000
C	0.881600000	1.056039000	3.885592000
C	2.890491000	-0.144125000	3.235347000
H	-0.104733000	1.449776000	3.657432000
H	3.459205000	-0.681947000	2.483943000
C	1.621386000	0.360915000	2.914998000
H	3.082973000	0.875725000	6.455648000
C	1.145732000	0.117643000	1.576375000
H	1.792606000	-0.453950000	0.914018000
N	0.024928000	0.500696000	1.075415000
H	-0.660238000	1.034244000	1.638205000
C	-0.436722000	0.178680000	-0.273211000
H	-1.375935000	-0.371763000	-0.132796000
H	0.305082000	-0.483236000	-0.726991000
C	-0.645188000	1.421364000	-1.101897000
C	-1.888305000	1.669728000	-1.680529000
C	0.400221000	2.319969000	-1.322060000
C	-2.084308000	2.795441000	-2.474605000
C	0.204232000	3.448084000	-2.109376000
C	-1.039262000	3.687444000	-2.688542000
H	-2.706502000	0.978387000	-1.505846000
H	1.374673000	2.140174000	-0.877263000
H	-3.056258000	2.977889000	-2.920602000
H	1.023336000	4.140179000	-2.273642000
H	-1.191955000	4.567900000	-3.303668000
O	-2.842001000	-1.137782000	1.176781000
H	-3.119720000	-2.055170000	1.124880000
C	-1.987501000	-0.697406000	7.872815000
C	-2.289217000	-1.865138000	7.174938000
C	-2.552642000	0.502383000	7.447564000
H	-1.328976000	-0.723466000	8.734671000
H	-1.865380000	-2.812739000	7.494645000
C	-3.134390000	-1.830296000	6.072274000
H	-2.336836000	1.422790000	7.982514000
C	-3.399139000	0.534126000	6.344879000
H	-3.357842000	-2.752199000	5.541021000
C	-3.710324000	-0.629850000	5.622518000
H	-3.831542000	1.479998000	6.029895000
C	-4.582131000	-0.591814000	4.423576000
Zn	-3.506377000	-0.329637000	2.746699000
H	-5.293521000	0.237729000	4.473421000
H	-5.142675000	-1.524763000	4.310881000
Br	-2.579490000	2.063116000	2.497761000

Cartesian coordinates reaction profile

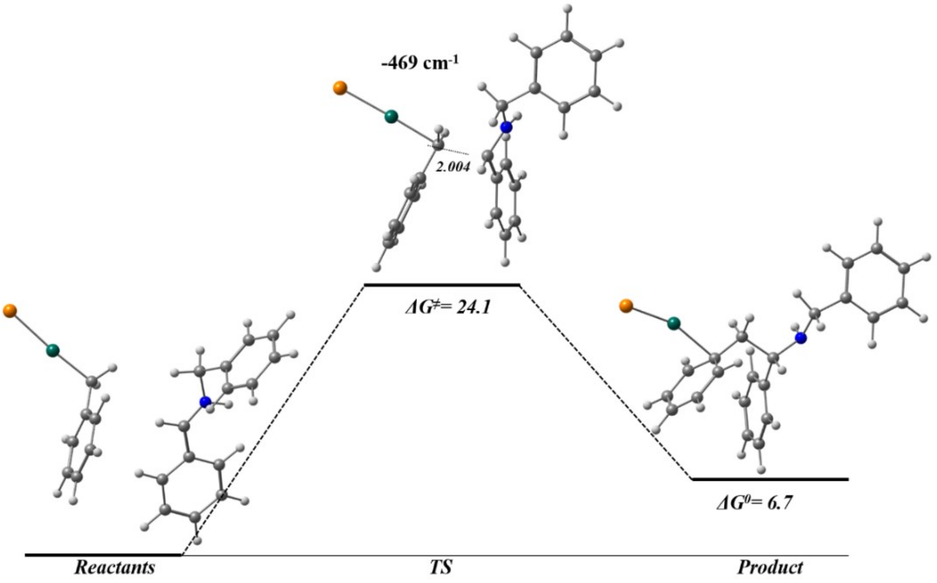

Reactants

C	-3.591812000	4.609004000	-1.111959000
C	-4.281704000	3.569591000	-1.740560000
C	-2.504178000	4.340713000	-0.288125000
H	-5.127502000	3.789320000	-2.381272000
H	-1.971934000	5.150949000	0.196166000
C	-3.887414000	2.260121000	-1.547086000
C	-2.101483000	3.029745000	-0.087428000
H	-4.435864000	1.467734000	-2.045204000
H	-1.251652000	2.810709000	0.552366000
C	-2.789647000	1.979299000	-0.715268000
H	-3.908881000	5.634047000	-1.269978000
C	-2.309120000	0.651898000	-0.452095000
H	-1.447957000	0.552058000	0.206680000
C	0.731107000	2.867281000	2.675680000
C	0.476162000	1.499904000	2.733638000
C	1.350005000	3.390631000	1.542488000
H	-0.005627000	1.073799000	3.608267000
H	1.554344000	4.455058000	1.479257000
C	0.834603000	0.670824000	1.675105000
C	1.703557000	2.560921000	0.484789000
H	0.639023000	-0.396627000	1.743237000
H	2.179886000	2.987255000	-0.393889000
C	1.454915000	1.180607000	0.523962000
H	0.452146000	3.514992000	3.499752000
C	1.831105000	0.296367000	-0.609578000
H	1.804759000	0.832537000	-1.562084000
H	1.168340000	-0.570681000	-0.683307000
Zn	3.659050000	-0.464995000	-0.471178000
Br	5.753472000	-1.494410000	-0.392669000
N	-2.799256000	-0.445865000	-0.920608000
H	-3.604871000	-0.406145000	-1.537888000
C	-2.298819000	-1.791093000	-0.625969000
H	-1.467952000	-1.683033000	0.073016000
H	-1.910312000	-2.209418000	-1.557193000
C	-3.388710000	-2.666433000	-0.065248000
C	-3.852774000	-3.759582000	-0.793845000
C	-3.937457000	-2.394211000	1.189351000
C	-4.851456000	-4.577181000	-0.273733000
C	-4.936996000	-3.208051000	1.706756000
C	-5.394977000	-4.301483000	0.975676000
H	-3.429083000	-3.975633000	-1.769789000
H	-3.580762000	-1.546065000	1.766443000
H	-5.203575000	-5.428441000	-0.846157000
H	-5.356590000	-2.992348000	2.683350000
H	-6.173614000	-4.938035000	1.381962000

Transition State

C	-0.613334000	0.826557000	-4.121146000
C	-1.241179000	1.508438000	-3.080267000
C	-0.165133000	-0.473815000	-3.925320000
H	-1.599126000	2.520926000	-3.231179000
H	0.320909000	-1.011400000	-4.732056000
C	-1.414016000	0.894606000	-1.848327000
C	-0.340710000	-1.092447000	-2.692343000
H	-1.920678000	1.439027000	-1.056545000
H	0.012154000	-2.107187000	-2.540507000
C	-0.955447000	-0.410506000	-1.640391000
H	-0.478620000	1.309968000	-5.082706000
C	-1.093546000	-1.091981000	-0.337038000
H	-0.668235000	-2.093643000	-0.306141000
C	-2.963762000	-4.488178000	-3.726078000
C	-2.535480000	-4.921940000	-2.474468000
C	-3.412818000	-3.179665000	-3.880386000
H	-2.184882000	-5.940123000	-2.342148000
H	-3.748967000	-2.830982000	-4.851223000
C	-2.558920000	-4.059383000	-1.386186000
C	-3.433044000	-2.311094000	-2.797445000
H	-2.232272000	-4.412289000	-0.412090000
H	-3.781879000	-1.291154000	-2.928333000
C	-3.009312000	-2.738353000	-1.530200000
H	-2.950940000	-5.165434000	-4.572991000
C	-2.966782000	-1.801124000	-0.385176000
H	-3.364610000	-0.792630000	-0.502584000
H	-2.805672000	-2.192880000	0.620856000
Zn	-4.997014000	-2.248959000	0.165834000
Br	-7.188132000	-2.763333000	0.700044000
N	-0.862002000	-0.370714000	0.782330000
H	-1.041598000	0.623069000	0.753755000
C	-0.831250000	-0.973205000	2.101945000
H	-0.492656000	-2.007478000	1.979783000
H	-1.836382000	-1.009177000	2.541549000
C	0.097294000	-0.232651000	3.030398000
C	-0.367827000	0.272430000	4.242300000
C	1.440420000	-0.055797000	2.691049000
C	0.494917000	0.939630000	5.108447000
C	2.302109000	0.611138000	3.552373000
C	1.830781000	1.110273000	4.764842000
H	-1.412051000	0.143950000	4.511900000
H	1.809622000	-0.442834000	1.746064000
H	0.120161000	1.328882000	6.049182000
H	3.344325000	0.740334000	3.279883000
H	2.504289000	1.630899000	5.437323000

PRODUCTS

C	-1.020170000	4.760436000	-1.014030000
C	-1.203333000	3.722737000	-1.923857000
C	-0.951221000	4.477776000	0.345514000
H	-1.258158000	3.934561000	-2.986560000
H	-0.809426000	5.279894000	1.062303000
C	-1.314522000	2.411803000	-1.474803000
C	-1.069517000	3.164704000	0.791872000
H	-1.448917000	1.615836000	-2.202338000
H	-1.021232000	2.951619000	1.855816000
C	-1.254043000	2.117576000	-0.110213000
H	-0.931452000	5.783279000	-1.364477000
C	-1.379420000	0.694693000	0.386120000
H	-1.328927000	0.716604000	1.481414000
C	3.291288000	1.888809000	1.348232000
C	2.581583000	1.036135000	2.192962000
C	2.965676000	1.967625000	0.000849000
H	2.853331000	0.958579000	3.239323000
H	3.529539000	2.611678000	-0.663553000
C	1.520778000	0.285690000	1.702944000
C	1.899752000	1.221809000	-0.506417000
H	0.956041000	-0.358890000	2.368670000
H	1.607851000	1.327379000	-1.546370000
C	1.130302000	0.382463000	0.342494000
H	4.110010000	2.479994000	1.741957000
C	-0.191827000	-0.185329000	-0.107111000
H	-0.226860000	-0.255079000	-1.198412000
H	-0.322267000	-1.194233000	0.293831000
Zn	2.704867000	-1.090700000	-0.289882000
Br	4.769257000	-2.129109000	-0.360404000
N	-2.685177000	0.157772000	0.040649000
H	-2.823186000	0.156188000	-0.964712000
C	-3.000495000	-1.151984000	0.596287000
H	-2.835449000	-1.094096000	1.679627000
H	-2.352793000	-1.958848000	0.221062000
C	-4.434704000	-1.526362000	0.322865000
C	-4.744324000	-2.688741000	-0.381124000
C	-5.479206000	-0.715110000	0.773526000
C	-6.069314000	-3.041514000	-0.627989000
C	-6.801799000	-1.063109000	0.529322000
C	-7.101138000	-2.229023000	-0.173205000
H	-3.940643000	-3.325474000	-0.740336000
H	-5.247176000	0.195432000	1.317085000
H	-6.292781000	-3.949627000	-1.178379000
H	-7.603136000	-0.425648000	0.888856000
H	-8.134194000	-2.500250000	-0.364148000

Cartesian coordinates reaction profile

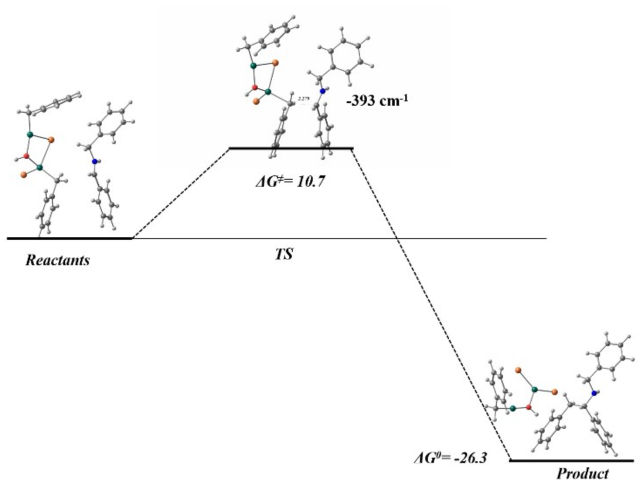

Reactants

C	4.880610000	4.741148000	-0.211511000
C	3.970378000	4.647825000	-1.266402000
C	4.530455000	4.312563000	1.064161000
H	4.251698000	4.985244000	-2.257019000
H	5.239610000	4.386253000	1.880116000
C	2.709600000	4.125425000	-1.050432000
C	3.267009000	3.789458000	1.289228000
H	2.017463000	4.063170000	-1.883347000
H	2.984974000	3.452126000	2.280911000
C	2.346040000	3.690989000	0.235154000
H	5.868093000	5.152261000	-0.390408000
C	1.056379000	3.139400000	0.551034000
H	0.866427000	2.856962000	1.583122000
C	5.823887000	-0.806999000	2.310333000
C	4.628591000	-0.856564000	3.024581000
C	5.778387000	-0.523916000	0.946997000
H	4.641884000	-1.071866000	4.089202000
H	6.698982000	-0.477183000	0.372381000
C	3.414609000	-0.629842000	2.387143000
C	4.563193000	-0.296228000	0.311851000
H	2.492967000	-0.672640000	2.962408000
H	4.549116000	-0.076301000	-0.752559000
C	3.345500000	-0.343360000	1.012391000
H	6.772183000	-0.983497000	2.806933000
C	2.051460000	-0.129995000	0.330855000
H	2.153962000	0.531369000	-0.534185000
H	1.302787000	0.284606000	1.014610000
N	0.079943000	2.956811000	-0.271770000
H	0.203045000	3.200108000	-1.249976000
C	-1.219140000	2.369479000	0.073234000
H	-1.243433000	2.252336000	1.157903000
H	-1.258593000	1.374885000	-0.383746000
C	-2.350611000	3.229636000	-0.422244000
C	-3.051826000	2.865424000	-1.570753000
C	-2.703992000	4.397206000	0.256538000
C	-4.094107000	3.659745000	-2.038344000
C	-3.745092000	5.190057000	-0.210051000
C	-4.440725000	4.822557000	-1.359164000
H	-2.785412000	1.952516000	-2.095137000
H	-2.167728000	4.684924000	1.156163000
H	-4.636646000	3.367553000	-2.930972000
H	-4.017335000	6.093253000	0.325410000
H	-5.254874000	5.441241000	-1.721378000
Br	2.359097000	-3.429159000	-1.855403000
Zn	1.210478000	-1.847287000	-0.349123000
O	0.053502000	-2.942416000	0.874268000
Br	-1.117150000	-1.230281000	-1.547152000
H	0.391784000	-3.773590000	1.212769000
Zn	-1.810276000	-2.830577000	0.481387000
C	-5.696981000	0.568582000	1.972694000
C	-5.234256000	-0.359050000	2.903344000
C	-5.539129000	0.296298000	0.616270000
H	-6.176793000	1.485233000	2.299198000
H	-5.354791000	-0.168243000	3.965673000
C	-4.622070000	-1.534049000	2.483214000
H	-5.897581000	1.004998000	-0.124491000
C	-4.927987000	-0.880949000	0.198890000
H	-4.268176000	-2.247296000	3.223331000
C	-4.450856000	-1.825362000	1.120739000

H	-4.815117000	-1.078135000	-0.863744000
C	-3.778868000	-3.073180000	0.675243000
H	-4.138395000	-3.390912000	-0.307630000
H	-3.944671000	-3.889195000	1.384268000

TS

C	-0.664834000	1.342115000	-3.871130000
C	-1.143487000	1.859284000	-2.667190000
C	-0.334911000	-0.004282000	-3.963150000
H	-1.410427000	2.907826000	-2.594183000
H	0.035044000	-0.412875000	-4.897125000
C	-1.284270000	1.037218000	-1.561073000
C	-0.478960000	-0.834377000	-2.857531000
H	-1.679061000	1.459897000	-0.642254000
H	-0.214469000	-1.883789000	-2.928311000
C	-0.945332000	-0.321126000	-1.641727000
H	-0.554329000	1.990431000	-4.733633000
C	-1.052408000	-1.226252000	-0.502903000
H	-0.732509000	-2.251387000	-0.660588000
C	-2.664508000	-4.425059000	-4.311352000
C	-2.357313000	-4.934882000	-3.050848000
C	-3.145034000	-3.121641000	-4.413999000
H	-1.980049000	-5.948332000	-2.953084000
H	-3.388122000	-2.708760000	-5.388316000
C	-2.531453000	-4.158660000	-1.913488000
C	-3.315119000	-2.338746000	-3.280085000
H	-2.295444000	-4.574639000	-0.937708000
H	-3.694330000	-1.325818000	-3.376143000
C	-3.018995000	-2.839934000	-1.998246000
H	-2.532088000	-5.034818000	-5.198638000
C	-3.166873000	-2.020399000	-0.809340000
H	-3.418849000	-0.968640000	-0.888333000
H	-2.964472000	-2.450711000	0.166044000
N	-0.945841000	-0.779152000	0.746949000
H	-1.099329000	0.203905000	0.925310000
C	-1.017773000	-1.655661000	1.902649000
H	-0.642351000	-2.636042000	1.592193000
H	-2.064026000	-1.787885000	2.208466000
C	-0.209046000	-1.125349000	3.057420000
C	-0.806743000	-0.914882000	4.298077000
C	1.151586000	-0.851547000	2.902223000
C	-0.057070000	-0.443003000	5.372443000
C	1.900085000	-0.376906000	3.971878000
C	1.296774000	-0.172435000	5.210862000
H	-1.865567000	-1.120571000	4.424933000
H	1.624668000	-1.010781000	1.937791000
H	-0.533932000	-0.282817000	6.333622000
H	2.956691000	-0.168346000	3.840691000
H	1.881792000	0.197778000	6.046106000
Br	-6.905843000	-1.118759000	-1.528091000
Zn	-5.293435000	-2.363740000	-0.257077000
O	-5.609354000	-4.272997000	0.119636000
Br	-5.304778000	-1.995681000	2.275136000
H	-5.957334000	-4.862151000	-0.552138000
Zn	-5.943409000	-4.621967000	1.970684000
C	-2.590735000	-6.070759000	5.898931000
C	-2.923927000	-7.021974000	4.937743000
C	-3.458477000	-5.004087000	6.119397000
H	-1.672374000	-6.161500000	6.469351000
H	-2.263316000	-7.864659000	4.756615000
C	-4.101778000	-6.904899000	4.208663000
H	-3.218769000	-4.255882000	6.869132000
C	-4.636387000	-4.890282000	5.389512000
H	-4.346724000	-7.657389000	3.463472000
C	-4.986939000	-5.835602000	4.413582000
H	-5.302504000	-4.051896000	5.575330000
C	-6.234695000	-5.699984000	3.616311000
H	-7.018892000	-5.192804000	4.185206000
H	-6.613930000	-6.675540000	3.299647000

Produts

C	5.366234000	-4.291476000	-0.337383000
C	5.011936000	-3.312787000	-1.261883000
C	4.988121000	-4.146508000	0.992927000
H	5.301653000	-3.417675000	-2.302426000
H	5.258129000	-4.903981000	1.721668000
C	4.282083000	-2.200475000	-0.857328000
C	4.263388000	-3.027791000	1.394087000
H	4.007547000	-1.453316000	-1.596892000
H	3.974438000	-2.918824000	2.435502000
C	3.901581000	-2.041344000	0.477407000
H	5.932537000	-5.161389000	-0.653217000
C	3.127504000	-0.821513000	0.928187000
H	2.917953000	-0.940898000	1.998271000

C	-0.837891000	-4.061048000	0.927964000
C	-0.611286000	-3.114670000	1.923968000
C	-0.220711000	-3.911883000	-0.310128000
H	-1.090421000	-3.219777000	2.892204000
H	-0.389614000	-4.642568000	-1.094556000
C	0.222091000	-2.027979000	1.680976000
C	0.619494000	-2.825534000	-0.547230000
H	0.380801000	-1.289434000	2.462097000
H	1.105449000	-2.721424000	-1.513227000
C	0.852522000	-1.866869000	0.443503000
H	-1.490009000	-4.907450000	1.115635000
C	1.766874000	-0.699387000	0.199163000
H	1.944492000	-0.581565000	-0.874961000
H	1.275536000	0.216364000	0.543580000
N	3.979055000	0.360232000	0.806683000
H	4.222684000	0.519825000	-0.166166000
C	3.436428000	1.582552000	1.386604000
H	3.170982000	1.358423000	2.427517000
H	2.516738000	1.935713000	0.895991000
C	4.452369000	2.695383000	1.354418000
C	4.194597000	3.875722000	0.659402000
C	5.672796000	2.562445000	2.021902000
C	5.130704000	4.907413000	0.632767000
C	6.608773000	3.588746000	1.998895000
C	6.340064000	4.766193000	1.302984000
H	3.250921000	3.989459000	0.133115000
H	5.885176000	1.643728000	2.559940000
H	4.913917000	5.819238000	0.085762000
H	7.550432000	3.473176000	2.526003000
H	7.071416000	5.567524000	1.284528000
Br	0.003385000	0.451852000	-3.890539000
Zn	-1.695691000	0.624398000	-2.252924000
O	-2.121020000	-0.998312000	-1.263193000
Br	-3.165709000	2.359787000	-1.571441000
H	-1.395812000	-1.627186000	-1.166717000
Zn	-3.456684000	-1.139682000	0.052127000
C	-4.267456000	1.700099000	4.555807000
C	-3.790379000	0.397835000	4.679453000
C	-4.958833000	2.058860000	3.401571000
H	-4.106457000	2.423173000	5.348339000
H	-3.255249000	0.098703000	5.575646000
C	-3.998972000	-0.528072000	3.663306000
H	-5.342826000	3.068363000	3.290349000
C	-5.165549000	1.130919000	2.387267000
H	-3.622706000	-1.541467000	3.776732000
C	-4.689391000	-0.183565000	2.492753000
H	-5.707686000	1.426997000	1.493115000
C	-4.901426000	-1.171937000	1.399861000
H	-5.826542000	-0.970935000	0.852516000
H	-4.947845000	-2.193542000	1.786550000
